# Selenium and Vitamin E for Prevention of Non–Muscle-Invasive Bladder Cancer Recurrence and Progression: A Randomized Clinical Trial

**DOI:** 10.1001/jamanetworkopen.2023.37494

**Published:** 2023-10-17

**Authors:** Richard T. Bryan, Sarah J. Pirrie, Ben Abbotts, Shanna Maycock, Vinnie During, Carolyn Lewis, Margaret Grant, Deborah Bird, Adam J. Devall, D. Michael A. Wallace, Nicholas D. James, Lucinda J. Billingham, Maurice P. Zeegers, K. K. Cheng

**Affiliations:** 1 Bladder Cancer Research Centre, Institute of Cancer and Genomic Sciences, University of Birmingham, Birmingham, United Kingdom; 2 Cancer Research UK Clinical Trials Unit, Institute of Cancer and Genomic Sciences, University of Birmingham, United Kingdom; 3 Department of Urology, The Royal Wolverhampton NHS Trust, Wolverhampton, United Kingdom; 4 MAC Clinical Research, Lancashire, United Kingdom; 5 Birmingham Clinical Trials Unit, Institute of Applied Health Research, University of Birmingham, United Kingdom; 6 Institute of Metabolism and Systems Research, University of Birmingham, United Kingdom; 7 Department of Urology, University Hospitals Birmingham NHS Foundation Trust, Birmingham, United Kingdom; 8 Prostate and Bladder Cancer Research Team, The Institute of Cancer Research, London, United Kingdom; 9 Department of Epidemiology, Maastricht University, Maastricht, the Netherlands; 10 MPB holding, Heerlen, the Netherlands; 11 Institute of Applied Health Research, University of Birmingham, United Kingdom

## Abstract

**Importance:**

Selenium and vitamin E have been identified as promising agents for the chemoprevention of recurrence and progression of non–muscle-invasive bladder cancer.

**Objective:**

To determine whether selenium and/or vitamin E may prevent disease recurrence in patients with newly diagnosed NMIBC.

**Design, Setting, and Participants:**

This multicenter, prospective, double-blinded, placebo-controlled, 2 × 2 factorial randomized clinical trial included patients with newly diagnosed NMIBC recruited from 10 secondary or tertiary care hospitals in the UK. A total of 755 patients were screened for inclusion; 484 did not meet the inclusion criteria, and 1 declined to participate. A total of 270 patients were randomly assigned to 4 groups (selenium plus placebo, vitamin E plus placebo, selenium plus vitamin E, and placebo plus placebo) in a double-blind fashion between July 17, 2007, and October 10, 2011. Eligibility included initial diagnosis of NMIBC (stages Ta, T1, or Tis); randomization within 12 months of first transurethral resection was required.

**Interventions:**

Oral selenium (200 μg/d of high-selenium yeast) and matched vitamin E placebo, vitamin E (200 IU/d of d-alfa-tocopherol) and matched selenium placebo, selenium and vitamin E, or placebo and placebo.

**Main Outcome and Measures:**

Recurrence-free interval (RFI) on an intention-to-treat basis (analyses completed on November 28, 2022).

**Results:**

The study randomized 270 patients (mean [SD] age, 68.9 [10.4] years; median [IQR] age, 69 [63-77] years; 202 male [75%]), with 65 receiving selenium and vitamin E placebo, 71 receiving vitamin E and selenium placebo, 69 receiving selenium and vitamin E, and 65 receiving both placebos. Median overall follow-up was 5.5 years (IQR, 5.1-6.1 years); 228 patients (84%) were followed up for more than 5 years. Median treatment duration was 1.5 years (IQR, 0.9-2.5 years). The study was halted because of slow accrual. For selenium (n = 134) vs no selenium (n = 136), there was no difference in RFI (hazard ratio, 0.92; 95% CI, 0.65-1.31; *P* = .65). For vitamin E (n = 140) vs no vitamin E (n = 130), there was a statistically significant detriment to RFI (hazard ratio, 1.46; 95% CI, 1.02-2.09; *P* = .04). No significant differences were observed for progression-free interval or overall survival time with either supplement. Results were unchanged after Cox proportional hazards regression modeling to adjust for known prognostic factors. In total, 1957 adverse events were reported; 85 were serious adverse events, and all were considered unrelated to trial treatment.

**Conclusions and Relevance:**

In this randomized clinical trial of selenium and vitamin E, selenium supplementation did not reduce the risk of recurrence in patients with NMIBC, but vitamin E supplementation was associated with an increased risk of recurrence. Neither selenium nor vitamin E influenced progression or overall survival. Vitamin E supplementation may be harmful to patients with NMIBC, and elucidation of the underlying biology is required.

**Trial Registration:**

isrctn.org Identifier: ISRCTN13889738

## Key Points

**Question:**

Can selenium and vitamin E supplementation prevent recurrence and progression of non–muscle-invasive bladder cancer (NMIBC)?

**Findings:**

In this randomized clinical trial of 270 adults, supplementation with selenium was not associated with a decreased risk of NMIBC recurrence; vitamin E supplementation was associated with a significantly increased risk of recurrence. Neither selenium nor vitamin E was associated with progression or overall survival.

**Meaning:**

These findings suggest that vitamin E supplements may be harmful to patients with NMIBC.

## Teaser

This randomized clinical trial assesses the ability of selenium and vitamin E to prevent non–muscle-invasive bladder cancer (NMIBC) recurrence and progression in patients with newly diagnosed disease.

## Introduction

Bladder cancer is the 12th most common cancer worldwide^1^; in high-income countries, more than 90% are transitional cell carcinomas of urothelial origin, and most patients (75%-85%) present with non–muscle-invasive bladder cancer (NMIBC) (Union for International Cancer Control stages Ta, T1, and Tis).^2^ Patients with NMIBC are initially treated by transurethral resection of bladder tumor and adjuvant intravesical therapy.^3^ Recurrence occurs in up to 80% of patients^4^; progression to muscle-invasive bladder cancer (stages T2 or higher) occurs in up to 45% of patients diagnosed with initial stage T1 disease.^5^

Given the frequency of recurrence and progression and the chronic nature of the disease, NMIBC may be amenable to chemoprevention.^6^ Selenium and vitamin E have previously been identified as promising agents^7,8,9^; notwithstanding, more recent clinical trials of selenium and/or vitamin E in the primary and secondary cancer prevention settings have shown no effect^10,11^ or a detrimental effect.^12^ Between 2005 and 2007, we established a trial protocol with the aim of providing important insights into the use of selenium and vitamin E as adjuvant therapies for NMIBC (SELENIB trial).

## Methods

### Study Design and Participants

From July 17, 2007, to October 10, 2011, SELENIB recruited patients with newly diagnosed NMIBC to a double-blinded, placebo-controlled, 2 × 2 factorial randomized clinical trial from 10 UK hospitals. Eligible patients were 18 years or older with newly diagnosed, pathologically confirmed urothelial NMIBC who were able to give informed consent. Ethnicity data were not collected because they were not considered to be relevant for a study of this nature. Patients were required to be randomized within 12 months of initial transurethral resection of bladder tumor. All patients provided written informed consent. SELENIB was coordinated by the Cancer Research UK Clinical Trials Unit at the University of Birmingham. The trial was conducted in accordance with the principles of the Good Clinical Practice guidelines and the Declaration of Helsinki.^13^ It was approved by the UK Medicines and Healthcare Products Regulatory Agency. Research ethics approval was gained from East Midlands–Derby Research Ethics Committee, and the trial was overseen by an independent data monitoring committee. This report follows the Consolidated Standards of Reporting Trials (CONSORT) reporting guideline.^14^ The trial protocol can be found in Supplement 1.

### Randomization, Blinding, and Interventions

Patients were randomly assigned equally to 1 of 4 groups: oral selenium (200 μg/d of high selenium yeast, 364% recommended daily allowance^15^) and matched vitamin E placebo, vitamin E (200 IU/d d-alfa-tocopherol, 600% recommended daily allowance^16^) and matched selenium placebo, selenium and vitamin E, or placebo and placebo. Treatment allocations were blinded. Randomization was stratified by recurrence risk group (high vs low or intermediate^17^) and treatment center. Patients took 1 tablet (selenium or placebo) and 1 capsule (vitamin E or placebo) once daily with food for up to 5 years. Patients were otherwise treated according to contemporaneous European Association of Urology guidelines.^17^ Patients attended a SELENIB follow-up clinic every 6 months for up to 5 years after randomization during which treatment adherence, toxic effects, and disease status were recorded.

### Outcomes

The primary outcome was recurrence-free interval (RFI), measured as the time from study entry to recurrence. For patients without recurrence at the time of analysis, the interval was censored at the date the patient was last known to be recurrence free. Recurrences at the first 3-month cystoscopy checkup were excluded. Secondary outcome measures included progression-free interval and overall survival (eMethods in Supplement 2). Quality of life was assessed at each follow-up visit.

### Statistical Analysis

The primary hypothesis was addressed on an intention-to-treat basis. As a 2 × 2 factorial design, analysis consisted of 2 comparisons: (1) those patients randomized to selenium vs those randomized to the associated placebo, stratifying by vitamin E allocation; and (2) those patients randomized to vitamin E vs those randomized to the associated placebo, stratifying by selenium allocation. Interaction was not expected. Treatment groups were compared by Kaplan-Meier estimates of recurrence-free and progression-free interval; log-rank tests were used to test the hypothesis of no difference between treatments. Hazard ratios (HRs) from Cox proportional hazards regression models compared treatments, both unadjusted and adjusted for known prognostic factors. Similar methods were used for secondary outcomes. These analyses were completed on November 28, 2022.

All tests used a 2-sided *P* < .05 to indicate statistical significance, and all analysis was performed in R software, version 4.2.0 (R Foundation for Statistical Computing) using lmtest, survival, survminer and cowplot, ggplot2, and RColorBrewer for graphs.

## Results

### Participants

**Figure 1.**
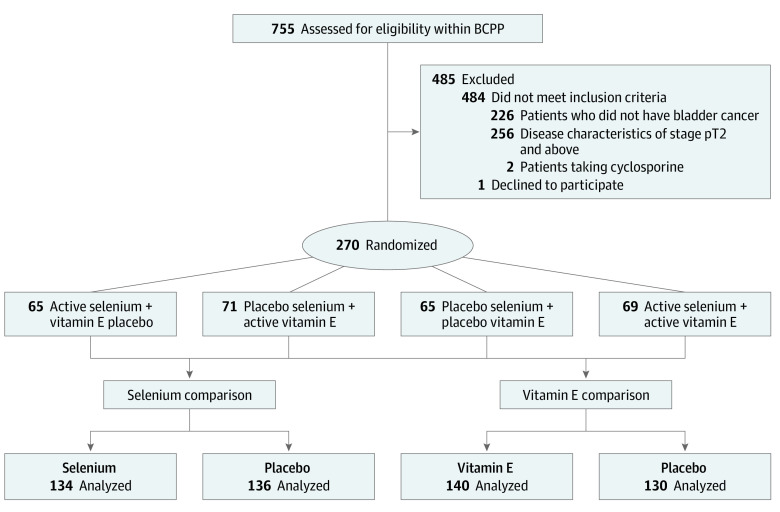
SELENIB Trial Profile Trial profile of the 4 arms and comparisons analyzed within the SELENIB trial. BCPP indicates Bladder Cancer Prognosis Programme.

The study randomized 270 patients (mean [SD] age, 68.9 [10.4] years; median [IQR] age, 69 [63-77] years; 202 male [75%] and 68 female [25%]), with 65 receiving selenium and vitamin E placebo, 71 receiving vitamin E and selenium placebo, 69 receiving selenium and vitamin E, and 65 receiving both placebos (Figure 1). Baseline characteristics are shown in the Table. A total of 256 (95%) of all tumors were pure transitional cell carcinomas, and 226 (84%) had papillary morphology (eTable 1 in Supplement 2). The trial failed to recruit to its prespecified target of 515 patients and was halted because of slow accrual. Two hundred twenty-eight patients were followed up for more than 5 years. To September 26, 2018, median overall follow-up was 5.5 years (IQR, 5.1-6.1 years); with the intervention of the COVID-19 pandemic, ongoing follow-up beyond this time point became unfeasible. All patients are included in the analysis.

**Table.** Baseline Characteristics of the Study Participants^a^

Characteristic	Selenium		Vitamin E		Overall (N = 270)
	Placebo (n = 136)	Active (n = 134)	Placebo (n = 130)	Active (n = 140)	
Age, y					
Median (IQR)	69.5 (63.0-76.2)	69.0 (62.0-77.0)	69.0 (62.2-75.8)	69.5 (63.0-78.0)	69.0 (63.0-77.0)
Mean (SD)	69.0 (10.3)	68.8 (10.6)	68.3 (10.0)	69.5 (10.9)	68.9 (10.4)
Sex					
Female	27 (20)	26 (19)	28 (22)	25 (18)	53 (20)
Male	102 (75)	100 (75)	97 (75)	105 (75)	202 (75)
Unknown	7 (5)	8 (6)	5 (4)	10 (7)	15 (6)
Baseline risk group^b^					
Low	17 (12)	21 (16)	18 (14)	20 (14)	38 (14)
Intermediate	59 (43)	55 (41)	53 (41)	61 (44)	114 (42)
High	51 (38)	53 (40)	48 (37)	56 (40)	104 (39)
Very high	9 (7)	5 (4)	11 (8)	3 (2)	14 (5)
Grade					
1	23 (17)	31 (23)	28 (22)	26 (19)	54 (20)
2	59 (43)	49 (37)	48 (37)	60 (43)	108 (40)
3	53 (39)	54 (40)	54 (42)	53 (38)	107 (40)
Unknown	1 (0.7)	0	0	1 (0.7)	1 (0.4)
Stage					
pTa	81 (60)	79 (59)	74 (57)	86 (61)	160 (59)
pTis	7 (5)	7 (5)	6 (5)	8 (6)	14 (5.2)
pT1	47 (35)	48 (36)	50 (38)	45 (32)	95 (35)
Unknown	1 (0.7)	0	0	1 (0.7)	1 (0.4)
Carcinoma in situ					
Yes	19 (14)	16 (12)	21 (16)	14 (10)	35 (13)
No	75 (55)	74 (55)	65 (50)	84 (60)	149 (55)
Unknown	42 (31)	44 (33)	44 (34)	42 (30)	86 (32)
No. of tumors					
No.	133	128	128	133	261
Median (IQR)	1.0 (1.0-2.0)	1.5 (1.0-3.0)	1.0 (1.0-3.0)	2.0 (1.0-3.0)	1.0 (1.0-3.0)
Mean (SD)	2.0 (1.9)	2.3 (1.9)	2.0 (1.8)	2.2 (2.0)	2.1 (1.9)
Unknown	3	6	2	7	9
Size of largest tumor, cm					
No.	128	124	123	129	252
Median (IQR)	2.5 (1.5-3.0)	2.0 (1.0-3.0)	2.5 (1.2-3.2)	2.0 (1.5-3.0)	2.0 (1.5-3.0)
Mean (SD)	2.9 (2.0)	2.6 (1.7)	2.8 (2.2)	2.6 (1.6)	2.7 (1.9)
Unknown	8	10	7	11	18

^a^ Data are presented as number (percentage) of patients unless otherwise indicated. Baseline risk groups are not the same as those used for stratification.

^b^ In calculating baseline risk group, carcinoma in situ is assumed absent if not confirmed present.

### Treatment

Across 3 approaches to assess adherence (returned tablets or capsules, diary, and patient recollection), the median percentage of days taking trial treatment was more than 95% (IQR, 90%-99%) (eTables 2 and 3 in Supplement 2). The median treatment duration was 1.5 years (IQR, 0.9-2.5 years). Of 270 participants, 264 (98%) had more than 3 years of follow-up or died within 3 years of randomization. Standard-of-care treatments received by participants are detailed in eTables 4 to 6 in Supplement 2.

### Adverse Events

In total, 1957 adverse events were reported; 85 were serious adverse events, and all were considered unrelated to trial treatment. The most common adverse events were fatigue (279 [14%]), cough and/or cold (217 [11%]), and dermatitis (190 [10%]); there was no difference in adverse events between treatment arms (eResults and eTables 7-9 in Supplement 2).

### Primary Outcome

**Figure 2.**
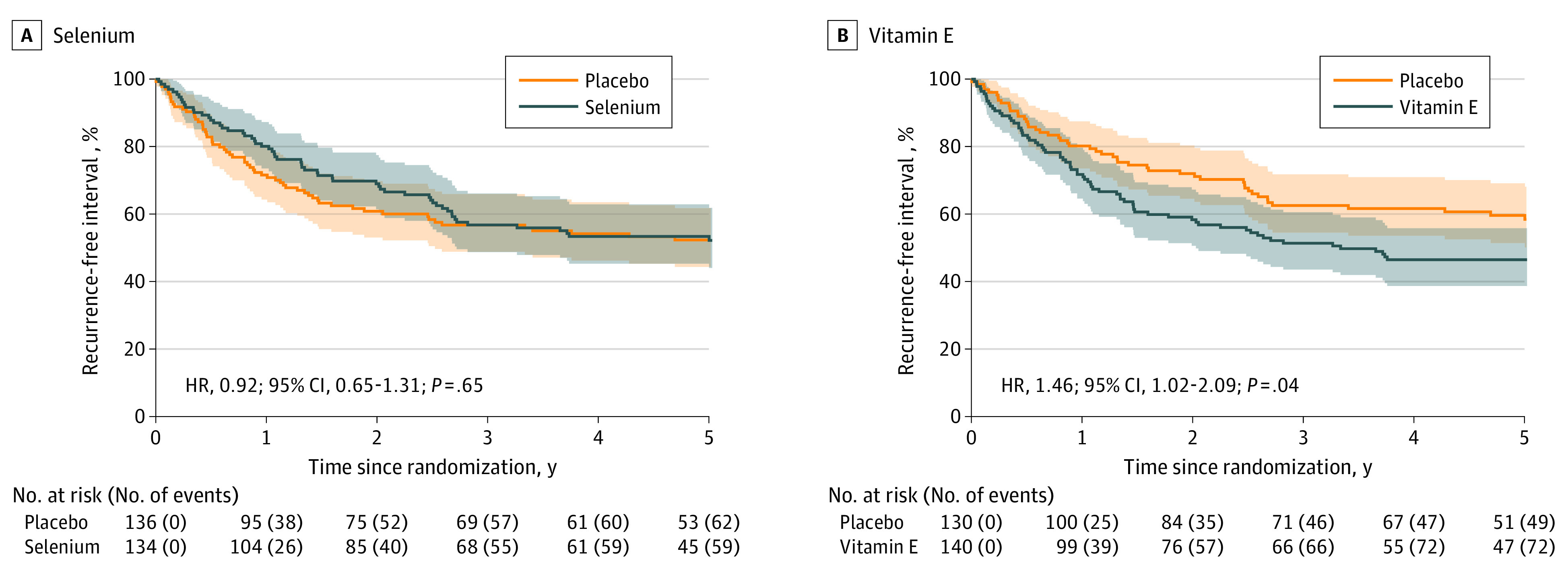
Recurrence-Free Interval Kaplan-Meier 5-year analyses of recurrence-free intervals, defined as the time from date of study entry to date of recurrence, for selenium and vitamin E. For patients not observed to have experienced recurrence at the time of analysis, the interval was censored at the date last known to be recurrence free. HR indicates hazard ratio.

Of 122 recurrences, 60 (49%) occurred in the selenium arm and 62 (51%) in the placebo arm. For selenium, there was no statistically significant difference in RFI (HR, 0.92; 95% CI, 0.65-1.31; *P* = .65); median RFI was not reached in either arm (Figure 2A). Of the recurrences, 72 (59%) occurred in the vitamin E arm and 50 (41%) in the placebo arm. Vitamin E was associated with a statistically significant decrease in RFI (HR, 1.46; 95% CI, 1.02-2.09; *P* = .04); median RFI was 3.3 years (IQR, 0.89 years to not reached) for vitamin E and was not reached for the placebo arm (Figure 2B). Recurrence-free survival estimates at yearly intervals for each treatment comparison are shown in eTable 10 in Supplement 2. We observed a 13% difference in RFI at 5 years for placebo vs vitamin E (59.6% vs 46.5%), with the study originally designed for an absolute difference of 12% at 5 years. Adjusted analyses of the primary outcome were also undertaken (eTables 11 and 12 in Supplement 2).

### Secondary Outcomes

**Figure 3.**
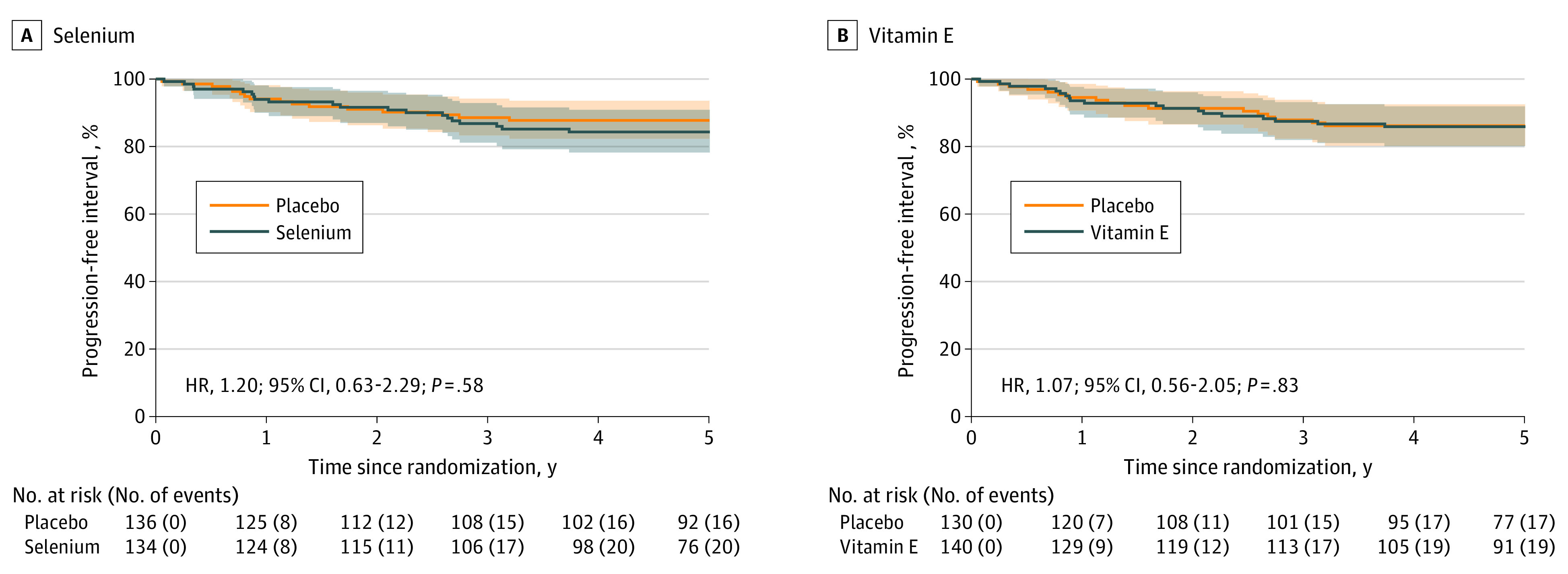
Progression-Free Interval Kaplan-Meier 5-year analyses of progression-free intervals as time from date of study entry to date of progression, for selenium and vitamin E. Progression was defined as recurrence with an increase in grade from grade 1 or grade 2 to grade 3 or an increase in T stage (determined by histopathologic analysis) or the new occurrence of carcinoma in situ (CIS) in a bladder previously free from CIS or the new occurrence of multiple urothelial tumors following the initial diagnosis of a solitary urothelial tumor. Progression was also reported if there was the need for a cystectomy because of refractory disease or the new development of nodal and/or distant metastases (determined by imaging). For those patients not observed to have experienced progression by the time of analysis, the interval was censored at the date last known to be progression free. HR indicates hazard ratio.

**Figure 4.**
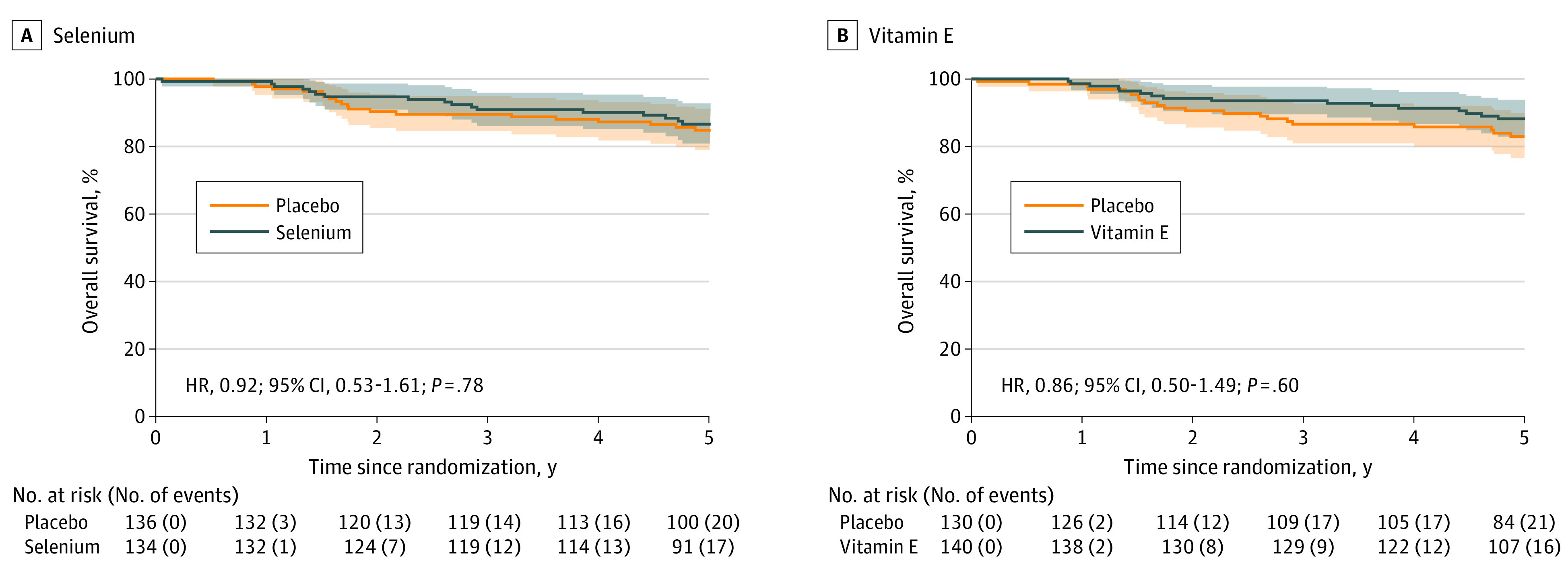
Overall Survival Analysis Kaplan-Meier 5-year analyses of overall survival time defined as time from the date of randomization to the date of death from any cause, for selenium and vitamin E. Patients alive at the time of analysis were censored at the date last known to be alive. For patients not observed to have experienced recurrence at the time of analysis, the interval was censored at the date last known to be recurrence free. HR indicates hazard ratio.

Overall, 37 patients had disease progression; no significant differences in progression-free interval were observed with selenium or vitamin E (Figure 3). Fifty-three patients died, and no significant differences in overall survival were observed with either selenium or vitamin E (Figure 4). No significant differences in quality of life were observed between the arms (eFigure in Supplement 2).

## Discussion

We observed no benefit of selenium for recurrence, progression, or overall survival in patients with NMIBC. We observed an association between vitamin E supplementation and an increased risk of recurrence but no association with progression or overall survival. The assumptions of the factorial trial design were demonstrated to be correct. The randomization used contemporary risk categorization as a stratification factor; over time, risk categorization has changed.^3,17^ For enabling up-to-date risk categorization and applicability to current practice, data were collected on each of the contributory factors.

SELENIB is, to our knowledge, the first trial to investigate the use of selenium and vitamin E for preventing recurrence and progression in patients newly diagnosed with NMIBC. In 2011, the Selenium and Vitamin E Cancer Prevention Trial (SELECT) concluded that dietary supplementation with vitamin E (400 IU/d all-rac-α-tocopheryl acetate) significantly increased the risk of prostate cancer among healthy men^12^; a secondary analysis in 2012 investigated bladder cancer incidence, demonstrating no preventive effect of selenium or vitamin E alone or combined on bladder cancer incidence within the SELECT population.^10^

Trials of dietary supplements in cancer prevention are important, as demonstrated here. Our data show no evidence of benefit from selenium and evidence of harm from vitamin E—a compound readily available over the counter. Future micronutrient and/or vitamin chemoprevention studies in NMIBC should carefully consider choice of compound, underlying biology, preclinical evidence, and study design.^18,19^

### Limitations

This study has some limitations, including the fact that patient accrual fell below the intended 515 due to an unexpectedly high proportion of ineligible patients, the elderly age of the patient population with accompanying comorbidities and medication, and the proposed trial duration. Furthermore, trial funding was withdrawn in 2010, resulting in the decision to end recruitment and treatment in 2011. Consequently, study participants received considerably less selenium and vitamin E than intended, and 245 fewer patients than required were recruited; thus, these analyses are underpowered.

## Conclusions

In this randomized clinical trial of selenium and vitamin E in patients with newly diagnosed NMIBC, selenium supplementation did not reduce the risk of disease recurrence, whereas vitamin E supplementation was associated with an increased risk of recurrence. Neither selenium nor vitamin E influenced progression or overall survival. These findings suggest that vitamin E supplementation may be harmful to patients with NMIBC, and elucidation of the underlying biology is required.

## Notes

Supplement 1.
Trial Protocol

Supplement 2. **eMethods.** Additional Methodological Information **eResults.** Additional Study Results **eTable 1.** Tumour Pathology of Patients Within the SELENIB Trial **eTable 2.** Trial Treatment Compliance **eTable 3.** Trial Treatment Duration **eTable 4.** Overview of Standard Treatments Received by Patients Within the SELENIB Trial **eTable 5.** Intravesical BCG Treatment Received by Patients Within the SELENIB Trial **eTable 6.** Intravesical Chemotherapy Received by Patients Within the SELENIB Trial **eTable 7.** Adverse Events by CTCAE Grade **eTable 8.** Adverse Events Occurrence and Incidence During the SELENIB Trial **eTable 9.** Serious Adverse Events Occurrence and Incidence During the SELENIB Trial **eTable 10.** Recurrence-Free Survival Estimates at Yearly Intervals for Each Treatment Comparison **eTable 11.** Recurrence-Free Interval Cox Model Estimates Adjusted for Prognostic Factors **eTable 12.** Recurrence-Free Interval Cox Model Estimates Adjusted for EAU Risk Group **eFigure.** Quality-of-Life Analysis
eReferences

Supplement 3.
Nonauthor Collaborators

Supplement 4.
Data Sharing Statement
