# Supplementary material for: Selenium and Vitamin E for Prevention of Non–Muscle-Invasive Bladder Cancer Recurrence and Progression: A Randomized Clinical Trial
Source: JAMA Netw Open. 2023 Oct 17;6(10):e2337494. doi: 10.1001/jamanetworkopen.2023.37494 (PMC10582794; doi:10.1001/jamanetworkopen.2023.37494)
Supplement: Supplement 1. — Trial Protocol [file jamanetwopen-e2337494-s001.pdf]

BLADDER CANCER PROGNOSIS PROGRAMME

Cancer Research UK Bladder Cancer Group

A Multi Centre Longitudinal Study

Protocol

FINAL VERSION 1.0 November 2005

PLEASE DESTROY ALL PREVIOUS DRAFTS

MREC APPROVAL: 23rd November 2005
START DATE: 1st January 2006

A programme of research

Conducted by: UNIVERSITY OF BIRMINGHAM

Funded by: CANCER RESEARCH UK

CONTACTS

Chief Investigator

Professor KK Cheng
The Public Health Building
Department of Public Health and Epidemiology
The University of Birmingham
Edgbaston
Birmingham B15 2TT
Tel: 0121 414 6757
Email: k.k.cheng@bham.ac.uk

BCPP Study Office

Mrs C Langford – Study Co-ordinator
Room G08, The Public Health Building
Department of Public Health and Epidemiology
The University of Birmingham
Edgbaston
Birmingham B15 2TT
Tel: 0121 414 7867 Fax: 0121 414 3630
Email: c.langford@bham.ac.uk

Other Investigators

Dr LJ Billingham
CRUK Institute for Cancer Studies
The University of Birmingham
Biostatistician

Mr RT Bryan
University Hospital Birmingham NHS
Foundation Trust
Urologist

Dr N Deshmukh
University Hospital Birmingham NHS
Foundation Trust
Pathologist

Dr SA Hussain
CRUK Institute for Cancer Studies
The University of Birmingham
Oncologist

Professor ND James
CRUK Institute for Cancer Studies
The University of Birmingham
Lead Oncologist

Dr PG Murray
CRUK Institute for Cancer Studies
The University of Birmingham
Molecular Biologist

Mr DMA Wallace
University Hospital Birmingham NHS
Foundation Trust
Lead Urologist

Dr MP Zeegers
Department of Public Health and Epidemiology
The University of Birmingham
Genetic Epidemiologist

TABLE OF CONTENTS

SECTION 1: INTRODUCTION AND STUDY SUMMARY ... 7

1.1. PROGRAMME BACKGROUND ... 7

1.2. PROGRAMME OVERVIEW ... 8

SECTION 2: PROJECT A1: LONGITUDINAL COHORT STUDY OF DIETARY, LIFESTYLE AND ENVIRONMENTAL FACTORS ... 10

2.1. BACKGROUND ... 10
2.1.1. Smoking ... 10
2.1.2. Diet ... 11
2.1.3. Total fluid intake ... 12
2.1.4. Alcohol drinking ... 12
2.1.5. Coffee drinking ... 12
2.1.6. Tea drinking ... 13
2.1.7. Artificial sweeteners ... 13
2.1.8. Medication ... 13
2.1.9. Occupational Exposures ... 14
2.1.10. Environmental exposures ... 14
2.1.11. Radiation ... 15
2.1.12. Urine pH ... 15
2.1.13. Menstrual, reproductive, and hormonal factors ... 15
2.1.14. Familial history of bladder cancer ... 15
2.1.15. Hair dyes ... 15

2.2. OBJECTIVE ... 16

2.3. RESEARCH QUESTIONS ... 16
2.3.1. Primary research questions ... 16
2.3.2. Exploratory research questions ... 17

2.4. METHODS ... 17
2.4.1. Study design ... 17
2.4.2. Setting ... 17
2.4.3. Study population ... 18
2.4.4. Recruitment of the cohort ... 18
2.4.5. Inclusion / Exclusion Criteria ... 18
2.4.6. Patient identification, recruitment and consent process ... 19

2.5. DATA COLLECTION ... 19
2.5.1. Baseline data collection ... 19
2.5.2. Postal questionnaire and diary ... 20
2.5.3. Follow-up ... 20
2.5.3.1. Initial follow up ... 20
2.5.3.2. Annual follow up ... 21
2.5.4. Follow-up after recurrence or progression ... 21
2.5.5. Central pathological review ... 21

2.6. STATISTICAL CONSIDERATIONS ... 21
2.6.1. Primary Outcomes ... 21
2.6.2. Main statistical analysis ... 22
2.6.3. Sample size and statistical power ... 22

SECTION 3: PROJECT B: LONGITUDINAL STUDY OF HEALTH RELATED QUALITY OF LIFE 24

3.1. BACKGROUND 24
3.1.1. Impact on health related quality of life (HRQL) 24
3.1.2. Utility-based patient preference for regular surveillance 24

3.2. OBJECTIVES 25

3.3. METHODS 25
3.3.1. General description of quality of life over time 26
3.3.2. Assessing the effect of repeat cystoscopy 26
3.3.3. Assessing the effect of urostomy and self-catheterisation 26
3.3.4. Establishing the effect of recurrence and progression on quality of life 26
3.3.5. Assessing patient preference and utility of hypothetical prognostic models 26

3.4. DATA ANALYSIS 27

SECTION 4: PROJECT C: BLADDER CANCER TISSUE BANK 28

4.1. BACKGROUND 28

4.2. OBJECTIVES 28

4.3. METHODS 29
4.3.1. Identifying patients 29
4.3.2. Specimen identification and labelling 29
4.3.3. Bladder tissue collection and processing 29
4.3.3.1. Snap frozen bladder tissue 29
4.3.3.2. Paraffin-embedded bladder tissue 30
4.3.3.3. Central Pathology Review Procedures 31
4.3.4. Blood and urine collection and processing 31
4.3.5. Transfer of specimens to the BCPP Tissue Bank 33
4.3.6. Retrieval of specimens from the BCPP Tissue Bank 33
4.3.7. Specimen collection standard operating procedures 34
4.3.8. Tissue bank quality assurance 34

SECTION 5: PROJECT D: STUDY OF THE PREDICTIVE EFFECT OF A PANEL OF MOLECULAR MARKERS ON RECURRENCE OR PROGRESSION OF BLADDER CANCER 35

5.1. BACKGROUND 35
5.1.1. The need for a good prognostic model based on molecular markers 35
5.1.2. Limitations of the research to date on the use of molecular markers for prognostication 35

5.2. OBJECTIVES 36

5.3. METHODS 36
5.3.1. Immunohistochemistry (IHC) 36
5.3.2. Tissue Arrays 36
5.3.3. PCR-SSCA 37

5.4. STATISTICAL CONSIDERATIONS 37
5.4.1. Data Analysis 37
5.4.2. Sample size 38

SECTION 6: PRACTICAL ORGANISATION OF THE PROGRAMME 39

6.1. SPONSORSHIP 39

6.2. THE BCPP WORKING GROUP 39

6.3. CENTRE PARTICIPATION 40
6.4. ETHICAL CONSIDERATIONS 40
6.5. PRACTICAL ADMINISTRATION 40
6.5.1. The Protocol 40
6.5.2. Questionnaires 41
6.5.3. Case Report Forms (CRF's) 41
6.5.4. Data Collection 41
6.5.5. Confidentiality of patient data 41
6.5.6. Data collection and security procedures 42
6.5.7. Use of translation / interpreters 42
6.5.8. Monitoring and quality assurance 43
6.5.9. Access to BCPP data 44
6.5.10. Access to Specimens within the BCPP Tumour Bank 44
6.5.11. Publication Policy 44
6.5.12. NCRI Bladder Cancer Studies Group 45

APPENDIX 1: PATIENT INFORMATION SHEETS AND CONSENT FORM 46

APPENDIX 2: BASELINE, POSTAL AND FOLLOW-UP QUESTIONNAIRES 54
Initial questionnaire 55
Postal questionnaire 80
Food, fluid and micturition diary 93
Follow-up questionnaire 122

APPENDIX 3: MOLECULAR MARKERS TO BE EXAMINED 139

APPENDIX 4: SITE PARTICIPATION FORM 142

APPENDIX 5: BCPP PATIENT PATHWAY 143

APPENDIX 6: SCHEDULE OF CRF RETURN 144

APPENDIX 7: TOENAIL COLLECTION PROTOCOL 145

APPENDIX 8: DECLARATION OF HELSINKI 150

APPENDIX 9: REFERENCES 154

List of Figures

Title	Page
**Figure 1:** BCPP programme overview	8
**Figure 2:** Frozen tissue collection and handling process	30
**Figure 3:** Blood and urine collection and handling process	32
**Figure 4:** BCPP patient pathway	143

List of Tables

Title	Page
**Table 1:** Overview of molecular markers associated with bladder cancer recurrence or progression	36

ABREVIATIONS

BCPP	Bladder Cancer Prognosis Programme
CI	Confidence Interval
CIS	Carcinoma in situ
CRCTU	Cancer Research UK Clinical Trials Unit, University of Birmingham
CRF	Case report form
CRUK	Cancer Research UK
EORTC	European Organisation for Research and Treatment of Cancer
GCP	Good clinical practice
GLP	Good laboratory practice
HRQL	Health-related quality of life
IARC	International Agency for Research on Cancer
ICH GCP	International Conference on Harmonisation in Good Clinical Practice
IHC	Immunohistochemistry
ISUP	International Society of Urologic Pathology
MHRA	Medicines and Healthcare Products Regulatory Agency
NCRI	National Cancer Research Institute
NTRAC	National Translational Cancer research Network
OR	Odds Ratio
PI	Principal Investigator (lead investigator at each site)
SOP	Standard operating procedure
TCC	Transitional cell carcinoma
TURBT	Transurethral resection of bladder tumour
US NCI	United States National Cancer Institute
VPN	Virtual private network
WHO	World Health Organization

SECTION 1: INTRODUCTION AND STUDY SUMMARY

1.1. Programme Background

Bladder cancer is the fifth most common cancer in the UK with over 12,000 new cases and 5,000 deaths per year.[1] Worldwide, there are approximately 330,000 new cases and 130,000 deaths per year.[2] In Europe and North America 80-90% of bladder cancers are transitional cell carcinomas (TCCs) of urothelial origin.[3;4]

Approximately 70% of TCCs present as stage Ta, Tis or T1 (non-muscle-invasive or “superficial”). [5-7] Treatment of these conditions relies upon a thorough initial staging resection of the bladder tumour (TURBT), followed by regular cystoscopic surveillance. Optimal additional treatment comprises intravesical chemotherapy (e.g. mitomycin C) within 24 hours of TURBT and/or a course of further mitomycin C or intravesical BCG, which reduce risk of recurrence and progression respectively.[8-11] Despite these treatments the risks of recurrence and progression remain high. It is estimated that, excluding solitary grade 1 pTa tumours, the rates of recurrence and progression are about 50-60% at five years.[12] This means that at any one time in the UK 70,000 – 80,000 patients with TCCs are at risk of recurrence.[12]

In the US, bladder cancer is the fifth most expensive cancer in terms of health care expenditures.[13] It also has the highest cost per patient from diagnosis to death among all cancers in the Medicare system.[14] In the UK individual patient management is more costly for bladder cancer but less is invested in research than for prostate cancer (BJU International 2005; 95: 59-63). The impact of recurrence and progression are thus significant, both for the patients in terms of quality of life and for the NHS in terms of costs. There is therefore an urgent need for effective interventions that reduce the risk of recurrence and progression, and for a prognostic tool that could predict adverse outcomes. These interventions and markers could potentially bring about health gains in a very large number of patients. Apart from the direct benefit to patients, the reduction in frequency of cystoscopic surveillance would bring huge healthcare savings.

1.2. Programme Overview

Objectives

1. To assess the effect of lifestyle factors (such as smoking, dietary habits, fluid intake and environmental exposures) on the recurrence and progression of bladder cancer
2. To study health related quality of life and its association with recurrence and progression of bladder cancer.
3. To establish a bladder cancer tissue bank (that will comprise blood, urine, and bladder tissue).
4. To study the predictive effect of molecular markers on the recurrence and progression of bladder cancer.

Methods

The study will be based on a cohort of patients with newly-detected bladder cancer in all 16 urological centres within the West Midlands, commencing in late 2005 for a period of 5 years.

Figure 1: BCPP programme overview

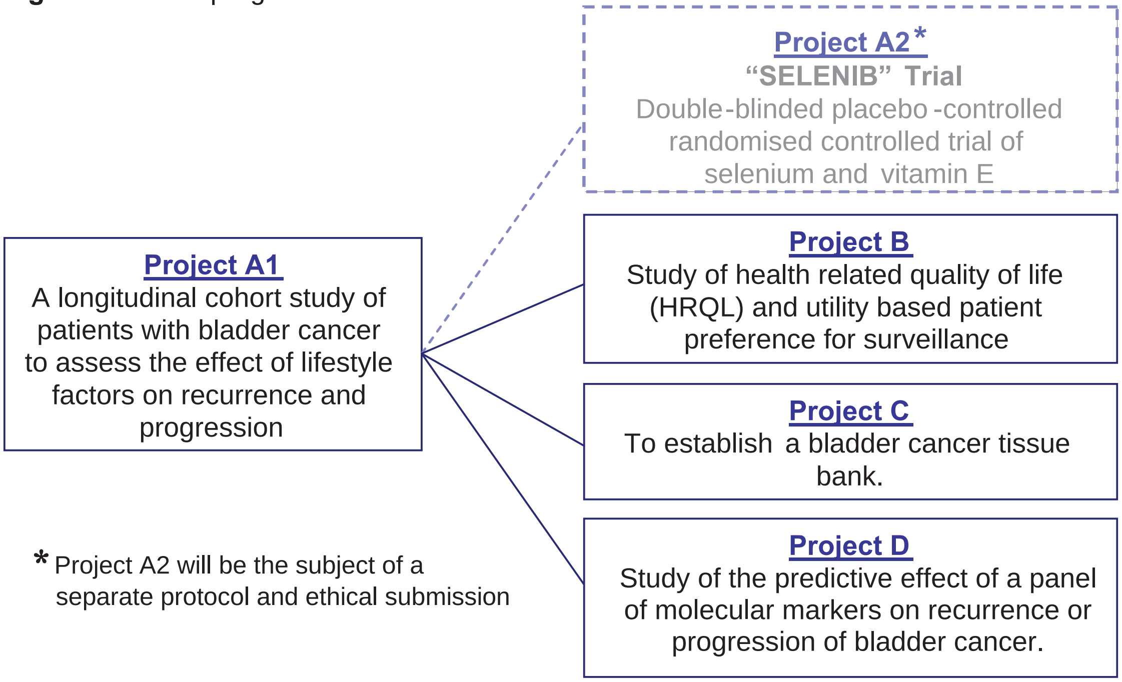

N.B. Both muscle invasive and non-muscle invasive bladder cancer patients will be recruited into the BCPP cohort. However, the analysis of Projects A1, A2 and D will focus mainly on the non-muscle invasive subgroup. See section 2.3.1 for further details.

Primary Endpoints

1. Recurrence of bladder cancer
2. Progression of bladder cancer.

Inclusion Criteria

- Disease characteristics – Bladder lesion with cystoscopic characteristics compatible with urothelial cancer/TCC.
- Able to give informed consent.
- Age – 18 years or above.
- Gender – both.
- Fit for cystoscopy and surgical biopsy/resection

Exclusion criteria

- Previous diagnosis of cancer of the urethra, bladder, uretor or renal pelvis within the 10 years prior to current diagnosis.
- Diagnosis of HIV infection
- Any condition, which, in the opinion of the local investigator, might interfere with the safety of the patient or evaluation of the study objectives.

Data collection

Baseline questionnaire (prior to TURBT)

- Socio-demographic data
- Health related behaviours questionnaire
- Medical history and medications
- Health related quality of life questionnaire (HRQL)
- Social support questionnaire

Self completion questionnaire (prior to first follow-up)

- Food, fluid and micturition diary
- Occupational and residential history
- Family history of cancer

Follow-up questionnaire (At 3m, 1yr, 2yr, 3yr, 4yr and 5yr post surgery)

- Changes to health related behaviours follow-up questionnaire
- Changes to medical history and medications
- Health related quality of life questionnaire (HRQL)
- Utility based patient preference for surveillance questionnaire.

Case report forms (CRF's)

- Clinical treatment data
- Histological data
- Follow-up data
- Event data (recurrence, progression or death)

Biological specimen collection

- Samples of snap frozen & paraffin-embedded bladder tissue, blood, and urine (taken at diagnosis and each subsequent recurrence)
- Toenail clippings for baseline selenium level determination.

SECTION 2: PROJECT A1: LONGITUDINAL COHORT STUDY OF DIETARY, LIFESTYLE AND ENVIRONMENTAL FACTORS

The Effect of Environmental Exposures, Diet, Lifestyle and Other Factors on Recurrence and Progression of Bladder Cancer: A Longitudinal Cohort Study

2.1. Background

The main causes of TCCs in developed countries are cigarette smoking and occupational exposure to carcinogens.[15] Genetic susceptibility may also play an important role, as reflected by the large variation in incidence between ethnic groups and also among populations with similar exposures to smoking and occupational carcinogens.[16] There is also evidence on the protective effects of other exposures, such as the dietary intake of selenium and vitamin E.

On the question of recurrence and progression, there is almost no information on the importance of environmental exposures in determining risk. Unlike malignancies that often present at advanced stages and/or have rapidly progressive courses (e.g. pancreatic, oesophageal, lung), the natural history of non-muscle-invasive TCC provides larger scope for intervention to limit the risk of adverse outcomes. In addition to investments in novel pharmaceutical interventions, modification of environmental exposures by dietary supplementation and lifestyle changes could result in substantial benefits.

2.1.1. Smoking

Evidence from epidemiological studies has shown that cigarette smoking substantially increases the risk of bladder cancer. It has been estimated in a recent meta-analysis of 43 published studies that current cigarette smokers have an approximately threefold higher risk of bladder cancer than non-smokers.[17] The Netherlands Cohort study[18] found that the association of cigarette smoking with bladder cancer risk was largely attributable to duration of smoking. In a large case control study in the West Midlands, Sorahan et al also found that stopping smoking led very quickly to reduction of risk.[19] These results may indicate that the promoting activity of cigarette smoke is of more importance than the initiating activity and consequently that cigarette smoking may also influence the progression of bladder cancer.

Health benefits in stopping smoking are unquestionable and brief advice is already given in most urology centres to newly diagnosed bladder cancer patients who smoke. Such a practice will continue in this project. However, one should bear in mind that cancer diagnosis represents a major life event. Therefore, stronger evidence that stopping smoking would

improve prognosis is likely to be needed before widespread introduction of *intensive* cessation support for smokers newly-diagnosed with bladder cancer.

The following summary of the role of other risk factors is extracted from a recent comprehensive review of bladder cancer epidemiology.[20]

2.1.2. Diet

Fruit and Vegetables

Most observational studies that investigated the consumption of fresh fruits and vegetables have shown a protective effect against development of bladder cancer.[21] A meta-analysis of 10 epidemiological studies reported an increased risk of bladder cancer associated with diets with a low fruit content (RR=1.40, 95%CI: 1.08, 1.83).[22] Regarding vegetable consumption, the same authors provided a meta-OR of 1.16 (95%CI: 1.01, 1.34) associated with diets low in vegetable content, based on 12 studies.

Meat

The effects of fat and meat intake were also summarized in a meta-analysis.[23] Elevated risks were identified for diets with a high fat content (RR=1.37, 95%CI: 1.16, 1.62) but not for diets with a high meat content (RR=1.08, 95%CI: 0.82, 1.42). Whether energy intake mostly accounts for this excess risk has not been elucidated. Only one case-control study has investigated the effect of heterocyclic amines (carcinogens arising from the cooking of meat and fish at high temperatures) and failed to find a relationship with bladder cancer.[24]

Vitamins and minerals

Six prospective cohort studies have investigated the association between serum or toenail selenium and bladder cancer incidence.[25-30] We have previously reviewed the results from five of them[31]. In the largest of these (the Netherlands Cohort Study: 120,852 men and women followed up for up to 7 years, resulting in 431 bladder cancer cases), we showed a significant inverse association between baseline selenium and subsequent bladder cancer risk. The rate ratio for the top vs bottom quintile of toenail selenium levels was 0.67 (95% CI 0.46-0.97), with a statistically significant linear trend over the five quintiles. This association was particularly strong in muscle-invasive tumours, indicating that selenium might be associated with cancer progression. Apart from smoking, selenium was the only important environmental exposure identified to be significantly associated with bladder cancer risk in this large cohort study, thus strengthening the possibility that the relationship is causal on the ground of specificity.[32] Of the other four studies[33-36], three[37-39] reported reduction in risk associated with higher baseline selenium but their powers were limited by a relatively small number of cases (92 cases in

total). The fourth of these studies found a non-significant positive association in women (based on only 9 cases) and a non-significant negative association in men (26 cases) between selenium level and bladder cancer incidence.[40]

A meta-analysis did not find increased risks for diets low in retinol (RR=1.01, 95%CI: 0.83, 1.23) or beta-carotene (RR=1.10, 95%CI: 0.93, 1.30).[41] There is no evidence that dietary or supplement intake of potassium, sodium, calcium, magnesium, phosphorus, iron, vitamin B1, B2, B6 and B12, niacin or folic acid affect UBC risk based on the results of the prospective Health Professionals Follow-Up Study in USA.[42]

2.1.3. Total fluid intake

The urogenous-contact hypothesis (or "field cancerisation" hypothesis) of transitional cell carcinogenesis associates the initiation and progression of bladder cancer with prolonged exposure to urinary carcinogens.[43;44] High consumption of fluids may reduce this exposure by diluting the urine and reducing contact time through increased frequency of urination. Among epidemiological studies, the large Harvard Health Professional Study[45] reported a negative association between high fluid intake and subsequent bladder cancer risk but the finding was not replicated elsewhere.[46;47] Only one study evaluated the association between fluid intake and bladder cancer prognosis and found no relationship. However, power was limited by the relatively small sample size.[48] One of the reasons for these inconsistent findings could be the differential roles of different types of beverage.

2.1.4. Alcohol drinking

Many studies have evaluated the role of alcoholic beverage consumption on bladder cancer risk and have provided inconsistent results. A recent meta-analysis indicated no effect for alcohol consumption on bladder cancer risk.[49] The analysis was based on 16 studies, the summary ORs for alcohol consumption being 1.3 (95%CI: 0.9, 2.0) for men and 1.0 (95%CI: 0.6, 1.7) for women.

2.1.5. Coffee drinking

The role of coffee in bladder cancer is not clear in spite of several epidemiological studies. In 1991, an IARC working group concluded that coffee is possibly carcinogenic to the human urinary bladder, though the possibilities of a bias or an influence of confounding factors (i.e. tobacco) could not be excluded.[50] Zeegers et al, in a meta-analysis, found an adjusted summary ORs for current coffee consumption of 1.26 (95%CI: 1.09, 1.46) for 16 studies in men, 1.08 (95%CI: 0.79, 1.46) for 12 studies in women and 1.18 (95%CI: 1.01, 1.38) for 14 studies with men and women combined.[51] A pooled analysis of 10 case-control studies

conducted in Europe including only non-smokers, found an excess risk for heavy coffee drinkers (OR=1.8, 95%CI: 1.0, 3.3).

2.1.6. Tea drinking

The results of the studies evaluating the effect of tea consumption on bladder cancer are also inconsistent. In 1991, the IARC working group concluded that there was inadequate evidence for the carcinogenicity of tea consumption in humans.[50] A recent meta-analysis has not found any association between tea consumption and bladder cancer.[52] The adjusted summary ORs for current tea consumers compared to non-drinkers was 1.08 (95%CI: 0.94, 1.24) for 7 studies with men, 0.99 (95%CI: 0.81, 1.20) for 6 studies with women and 1.01 (95%CI: 0.92, 1.10) for 7 studies with men and women combined.

2.1.7. Artificial sweeteners

Cyclamate was banned in 1970 from all dietary foods and fruits in the USA by the Food and Drug Administration because of induced cancer in experimental animals.[53] The European Union and the World Health Organization concluded in later research that cyclamate is not a carcinogen and readmitted it to the food market.[54] Besides cyclamate there are other artificial sweeteners on the market in the UK; saccharin, stevioside, thaumatin, aspartame, and acesulfame-k.[55] In 1999, the IARC evaluated the effect of saccharin and its salts on bladder cancer. The working group's conclusion was that these substances were not classifiable as carcinogenic in humans, despite there being sufficient evidence for the carcinogenicity of sodium saccharin in experimental animals.[56]

2.1.8. Medication

Phenacetin

The use of phenacetin-containing analgesics has been associated with an increased risk of renal pelvis TCC's. An association with bladder cancer has been found in most, but not all, published case-control studies.[57-59] In 1987, the IARC included phenacetin in Group 2A (probably carcinogenic to humans) and the analgesic mixtures containing phenacetin in Group 1 (human carcinogens). [60]

Paracetamol

In contrast, acetaminophen (paracetamol), the major metabolite of phenacetin, has generally not been found to increase the risk of bladder cancer [61-63]. Only the case-control study conducted by Steineck et al (1995) found a significant risk increase for TCC among users of paracetamol (OR=1.6; 95%CI: 1.1, 2.3). [64] However, analyses by duration and quantity of paracetamol use did not support this association. In 1999, the IARC concluded that there was

inadequate evidence for both humans and experimental animals as to the carcinogenicity of paracetamol. [65] Subsequent publications have also failed to demonstrate an association with bladder cancer risk. [66-69]

NSAID's

Regarding the use of non-steroidal anti-inflammatory drugs (NSAIDs), the intake of any class of NSAIDs, except for pyrazolone derivatives, was inversely associated with bladder cancer. [70] The protective effect was strongest among regular users of acetic acid compounds (OR=0.54; 95%CI: 0.31, 0.94) and weaker for users of aspirin, other salicylic acids and oxicams. However, other studies did not find any significant effect of NSAIDs on bladder cancer risk. [71-75]

Cyclophosphamide

Cyclophosphamide use has been found to be associated with an increased risk of bladder neoplasms [76-78] and it is also included in the group of human carcinogens. [79]

2.1.9. Occupational Exposures

Exposure to specific chemical carcinogens is another well-established risk factor for bladder cancer. Exposure to aromatic amines is associated with bladder cancer. [80] An excess risk of bladder cancer was also reported for dyers in textile industries, painters, varnishers and hairdressers.[81]

Other agents that have been associated with bladder cancer include polycyclic aromatic hydrocarbons (PAHs), used in aluminum production, coal gasification, coal tars, roofing and carbon black manufacture [82]. An excess risk in bladder cancer was reported among workers exposed to diesel engine exhaust, such as drivers. In a meta-analysis, the summary relative risk among truck-drivers was 1.17 (95%CI: 1.06, 1.29) and among bus drivers, it was 1.33 (95%CI: 1.22, 1.45). [83] High-risk occupations may account for 5-10% of bladder cancer cases in European men. [84]

2.1.10. Environmental exposures

Contaminants in drinking water

Several studies have found a positive association between chlorination by-products in drinking water and bladder cancer. [85-89] Some studies have found increased bladder cancer risks associated with exposure to high-levels of arsenic in drinking water. [90-93] An IARC working group has evaluated and classified arsenic in drinking water as carcinogenic to humans [94]. The data on exposure to nitrate in drinking water is limited and inconsistent.[95;96]

2.1.11. Radiation

Chronic low-dose radiation could affect bladder urothelium through oxidative stress and impairment of DNA repair. Therapeutic pelvic radiation, used for dysfunctional uterine bleeding and ovarian, cervical and prostate cancer is associated with an increase in bladder cancer risk. [97-100] Radioiodine (iodine-131), used for treating hyperthyroidism, was reported as being associated with bladder cancer in some, [101;102] but not all, publications. [103]

2.1.12. Urine pH

Rothman et al found that low urine pH was associated with elevated levels of free urinary benzidine and N-acetylbenzidine and 10-fold higher DNA adduct levels in exfoliated urothelial cells. [104] However, a recent case-control study did not detect an association between urine pH and bladder cancer. [105]

2.1.13. Menstrual, reproductive, and hormonal factors

Hormonal factors have been proposed as one explanation for the excessive incidence of bladder cancer in men. [106] Nevertheless, data on hormonal, menstrual, and reproductive factors and bladder cancer risk are scarce. A case-control study reported a decreased risk for ever-parous women who never-smoked (adjusted OR=0.51; 95%CI: 0.30, 0.88) and no apparent effect among ever smokers. [107] A more recent case-control study has not confirmed this association and has not found significant results for other menstrual and reproductive factors. [108] However, an elevated risk of bladder cancer for ever users of menopause hormone replacement therapy was observed (adjusted OR=3.29; 95%CI: 1.49, 7.25). [109]

2.1.14. Familial history of bladder cancer

Familial clustering of bladder cancer has been described in several case-reports.[110-113] The largest case-control study, including 2,982 bladder cancer patients and 5,782 controls, reported a statistically significant risk of bladder cancer for those with family history of genitourinary cancer (OR=1.45, 95%CI:1.2, 1.8). [114] The risk appeared to be higher among patients under age 45 (OR=2.7, 95%CI: 0.8, 8.9) and among women (OR=1.8, 95%CI: 1.1, 2.7).[115]

2.1.15. Hair dyes

The risk of bladder cancer associated with personal use of hair dyes is uncertain. Two cohort studies and several case-control studies have not observed any effect of permanent hair dye use and bladder cancer incidence or mortality. [116-121] Several other cohort and case-control studies have found an increased risk of bladder cancer among hairdressers and barbers who

are occupationally exposed to hair dyes.[122] Combined, these cohort studies give an estimated relative risk for bladder cancer associated with occupational exposure to hair dyes of 1.4 (183 observed vs 129 expected) based on 81,075 subjects (RR=1.1 for females and RR=1.6 for males).[123] A recent case-control study also reported an elevated risk of bladder cancer for women with frequent and long-term permanent dye use. [124] However, the carcinogenic risk associated with personal use of hair dyes remains unclear.

2.2. Objective

To examine if common and potentially modifiable dietary, lifestyle and environmental exposures affect the risk of recurrence and progression in bladder cancer.

2.3. Research Questions

2.3.1. Primary research questions

The following primary research questions have been selected on the basis of the strength of available evidence. They are also potentially amenable to modification by behavioural and lifestyle changes.

1. Among patients who are cigarette smokers at diagnosis, is stopping smoking associated with a lower risk of recurrence or progression compared to those who continue?
2. Is total fluid intake associated with the risk of recurrence or progression?
3. Is total fruit consumption associated with the risk of recurrence or progression?
4. Is toenail selenium level associated with the risk of recurrence or progression?

N.B. The effects of selenium and Vitamin E dietary supplementation will be investigated by a linked prospective randomised control trial (“SELENIB”). The RCT will be established with a related but separate protocol, which will be the subject of a separate ethical submission. The trial will involve patients with histologically diagnosed TCC drawn from the study cohort and will require an additional informed consent procedure.

Due to the distinct disease characteristics and natural histories of non-muscle-invasive and muscle-invasive bladder cancer, it is imperative to examine these subgroups separately in relation to the above research questions. However a subgroup analysis of muscle invasive bladder cancer would lack power due to the relatively small number of cases. Therefore the focus of this study will be on patient with non-muscle-invasive bladder cancer, whilst collecting valuable information on patients with muscle-invasive disease.

2.3.2. Exploratory research questions

Are other putative risk factors for bladder cancer associated with bladder cancer recurrence or progression? These factors include:

- Smoking (Active and Passive)
- Diet (including vitamins and minerals)
- Types of beverages
- Medications
- Hair Dye
- Urine pH
- Artificial Sweeteners
- Medical history
- Menstrual and reproductive factors
- Family history of cancer
- Residential and occupational histories as proxies for environmental exposures.

2.4. Methods

2.4.1. Study design

Prospective Cohort study.

2.4.2. Setting

All Urology centres in the West Midlands are invited to participate in The BCPP. The following Urology centres are located within the West Midlands:

- Arden Cancer Network
    - George Eliot Hospital NHS Trust
    - South Warwickshire General Hospitals NHS Trust
    - University Hospitals of Coventry and Warwickshire NHS Trust
    - Worcestershire Acute NHS Trust
- Black Country Cancer Network
    - Dudley Group of Hospitals NHS Trust
    - Royal Wolverhampton Hospitals NHS Trust
    - Walsall Hospitals NHS Trusts
- North West Midlands Cancer Network
    - Mid Staffordshire General Hospitals NHS Trust
    - Shrewsbury and Telford Hospitals NHS Trust
    - University Hospital of North Staffordshire NHS Trust
- Derby-Burton Cancer Network
    - Burton Hospitals NHS Trust

- Pan Birmingham Cancer Network
    - Birmingham Heartlands and Solihull NHS Trust
    - Good Hope Hospital NHS Trust
    - Sandwell and West Birmingham NHS Trust
    - University Hospital Birmingham NHS Foundation Trust
- Three Counties Cancer Network
    - Hereford Hospitals NHS Trust

2.4.3. Study population

Post-cystoscopy patients with a bladder abnormality that is suspicious of bladder cancer presenting to participating urology centres in the UK's West Midlands health region.

2.4.4. Recruitment of the cohort

Based on the incidence of bladder cancer from previous years (derived from regional cancer registry data), it has been estimated that approximately 4220 new cases of bladder cancer will be registered in the West Midlands during a 3 year study period, of which around 80% (3400) will meet the inclusion criteria for the study. It is expected that 1600 cases of non-muscle invasive TCC will be included in the primary analysis.

2.4.5. Inclusion / Exclusion Criteria

Inclusion Criteria

- Disease characteristics – Bladder lesion with cystoscopic characteristics compatible with urothelial cancer/TCC.
- Able to give informed consent.
- Age – 18 years or above.
- Gender – both.
- Fit for cystoscopy and surgical biopsy/resection

Exclusion criteria

- Previous diagnosis of cancer of the urethra, bladder, ureter or renal pelvis within the 10 years prior to current diagnosis.
- Diagnosed of HIV infection
- Any condition which, in the opinion of the local investigator, might interfere with the safety of the patient or evaluation of the study objectives.

All patients presenting with suspected urothelial cancer based on cystoscopy will be screened for entry into the cohort, subject to satisfying all other inclusion / exclusion criteria. **Primary**

analyses in Project A1 will however focus on patients with histologically proven urothelial cancer meeting one of the following criteria:

- **Stage Ta (WHO grade 2/3, or grade 1 tumours that are either multifocal or >3cm in size)**
- **Stage T1 (any grade)**
- **Stage Tis**

Solitary grade 1 pTa tumours will not be included in the main analysis due to the very low risk of recurrence and progression amongst this group. [125]

2.4.6. Patient identification, recruitment and consent process

Potentially eligible patients will be identified during haematuria clinics on the basis of abnormal cystoscopic findings suggestive of bladder cancer. The patient's written Informed consent will be obtained after initial cystoscopy and before staging TURBT. Patient information sheets (see APPENDIX 1) will be given to patients immediately after their initial cystoscopy by the research nurse. The patient will be asked to take the information sheet home and to decide whether they wish to participate. On returning to hospital, prior to their TURBT, the patient will be asked by the research nurse to give written informed consent.

2.5. Data collection

Detailed information will be collected about the patients' lifestyle and their exposure to risk factors associated with bladder cancer using semi-structured questionnaires. A baseline questionnaire will be administered at the time of diagnosis. Further questionnaires will be administered at regular follow-up visits to capture information relating to changes in exposure. A postal questionnaire will be used to collect historical information that may require the patient to check records or consult family or friends. Patients will also be asked to keep a 1 week food, fluid and micturition diary.

2.5.1. Baseline data collection

A semi-structured questionnaire (see APPENDIX 2) will be used to collect baseline data. This will be administered by the research nurses prior to TURBT.

The following information will be collected:

- Socio-demographic data – age, sex, ethnicity, marital status, education.
- Environmental exposures - smoking, passive smoking, use of hair dye
- Medical history and use of medications.

- Dietary intake – food type frequency, alcohol, caffeine and total fluid intake, use of vitamins and artificial sweeteners.
- Health related quality of life (general questions for cancer patients)
- Social support

In addition, clinical data (tumour histopathology and treatment data) will be collected on case report forms (CRF's) by the research nurse from the patient's medical records. Biological samples will be collected at baseline: these include samples of tumour tissue, blood, and urine. A sample of toenail will also be obtained for selenium level determination.

2.5.2. Postal questionnaire and diary

Prior to the first routine follow-up, patients will be sent a questionnaire to self complete. This questionnaire will collect data on patients' occupational history, residential history, family history of cancer and a 1-week food, fluid and micturition diary (See APPENDIX 2). Patients will be asked to bring the completed questionnaire and diary back with them to the hospital when they attend for their first follow-up appointment.

2.5.3. Follow-up

Patients will be reassessed at routine follow-up visits. Patients participating in this study will have disease status reassessments at each follow-up until year 5, which will be the final follow-up unless a recurrence or progression occurs. They will also be asked to complete a follow-up questionnaire to capture changes in exposure levels. The follow-up questionnaires will be undertaken at the initial post TURBT reassessment (eg. 3 months) and at each subsequent annual follow-up.

2.5.3.1. Initial follow up

The following re-assessment information will be collected at the first routine follow-up (Approximately 3 months post TURBT);

- Follow-up CRF – disease status, details of recurrence or progression if appropriate.
- Changes in fluid intake
- Changes in smoking habits
- Health related quality of life (general cancer and bladder cancer specific questions)
- Social support
- Utility based patient preference for regular surveillance.

2.5.3.2. Annual follow up

The following re-assessment information will be collected at each annual routine follow-up:

- Follow-up CRF – disease status, details of recurrence or progression if appropriate.
- Dietary intake – Food type frequency, alcohol, caffeine and total fluid intake, use of vitamins and artificial sweeteners.
- Changes in smoking habits and use of hair dye, vitamin supplements and artificial sweeteners.
- Changes to medical history or medications
- Health related quality of life (general cancer and bladder cancer specific questions)
- Social support
- Utility based patient preference for regular surveillance.

2.5.4. Follow-up after recurrence or progression

Additional information will be collected following recurrence. This will include pathological data, treatment details and status surveillance. Tumour tissue, blood serum and urine samples will also be collected at resection. Follow-up questionnaire data will also be collected at recurrence or progression. However the utility based patient preference for regular surveillance section will be omitted.

2.5.5. Central pathological review

In order to obtain standardised pathological data, all diagnostic haematoxylin and eosin stained slides will be reviewed centrally (see 4.3.3.3 for further details). The central review will reduce the bias caused by inter-reporter variability. The data generated by the central pathological review will not be used for any auditing purposes, but purely as a measure of consistency.

2.6. Statistical considerations

2.6.1. Primary Outcomes

Recurrence

Defined as the new occurrence of a bladder cancer at the same or different site as the initial index primary cancer and *excluding* recurrences identified at the first 3-month check cystoscopy.

Progression

Defined as a recurrence with:

- an increase in grade from grade 1/grade 2 to grade 3, or

- an increase in TNM stage, or
- the new occurrence of *carcinoma in situ* (CIS) in a bladder previously free from such lesions, or
- the new occurrence of multiple urothelial tumours following resection of a solitary urothelial tumour, or
- the need for a cystectomy because of refractory disease.

2.6.2. Main statistical analysis

The main analysis for project A1 will focus on patients with non-muscle-invasive bladder cancer.

Incidence rate ratios and corresponding 95% confidence intervals for recurrence and progression comparing high versus low levels of exposure for each of the exploratory variables (see 2.3.2) will be estimated by using age-adjusted and multivariable Cox proportional hazards model [126] processed with the Stata statistical software package. [127] The multivariable model will consist of covariates selected by hierarchical top-down evaluation of potential confounders but will include at least age, sex and cigarette smoking. The proportional hazards assumption will be tested using the scaled Schoenfeld residuals. [128]

Tests for dose-response trends in risk of recurrence or progression will be assessed by fitting ordinal exposure level variables as continuous terms. Statistical tests for interaction will be based on Wald statistics.[129] Two-sided p-values will be used throughout the project. A significance level of 0.05 will be used in all analyses.

2.6.3. Sample size and statistical power

<u>Stopping smoking</u>

Out of the cohort of 1,600 patients with non muscle invasive bladder cancer, we conservatively estimate that there would be about 720 current smokers at diagnosis available for this analysis. There is no reliable data on the proportion of patients who would stop smoking after a bladder cancer diagnosis but using information from studies on patients after myocardial infarction as a guide[130], we assume that 30% of smokers would have become non-smokers by the first anniversary. We further assume five-year recurrence and progression rates among those who continue to smoke to be 45% and 23% respectively, and 5% loss to follow up. With a sample of 720, we would be able to detect rate ratios of ≤ 0.66 for recurrence and ≤ 0.54 for progression (80% power, 2-sided $\alpha = 0.05$), comparing those who stop to those who continue. The power would be higher if there are more smokers in the cohort at baseline.

Fluid and fruit consumption and toenail selenium

Power calculation is based on the non-muscle invasive cohort. For comparison between two equal-sized groups, we again assume five-year recurrence and progression rates in those with below median consumptions to be 45% and 23% respectively, and 5% loss to follow up. With 1,600 non-muscle invasive patients, we would be able to detect rate ratios of ≤ 0.78 for recurrence and ≤ 0.70 for progression (above vs below median; 80% power, 2-sided $\alpha = 0.05$). Powers would be considerably higher if these exposures were analysed as continuous variables.

SECTION 3: PROJECT B: LONGITUDINAL STUDY OF HEALTH RELATED QUALITY OF LIFE

A longitudinal study of health related quality of life and utility-based patient preference for regular surveillance

3.1. Background

3.1.1. Impact on health related quality of life (HRQL)

As a chronic condition, bladder cancer poses a large burden on patients and health services (see section 1.1). It is therefore surprising that few studies examining the short- and long-term impacts on patients' HRQL have been reported. Botteman et al recently published a review of the literature and identified only three relevant papers. [131] Two of these described the effect of intravesical BCG in 85 Austrian and 30 German patients respectively.[132;133] The main conclusion from these two studies is that despite unpleasant symptoms (mainly urinary) during treatment, severe impacts on HRQL were uncommon. In the third paper, Shover focused on sexual function and cited clinical anecdotal evidence that repeated cystoscopies and intravesical treatment may have adverse psychological effects that led to sexual dysfunction. [134]

We conclude that previous evidence is very limited, especially on the psychological effects of cystoscopic surveillance, and recurrences. This study therefore provides a unique opportunity to investigate these factors longitudinally in a large sample. The results will also be useful for future evaluations of new technologies in surveillance and therapy.

3.1.2. Utility-based patient preference for regular surveillance

Cystoscopic surveillance remains the method of choice to detect recurrences in patients with bladder tumours. Although the negative effect of each individual cystoscopy may not be very large, the overall impact on HRQL may be significant since many patients would have to undergo these tests regularly for the rest of their lives. However, many patients will not have any recurrence after the initial diagnosis. For these patients, accurate prediction using a good prognostic model could spare them unnecessary physical discomfort and psychological stress.

One of the key aims of this programme (specifically Project D) is to develop a prognostic model based on histopathological and molecular markers. We therefore propose to assess patient preference with a utility-based technique, comparing the current regime of surveillance with an alternative follow-up regimen, based on a hypothetical prognostic tool (that is unlikely

to be 100% accurate). Together with data on the effects of repeat cystoscopy, recurrence and progression, the results will be very important if a good prognostic model becomes available which may suggest the possibility of less intensive surveillance in those deemed to be at low risk. In this context we have identified a study that examined patient opinion of urinary tests compared to flexible cystoscopy in bladder TCC follow-up. It found that 89% of patients preferred cystoscopy if the sensitivity of the urinary test was lower than 90%, and 68% of those sampled expected a sensitivity of over 99% before they would opt out of cystoscopic surveillance. [135] However, this very interesting study had its limitations: it was cross-sectional and the sample size was small (n=102, with only 15 women). We therefore propose to examine the preference of patients longitudinally in our cohort.

3.2. Objectives

- To study the effects of recurrence and progression on HRQL
- To study the effects of repeat cystoscopy on HRQL
- To study the patients' assessments of a hypothetical prognostic model and how this affects their preference for the mode of surveillance.

N.B. The second and third objectives are mainly for patients with non-muscle-invasive tumours as most patients with muscle invasive disease will undergo cystectomy.

3.3. Methods

We will use the quality-of-life questionnaires developed by the European Organisation for Research and Treatment for Cancer. The EORTC QLQ-BLS24 with 24 questions specific to non-muscle-invasive bladder cancer and the EORTC QLQ-BLM30 with 30 questions specific to muscle-invasive bladder cancer will be combined and used in conjunction with the general cancer questionnaire QLQ-C30. [136] The bladder specific questionnaires are in the advanced development phase and we will contribute data that can be used for the evaluation of its psychometric properties (Karen West, personal communication).

Assessments using the QLQ-C30 will be made at baseline in the entire cohort of 3,400 patients. Follow-up assessments using the QLQ-C30, QLQ-BLS24 and QLQ-BLM30 will be made at first routine follow-up and annually until the end of study. We have considered the option of limiting the assessment to a sub-sample within the cohort. However, at baseline, one cannot predict which patients will suffer recurrence(s) or progression. We have therefore decided to survey all patients in the cohort to ensure that we have baseline information with

annual review on all patients. The HRQL questions feature in section 8 of the baseline questionnaire and section 8 of the follow-up questionnaire (APPENDIX 2).

3.3.1. General description of quality of life over time

The assessments made at various time points during patient follow-up will provide a general description of the quality of life of patients at diagnosis and during the course of the disease. The questionnaires will provide information on physical, emotional, social, cognitive and role functioning as well as a measure of global health status. Measures of general symptoms will also be available, together with measures of the impact of the disease on urinary, sexual, and bowel symptoms.

3.3.2. Assessing the effect of repeat cystoscopy

The EORTC QLQ-BLS24 has specific questions that measure the impact of repeated cystoscopy and intravesical therapy on patients. This will allow an accurate description of changes over time.

3.3.3. Assessing the effect of urostomy and self-catheterisation

Most patients diagnosed with muscle-invasive bladder cancer will undergo a cystectomy and will require a urostomy. The EORTC QLQ-BLM30 has 6 specific questions about the impact of urostomy and one question about self-catheterisation. These questions will allow for measurement of the impact of urostomy and self-catheterisation on patients quality of life over time.

3.3.4. Establishing the effect of recurrence and progression on quality of life

In addition to annual assessments of HRQL, assessments will also be made after recurrence or progression. This will enable the effect of recurrence and progression on HRQL to be established. It will also allow us to ascertain the benefits of selenium and vitamin E on HRQL in “SELENIB”.

3.3.5. Assessing patient preference and utility of hypothetical prognostic models

Our method has been adopted from the Standard Gamble technique.[137] We will question all patients in follow-up sessions with a choice using an additional question (see Section 6 of follow-up questionnaire, APPENDIX 2). The options are to continue repeated invasive cystoscopy or to take a gamble and choose a less invasive prognostic test with two possible outcomes: a true negative prediction with probability p or a false negative prediction with the probability 1-p. The probability of outcomes will be varied until the patient is indifferent about the two alternatives. A measure of the utility of the new prognostic test is the lowest p. This

assessment will be taken at the first routine follow-up and then repeated annually. No assessment of this measure will be undertaken at recurrence or progression.

3.4. Data Analysis

The responses from the EORTC questionnaires will be scored according to specified guidelines.[138] The analysis of scores at each time point and in terms of change over time will generally be descriptive. Hypothesis testing will be used for the global health status measure, specifically in terms of changes in score from before and after a recurrence or progression. The utilities of hypothetical prognostic tests will be described. We will investigate whether the perceived utility differs according to sex, duration of disease, recurrence status, and the number of previous cystoscopies.

SECTION 4: PROJECT C: BLADDER CANCER TISSUE BANK

To establish a bladder cancer tissue bank to study the predictive effect of molecular and chromosomal factors on the recurrence and progression of bladder TCC

4.1. Background

Establishing a bladder cancer tissue bank

This study will include a large cohort of patients with newly diagnosed bladder cancer. As initial tissue collection will take place prior to definitive histology, tumour tissue, blood and urine will be collected from all patients, regardless of stage. Together with tissues from patients with non-muscle-invasive tumours, we will also collect samples from patients with muscle-invasive tumours and solitary grade 1 pTa tumours. The samples will be stored in a central repository based at the University of Birmingham. Together with detailed linked information on environmental exposures and clinical outcomes, this tissue bank will form a unique resource in the UK.

4.2. Objectives

- To establish a bladder cancer tissue bank to prospectively store snap frozen and paraffin-embedded bladder tumour tissue for future research into genetic, epigenetic and genomic profiles of bladder cancer. Banked tissue samples will also be utilised in a preliminary study of a panel of molecular markers will be undertaken as part of this protocol (See Project D, Section 5:)
- To prospectively store blood specimens for future research into DNA factors and serum markers.
- To prospectively store urine specimens for future research into the identification and testing of markers that may be used in screening, diagnosis and surveillance of bladder cancer.

These have been highlighted in a recent review of research priorities by the US NCI. [139]

All future research projects which utilise the specimens stored in the BCPP tissue bank will be the subject of separate protocols and ethical submissions.

4.3. Methods

4.3.1. Identifying patients

Informed consent for tissue collection and storage for research and teaching purposes will be sought from all patients recruited into the observational cohort study (Project A1). Details of patient identification and recruitment procedure can be found in section 2.4.6.

4.3.2. Specimen identification and labelling

Specimen tubes/containers will be pre-labelled with a unique specimen number, hospital number, date of birth and study acronym. The specimen number will be used for all bladder tissue, blood and urine specimens collected at the time of the initial diagnosis. The specimen number will include a suffix to identify each individual specimen tube/container. Separate specimen numbers will be allocated for each subsequent recurrence. A Specimen Number Master List matching the patients study number to the specimen number will be maintained in the BCPP Study Office. All processing of samples will be managed via the specimen number in order to ensure that analyses of materials are performed blind. Only designated staff within the BCPP Working Group will be able to access the Master List and thus “back-trace” specimen numbers to patient details.

A tissue collection proforma will be completed in theatre to record details of all samples removed. This proforma will be signed and verified by the responsible surgeon.

4.3.3. Bladder tissue collection and processing

4.3.3.1. Snap frozen bladder tissue

The research nurses will be responsible for tissue collection in the operating theatre. All research nurses will have received training on the handling, storage and retrieval of human biological materials including health and safety measures. They will also be required, where applicable, to use appropriate protective clothing and equipment.

Tissue samples for storage will be selected by the operating surgeon, taking into consideration the priority for clinical samples for histopathological diagnosis. They will be separated from the routine pathology samples and placed directly into cryovial storage tubes and snap-frozen in liquid nitrogen for transfer for temporary storage in a dedicated -80°C freezer in the local hospital.

Samples will be periodically transferred from the local hospital to the central laboratory located within the CRCTU where they will be stored indefinitely in dedicated freezers operating at

-80°C or liquid nitrogen storage tanks at a temperature of -150°C. Prior to transfer, the responsible pathologist will be required the sign the release of the specimens. The minimum period that tissue specimens will be kept in the local hospital prior to transfer will be two weeks. This will enable efficient retrieval of specimens should they be required for clinical purposes.

Figure 2: Frozen tissue collection and handling process

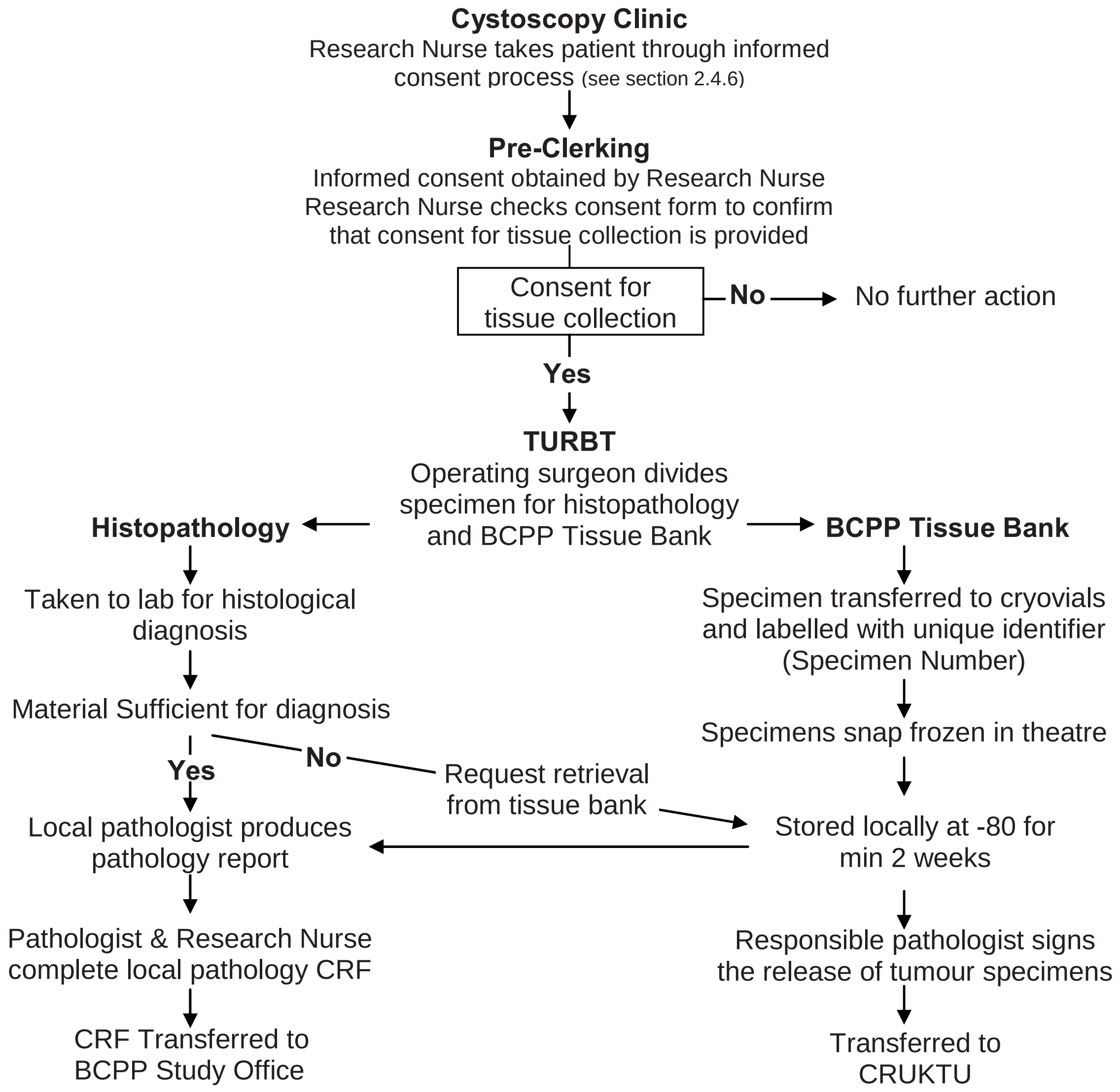

4.3.3.2. Paraffin-embedded bladder tissue

Tissue specimens for routine pathological examination are fixed in formalin in the operating theatre and subsequently transferred to the pathology department. The formalin-fixed tissue is processed and one or more paraffin tissue blocks created. These paraffin blocks are then used to provide tissue sections for diagnostic purposes. For the purposes of the BCPP Project

D (SECTION 5:) formalin-fixed paraffin-embedded tissues will be used to generate tissue arrays, which will then be used for immunohistochemistry to stain for a panel of molecular markers. The process of creating tissue arrays involves taking “Tru-Cut” specimens from a paraffin block and following this the paraffin block is unsuitable for subsequently providing sections for slide mounting. Patients for whom only 1 tissue block exists thus may not be suitable for the collection of tissue for array production. It may nonetheless be possible to take additional unstained slide sections to study expression of individual marker proteins.

To take into account this part of the programme, local pathology departments will be requested to create an extra paraffin block (or blocks), provided that there is enough tissue available. This block will be for the use of the programme and subsequently will be stored centrally. Slides from this block will initially be examined under the microscope by the local pathologist to ensure that this tissue is representative of the tumour sample as a whole. If not enough tissue exists in the original specimen to create an extra paraffin block local pathology departments will be requested to create 10 slide-mounted sections for subsequent central transfer and use within the programme.

4.3.3.3. Central Pathology Review Procedures

Following routine local pathological examination and clinico-pathological staging slides will be transferred for central pathology review. The review will be undertaken by the BCPP pathologists at the Pathology Laboratories of University Hospital Birmingham NHS Foundation Trust. The BCPP pathologists will be blind to the clinical details and local diagnosis. The review diagnosis will provide a stage and grade classifications based on the WHO and WHO/ISUP classification criteria.

The intention of the review is not to alter the clinical diagnosis and management of the patient but to ensure consistency of classification.

Following central pathology review slides will be returned to the local pathology department. Throughout the pathology review process local units will be able to request the immediate return of the slides if required.

4.3.4. Blood and urine collection and processing

At study entry, blood from all patients will be collected in two 5-ml ETDA tubes and one plain 10-ml tube by the research nurse. The EDTA treated sample will be centrifuged at 3000g for 20 min (room temperature) and the plasma removed using a sterile Pasteur pipette and placed into 6 separate appropriately labelled tubes for storage at -80°C. The sample in the

plain tube will be left to clot, at room temperature for no less than 90 min and no more than 150 min. After this time the clotted sample will be centrifuged for 20 min at 3000g (room temperature) and the serum collected using a sterile Pasteur pipette and placed into a labelled fresh centrifuge tube. The serum samples are then re-centrifuged for 10 min at 3000g after which the serum is collected (fresh pipette) and placed into 6 appropriately labelled tubes for storage at -80°C. Subsequent transfer to the tissue repository at the CRCTU will be as for the tissue samples. White blood cells will be separated by standard methodology (e.g. “Lymphoprep”) and frozen and stored at -80°C for subsequent DNA extraction either locally or at the CRCTU (giving a non-tumour DNA control for each patient). Full training will be provided to the research nurses in carrying out the relevant procedures. Subsequent serum samples at recurrence will be collected and processed in the same manner. There will be no further collection of white blood cells after baseline other than to replace missing or technically inadequate samples.

Figure 3: Blood and urine collection and handling process

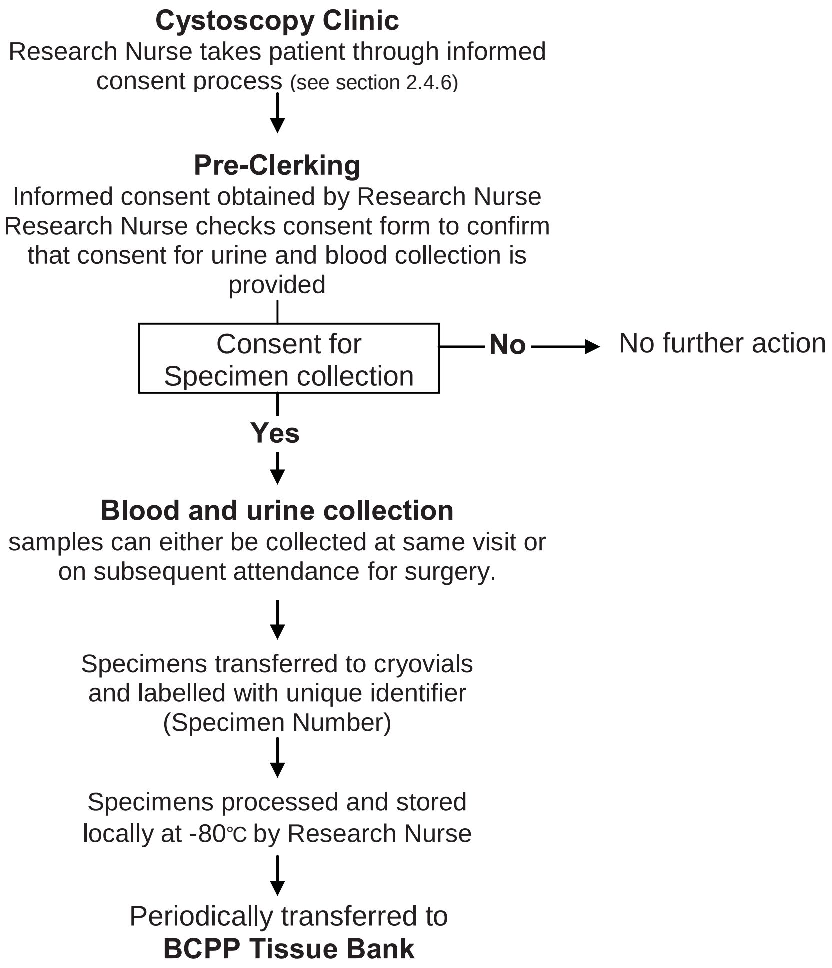

Urine samples will be collected for each patient by the research nurse during routine pre-clerking. The urine will be collected using a urine cup with an integrated sampling device and transferred into an 8ml 'Vacutainer' urine preservative tube. The urine will be centrifuged to separate residues such as cellular debris or urate crystals. The supernatant will be aliquoted into pre-labelled cryovials and frozen at -80°C. Urine samples will be transferred to the central tissue bank as for blood and tissue samples. The urine residue will be separately resuspended in distilled water, frozen and stored as for the supernatant, and stored as for the blood and tissue samples. Urine volumes and concentrations will be normalised by measurement of urine protein and creatinine concentration and comparison with the serum creatinine taken concurrently. Urine pH will also be measured at the time of collection. Evidence of a urinary tract infection will be documented on the CRF to be included in subsequent analysis.

4.3.5. Transfer of specimens to the BCPP Tissue Bank

All specimens will be stored locally until such a time that the responsible pathologist is satisfied that clinico-pathological requirements have been fulfilled and that the specimens can be dispatched for central storage. It is anticipated that this will be a minimum period of 3 months post surgery. Periodically the responsible pathologist will be asked to give signed authorisation for the release of a batch of specimens. A detailed log of all transferred specimens will be provided to the authorising pathologist. The transfer of specimens from local sites to the central tissue bank will be coordinated by the research nurse at the individual urology unit and by the central tissue bank technician.

The specimens for transfer to the BCPP Tissue Bank will comprise:

- Paraffin tissue block (for creating tissue arrays)
- Snap-frozen bladder tumour samples
- Blood samples
- Urine samples.

In order to integrate the central pathological review process, haematoxylin and eosin slides will be requested for transfer at the same time as specimens required for the tissue bank.

4.3.6. Retrieval of specimens from the BCPP Tissue Bank

The location of specimens will be tracked by a computerised system. Should any specimen be required to be returned to the local site for further clinico-pathological staging or due to patient withdrawal of consent, the relevant sample will be speedily retrieved from the bank and returned to the local pathologist.

4.3.7. Specimen collection standard operating procedures

The process of collection, handling, routing and storage of specimens will be controlled by one common set of standard operating procedures (SOP's). The SOP's will ensure full compliance with relevant legislation including the European Union Trials Directive and associated codes of conduct for Good Clinical Practice (GCP) and Good Laboratory Practice (GLP).

The Institute for Cancer Studies, University of Birmingham, has in place a Quality Assurance (QA) system utilising Q-Pulse software for document management. This system mirrors the BSI_ISO9000.2000 accredited QA system already in place in the Cancer Centre at the Queen Elizabeth Hospital for which one of the lead investigators (NJ) is the responsible lead clinician. All SOPs, documentation and procedures will be managed via the Q-pulse system, ensuring consistency with Institute practice and allowing effective sharing of best practice between groups. The Institute has recently had inspections from the MHRA, NTRAC and CRUK, all of which have examined aspects of the QA system and all of which have been satisfied with the procedures in place.

4.3.8. Tissue bank quality assurance

Ensuring the quality of the specimens collected will be an essential part of the BCPP. Tissue bank quality assurance will be a continuous process throughout the life of the programme to ensure that none of the collected tissues will be wasted. Within the first year of the programme a separate study will be instituted to prospectively evaluate the BCPP tissue collection SOP's. This quality assurance study is the subject of a separate research grant application. The objective of this study will be to identify baseline or standard reference profiles of molecular-genetic expression that will subsequently be used to evaluate the quality of the collected tissues. It is anticipated that the evaluation of tissue quality will take place on a six-monthly basis, and will involve the evaluation of at least 10% of all the tissues collected during the life of the programme.

SECTION 5: PROJECT D: STUDY OF THE PREDICTIVE EFFECT OF A PANEL OF MOLECULAR MARKERS ON RECURRENCE OR PROGRESSION OF BLADDER CANCER

5.1. Background

5.1.1. The need for a good prognostic model based on molecular markers

In current practice, the majority of patients with non-muscle-invasive tumours are subject to frequent follow up by cystoscopy, many for the rest of their lives. A prognostic tool that can accurately identify those who are very unlikely to suffer recurrence or progression could spare patients unnecessary invasive procedures and psychological burdens. There could also be enormous cost savings for health services.

Currently, histopathological factors only provide very crude prognostication. In this regard, molecular markers could represent a potential basis for refinement. Such markers may also help to identify patients at high risk of relapse who may benefit from adjuvant therapy. Furthermore, these molecules may provide the targets for novel therapies.

5.1.2. Limitations of the research to date on the use of molecular markers for prognostication

Research in this area has not yet resulted in assisting therapeutic decisions or prognostication. [140] We believe there are at least two main reasons behind this:

<u>Methodological</u>

One important principle of research requires that hypotheses be stated in advance. Unfortunately, it is rare to find clearly stated hypotheses in prognostic factor studies in bladder cancer. As a result, in the literature there are numerous reports on the effect of single markers on which no *a priori* hypotheses were stated when the tissues were collected. Such studies can only be regarded as exploratory and any positive results would need to be confirmed. Previous work is also limited methodologically by small sample sizes, inadequate validation, inadequate clinical follow up, use of different cut-off values, and failure to adjust for known predicting factors.[141-143] This sad state of affairs led Schmitz-Drager et al to provocatively label the field as the “playground of urology scientists”, using p53 immunohistochemistry as a specific example. [144]

<u>The complexity of tumour development</u>

Carcinogenesis involves multiple genetic, epigenetic and cellular changes and so reliance on single markers would almost certainly fail to result in large predictive power.[145;146]

Identifying suitable molecular markers

Our group recently conducted a systematic review of molecular markers in bladder cancer. We have identified those markers for which there has been the strongest or most consistent evidence suggesting that they may be useful in predicting recurrence and progression.[12] Some well-studied markers have been omitted from our list as we felt that the evidence for their role was inconclusive. A detailed description of our panel of 7 markers can be found in APPENDIX 3. A summary of these markers is presented in table 1.

Table 1: *Over*view of molecular markers associated with bladder cancer recurrence or progression

Markers	Hypothesised predominant effect(s)	Number of patients in largest study to date	Reference for method to be used
FGFR3	Recurrence and progression	112	119
EGFR	Progression	101	156
pRB	Progression	363	157
p53	Recurrence and progression	164	157
Ki-67	Recurrence and progression	319	157
VEGF	Recurrence and progression	185	158
CK20	Recurrence	58	159

5.2. Objectives

- To evaluate the prognostic role of a panel of markers chosen *a priori* in a large prospective study using prospectively collected tissues, which will be analysed by standardised and validated methods

5.3. Methods

5.3.1. Immunohistochemistry (IHC)

IHC on tissue arrays will be used to detect the expression of these markers (except FGFR3). The rationale for this technique is twofold: IHC is a technique that is used in NHS histopathology laboratories nationwide and is thus highly clinically applicable; tissue arrays facilitate the handling of large numbers of tumour samples, thus enabling large-scale screening of these molecular markers.

5.3.2. Tissue Arrays

This new technique allows a large number of samples from different patients to be presented on the same slide, thus enabling the large scale screening of samples by IHC. Representative

areas of tumour material from paraffin wax blocks (up to a maximum of four per tumour) are selected for subsequent incorporation into tissue arrays. These are constructed by acquiring cylindrical ("Tru-Cut") biopsies from individual paraffin-embedded tumour tissues into a tissue array block. A subsequent single immunostaining reaction provides information on all of the specimens on the slide. We plan to use a modified method of array preparation to include fewer (50) but larger (5mm diameter) samples per array to reduce the possibility of bias from small sample size.

Patient samples will be identified from pathology records and the archive haematoxylin and eosin (H&E) slide examined for a suitable region of interest. A "Tru-Cut" core corresponding to a representative region will be taken from the tissue blocks and mounted in normal liver, which is used as the support medium on the tissue array slide. 5mm "Tru-Cut" needle cores will be taken from each tumour block to construct tissue arrays, each comprising 50 different tumours. The array block is subsequently sectioned and stained. Staining will then be assessed and reported by two pathologists, assessing the sections independently. To allow for tumour heterogeneity, antibody optimisation and experimental errors, a total of four cores from each block will be taken to create four different sets of tissue arrays from this group of patients. This technique greatly reduces the work involved in screening large numbers of samples and facilitates inter-comparison between different samples by eliminating variations due to staining techniques.

5.3.3. PCR-SSCA

For FGFR3 mutations, DNA will be extracted from paraffin-embedded tissue followed by PCR-SSCA (single-strand conformation analysis) to screen for mutations in exons 7, 10, 15, and 19 of the FGFR3 gene. [147]

5.4. Statistical considerations

5.4.1. Data Analysis

The statistical investigation of specific prognostic markers and the development of a prognostic model will take full account of current thinking regarding the development of reliable prognostic models.[148;149] We will model continuous markers using fractional polynomials. We will explore both parametric and non-parametric models. The independent and joint effects of markers will be examined in a multivariate manner, adjusting also for environmental exposures and known (mainly histopathological) prognostic factors. In patients participating in the SELENIB trial (Project A2), selenium and α-tocopherol status would also

be taken into account in data analysis. This will be performed by independent statisticians so as not to break the blinding.

We will make use of resampling methods (bootstrapping) to investigate the stability of the derived model.[150] We would use the data set to attempt to validate pre-existing models for prognosis in bladder cancer derived from an individual patient data meta-analysis of randomised trials.[151;152] Although high quality, these data would not be useful for reverse validation because of absence of information on all the relevant variables.

5.4.2. Sample size

With 3-year recruitment, median follow up of 36 months (range 18-54 months) and 5% loss to follow up, a sample size of 1,600 non-muscle invasive bladder cancer patients would give us 80% power (2-sided $\alpha = 0.01$, allowing for multiple comparisons) to detect the following effects associated with the markers:

Prevalence of markers (%)	**Rate ratio detectable**	
	Recurrence*	Progression#
10	≥ 1.77	≥ 2.12
50	≥ 1.47	≥ 1.68
90	≥ 1.92	≥ 2.39

*assuming 25% in reference group
assuming 13% in reference group

We will therefore have very good power to detect associations that are likely to be of significant clinical utility. Our sample size is also more than four times larger than the largest individual studies on single markers to date. This implies that almost all previous studies have been underpowered, with substantial risks of false positive findings given the multiple comparisons made in the absence of *a priori* hypotheses.

SECTION 6: PRACTICAL ORGANISATION OF THE PROGRAMME

6.1. Sponsorship

The University of Birmingham undertakes the role of Sponsor for the Bladder Cancer Prognosis Programme. The sponsor delegates all aspects of management and co-ordination of the study to the BCPP Working Group.

6.2. The BCPP Working Group

This study is funded by Cancer Research UK and conducted by The Department of Public Health and Epidemiology and the CRUK Institute for Cancer Studies, University of Birmingham (CRCTU). The BCPP Working Group forms part of the Cancer Research UK Bladder Cancer Group.

The programme will be centrally co-ordinated and managed by the BCPP Working Group, which is a multidisciplinary collaborative group comprising clinical and academic members of The University of Birmingham and University Hospitals Birmingham NHS Trust. The group, led by the Chief Investigator, comprises urologists, oncologists, pathologists, molecular biologists, epidemiologists and statisticians (a full list of BCPP Working Group members can be found on page 2).

The team is based within the BCPP Study Office within the Department of Public Health and Epidemiology at the University of Birmingham. Tissue storage and most of the laboratory and statistical work will be carried out in the nearby CRUK Institute for Cancer Studies. The BCPP Working Group will be responsible for the application for ethical approval, setting up participating centres and continued liaison with and support for the centres. The team will be responsible co-ordinating data and tissue sample collection; tissue, blood and urine banking; data analysis; publication and reporting to ethical and regulatory authorities. The programme will be managed day-to-day by the BCPP Study Co-ordinator under the leadership of the Chief Investigator. The Study Co-ordinator is responsible for the management and supervision of the research nurses in collaboration with clinical managers located within the centres.

The BCPP Study Co-ordinator will establish and oversee the data capture process and will also monitor the data integrity and completeness. The Study Co-ordinator will provide regular reports on performance and data quality to the BCPP Working Group.

6.3. Centre Participation

To participate in the research programme each institution should nominate one responsible urologist who will undertake the role of Principal Investigator for the study at their institution. A signed Site Participation Form (See APPENDIX 4), is required for each centre. The form must be returned to the BCPP Study Office before the first patient is entered into the study by that centre. The BCPP Working Group will undertake a process of site appraisal, investigator consultation and site set-up prior to the launch of the programme at each institution.

6.4. Ethical Considerations

The Chief Investigator will be responsible for ensuring that multi-centre ethical approval is obtained.

The Principal Investigator at each centre will be responsible for ensuring that this study is conducted in agreement with the Declaration of Helsinki (APPENDIX 8) and that local site specific assessment and local R&D approval are obtained. The Study will be conducted in accordance with the principals of the ICH Guidelines on Good Clinical Practice.

Patients will be required to give written informed consent prior to entry into the study (full consent process detailed in section 2.4.6). Information sheets (APPENDIX 1), prepared in accordance with COREC guidelines, will be given to patients at least 24 hours before written consent is requested. Patients will be advised of their right to withdraw from the study. Upon withdrawal, unless the patient gives explicit written permission for the data and tissue to be retained, all study materials relating to the patient will be destroyed. The study number will not be re-allocated.

6.5. Practical Administration

6.5.1. The Protocol

One common protocol will be used at all participating centres. The finalised master protocol will be held at the BCPP Study office. Any amendment to the protocol will be co-ordinated by the BCPP Working Group. Amendments will be submitted to the appropriate regulatory and ethical authorities for approval. Upon approval, amendments will be circulated to all centres incorporated in a fully re-printed copy of the protocol. A full audit trail of such amendments will be maintained and a log of amendments will be included at the front of the newly printed protocols. A process of document control will be implemented to provide assurance that all copies of the protocol that are in circulation are current version. An individual in each centre

will be identified as the document controller for their institution. A shortened summary protocol will also be created for use in clinical areas, containing the parts of this protocol most pertinent to the clinical management of patients.

6.5.2. Questionnaires

One common set of questionnaires will be used at all participating centres (see APPENDIX 2). The questionnaires have been piloted initially within the BCPP Working Group and lay-members of the public. Following ethical approval, the questionnaire will be further tested amongst bladder cancer patients recruited into the study at the first recruiting centre prior to the programme being rolled out to other sites. As a measure of quality assurance, the patient will be asked to verify that the data is correct by signing and dating each questionnaire.

6.5.3. Case Report Forms (CRF's)

One common set of forms will be used at all participating centres. CRF's will be used to capture clinical data directly from source documents (see APPENDIX 6). Source data verification will be undertaken on a random sample of patients. All data will be subject to systematic data checking. Any queries detected by the BCPP Study Office will be fed back to the centres for resolution.

6.5.4. Data Collection

The database for the study will be held at the BCPP Study Office located within the University of Birmingham. Paper copies of all questionnaires, CRF's and supplementary clinical documentation will be stored both locally in the centre and in the BCPP Study Office.

6.5.5. Confidentiality of patient data

All data collected as part of the BCPP will be handled according to the Data Protection Act. The use of names as patient identifiers on paper forms and the study database will be limited. Where possible, an abbreviated patient identifier will be used in place of the patient name. Within the BCPP Study Office and at each participating site, patient identifiable documentation will be held in locked cabinets within an office. The Office itself will be accessible only to authorised personnel. Any electronic correspondence containing patient data will be password protected and encrypted. All study personnel will be required to sign a confidentiality undertaking.

No personally identifiable information will be released from the BCPP Study Office. Limited clinical information may be passed on to researchers within the UK. It would not be possible to

identify any patient from this information and any information provided will be handled according to the normal standard of medical confidentiality and data protection.

6.5.6. Data collection and security procedures

Where possible, data will be captured and transferred between the local hospitals and the BCPP Study Office via BCPP laptops operating on a virtual private network (VPN). The VPN will enable the laptops to log directly onto the dedicated BCPP server, located at the University of Birmingham. The VPN will be accessible only to authorised BCPP personnel when the username and password is entered in conjunction with a corresponding RSA fob number (randomly generated constantly changing password number). The VPN will transmit snapshot keystroke data via HTTPS secure web-pages using 128 mega-bit encryption. The use of a VPN ensures that no patient data will be stored on laptop hard-drives. The security afforded by this system will afford is on a par with that of UK online banking systems.

Data stored on the dedicated BCPP area of a server within the University of Birmingham will be protected by firewalls at both university and server room level. Load-balancing between two sites will be implemented to ensure that the VPN will function in the multi-user environment and will provide complete redundancy. The server rooms will only be accessible by authorised key holders within the IT team. The building in which the servers are located will additionally be restricted to authorised university personnel. Back-up tapes will be taken daily and will be stored in an onsite fireproof safe located within the server building.

The electronic transfer of encrypted data would, in all cases, be backed-up by a printed hard-copy. One copy will be sent to the BCPP Study Office and another copy will be stored locally in the site-file. The site file will be kept within a locked office accessible only to authorised hospital personnel.

Where electronic data transfer is not possible, a paper-based system will be implemented. The paper-based system requires completed paper copies of questionnaires and CRF’s to be sent to the BCPP Study Office for data entry.

Transportation of all printed documents containing patient identifiable data will be undertaken according to local trust policy on postage of confidential data.

6.5.7. Use of translation / interpreters

Depending on local guidelines, patients who do not have a good understanding of English will not be excluded from the study. However, we do not plan to provide written translations of

questionnaires as no validation testing has been undertaken to ensure that translation does not affect the semantics of the questions. Also, when completing the questionnaires, the patient must be able to communicate verbally with the research nurse to ask for clarification and to respond to questions. Where completion of the questionnaires can be achieved with the help of interpreters this will be encouraged.

If interpreters are used, it is important that they are available at each consultation and study visit as accurate and adequate information is required from the patient as part of the study procedures. If family members are used and/or NHS interpreters, this requirement will be discussed and it would be the local investigator's responsibility to arrange these services prior to patient entry into the study.

Patients unable to participate in the observational research (Project A1 and B) would still be considered eligible for the associated molecular pathology studies as well as the linked SELENIB trial.

6.5.8. Monitoring and quality assurance

100% of patient informed consent forms will be monitored by site visits and / or forwarding of copies to the BCPP Study Office.

Trial conduct will be monitored by regular site visits by the Study Co-ordinator and/or other members of the BCPP Working Group. These site visits will include source data verification of a random sample of patients' files. This process will involve comparing data contained in the CRF with source data from the medical notes. This will be undertaken for approximately 10% of patients.

Data submitted via CRF's will be subject to systematic validation at the point of data entry. Manual checking will also be undertaken centrally within the BCPP Study Office. In addition, there will be periodic clinician reviews of CRF data.

To enhance the quality and validity of questionnaire data, a printed copy of the completed questionnaires will be verified by the patient who will sign and date the printed copy on the day of completion. The verified paper copy will be forwarded to the BCPP Study Office for routine manual checking and secure storage. Crosschecks will be undertaken to compare electronic data with the printed hard-copy on a random sample of approximately 10% of patients.

6.5.9. Access to BCPP data

BCPP data may be made available to individuals subject to ethical approval and permission from the BCPP Working Group. Requests to the BCPP Study Office should include the following information:

- Reason for data request, specifying question(s) to be answered
- Documentary evidence of ethical approval of the research project.
- Required data (item and in which form)
- Proposed method to be used when working with the data (define file specification).
- Whether there are any objections to involvement of the BCPP statistician. If so outline the reason for the objections.
- Which other committees are informed/involved.
- If the data are going to be published, an agreement to name those people relevant to the study
- An agreement to adhere to the BCPP publication policy and to notify the BCPP Working Group of any resulting publications.

6.5.10. Access to Specimens within the BCPP Tumour Bank

Access to specimens stored within the BCPP Tumour Bank may be made available to individuals subject to ethical approval and permission from the BCPP Working Group. Requests to the BCPP Study Office should include the following information:

- Reason for specimen request, specifying question(s) to be answered
- Documentary evidence of ethical approval of the research project.
- A clear description of which specimens are needed.
- Proposed method to be used when working with the specimens (define laboratory technique and tests to be performed).
- Whether additional clinical data are required (if so, please also refer to section 6.5.9)
- Which other committees/organisations are involved.
- If the resulting data are going to be published, an agreement to name those people relevant to the study
- An agreement to adhere to the BCPP publication policy and to notify the BCPP Working Group of any resulting publications.

6.5.11. Publication Policy

Publication using data from the BCPP must be agreed between the main author of the manuscript or abstract and the full BCPP Working Group before starting the work, so that authorship can be discussed within this group prior to preparation of any publications. All

manuscripts and abstracts and other documents that contain data arising from the BCPP must be submitted to the BCPP Working Group at least 21 days prior to the deadline for conference submission. All abstracts and papers must have written approval from the study management committee prior to final submission.

All persons designated as authors should qualify for authorship. Every author should have participated sufficiently in the work to take public responsibility for the content. At an appropriate place in the article one or more statements should specify contributions that need acknowledging for general support, technical help, financial and material support etc.

6.5.12. NCRI Bladder Cancer Studies Group

The NCRI Bladder Cancer Studies Group will be kept informed of progress with the project and the linked SELENIB trial (Project A2). Both this project and the SELENIB trial have NCRI approval by virtue of the peer-reviewed funding from CRUK.

APPENDIX 1
PATIENT INFORMATION SHEETS AND CONSENT FORM

1. Patient Information Sheet (Version Number: 1.0 November 2005)

2. Patient Consent Form (Version Number 1.0 – November 2005)

3. General Practitioner Information Sheet (Version Number 1.0 November 2005)

TO BE PRINTED ON INSTITUTION HEADED PAPER

Bladder Cancer Prognosis Programme (BCPP)
Cancer Research UK Bladder Cancer Research Group

**Chief Investigator:**	**Professor KK Cheng**<br>**The University of Birmingham**
**Name of your doctor:**	***<Name of urologist>***<br>***<Hospital name>***

PATIENT INFORMATION SHEET
(Version Number: 1.0 November 2005)

We would like to invite you to take part in a clinical research study.

We are currently recruiting patients who have a bladder abnormality that is suspicious of bladder cancer.

Taking part in this study is entirely voluntary. Before you decide whether you would like to take part, it is important for you to understand why the research is being done and what it will involve. Please take time to read the following information carefully and discuss it with others if you wish. Ask us if there is anything that is not clear or if you would like more information. Take time to decide whether or not you wish to take part.

Thank you for reading this information leaflet.

1. What is the purpose of the study?

For the majority of patients who are diagnosed with bladder cancer, regular check-ups are required for the rest of their lives, so that if the cancer returns it can be detected early. These check-ups involve an examination of the bladder using a thin tube with a light inside that is inserted into the bladder through the water pipe (urethra). This procedure is called cystoscopy.

The dilemma for Urologists treating bladder cancer is that some bladder cancers need very frequent cystoscopies and others can be considered to be less harmful. Unfortunately, our current tests do not yet allow us to accurately predict at the outset how a patient's bladder cancer will behave in the future.

Here in the West Midlands we are undertaking a programme of research (funded by Cancer Research UK) to investigate if we can improve our ability to predict how a patient's bladder cancer will behave. This research will involve studies on your blood, urine, bladder tissue and nail clippings (these allow us to measure levels of various natural minerals contained within the body). We will use these samples to study some genetic and biological factors. We will also collect some personal details and lifestyle information using questionnaires. This information will help us to look into factors that can influence the way in which bladder cancers behave.

In order to find out if any of these factors are important, we will need to monitor your progress over the next 5 years and collect information from you regularly during your routine follow-up visits to hospital.

2. Why have I been chosen?

You have been diagnosed with a bladder abnormality that is suspicious of bladder cancer. We are hoping to collect information on patients who have been newly-diagnosed with a bladder abnormality that is suspicious of bladder cancer and who meet the requirements of the study. We intend to study at least 3400 patients over the next 3-5 years.

3. Do I have to take part?

It is up to you to decide whether or not to take part. You do not have to decide straight away. You should take this information sheet away with you. When you return to hospital before your operation, you will be asked if you would like to participate in the study. If you agree to take part you will be asked to sign a consent form. If you do decide to take part you are still free to withdraw at any time and without giving a reason. A decision to withdraw at any time, or a decision not to take part, will not affect the standard of care you receive or your relationship with your doctor.

4. What will happen to me if I take part?

Once you have agreed to take part and have signed a consent form, the study will run alongside your standard bladder cancer treatment. When you come into hospital before your bladder operation, our research nurse will talk to you about the study and will take you through a questionnaire. The questions will be on your background, medical history and lifestyle. We would need you to give us about an hour of your time to fill in this questionnaire. Before your operation we will take samples of your blood and urine and nail clippings.

During your operation pieces of tissue from your growth are removed from the lining of the bladder. This tissue will be sent to the pathologist at your hospital for detailed analysis under a microscope as part of your standard care. For the purposes of this study we will use a small sample of the tissue that is removed.

If the microscope analysis confirms that you have bladder cancer and if the cancer is suitable, we would then like to collect information from your medical records on any further bladder cancer treatment that you receive. After your operation we will send you a questionnaire which asks about your family history of cancer, your previous occupations and the places where you have lived. This questionnaire should take you about half an hour to fill in. We will also send you a food diary so that you can keep a record of what you eat and drink in a one week period.

We will monitor your progress at your regular hospital follow-up visits. This monitoring would involve completing a short questionnaire with the research nurse at each follow-up visit. This follow-up questionnaire will require approximately half an hour of your time to fill it in. If it is not possible to fill it all in when you attend your follow-up appointment, we may need to telephone you at home to collect the rest of the information. If your bladder cancer returns we would once again like to take samples of your blood and urine and bladder cancer tissue. We would like to monitor you in this way for a total of 5 years.

Following your operation and depending on the outcome of the pathologist's diagnosis, we may also wish to ask you to take part in a randomised trial (the research nurse will discuss this with you, if appropriate). If, after your operation, you are not diagnosed as having a bladder cancer, we will not ask you to participate further in this research project,

5. What do I have to do?

Other than your normal treatment, we would need you to complete our questionnaires and let us have samples of your blood, urine and nail clippings. You would also be asked to give

permission for us to use some samples of the abnormal bladder tissue that is removed during your operation.

6. What will happen to the tissue and blood samples taken as part of this study?

The bladder tissue, blood, urine and nail samples that will be collected as part of this research study will be stored centrally at a laboratory at The University of Birmingham.

We aim to collect together, from all of the patients who enter this research study, a large ‘bank’ of bladder tumour samples. The samples collected will be used, first and foremost, for research as part of the BCPP study. Such a collection or ‘bank’ of samples may also be very useful for research in the future that will help us to understand more about how bladder cancers behave. We would like to store and later use the samples donated as part of this study for future research, although such research projects have not yet been planned and could occur many years in the future. These future research projects may involve studies of your genes and DNA. By giving your consent for your bladder tissue, blood, urine and nail samples to be stored in the ‘bank’ you will be offering your samples as a gift. If, after your operation you are diagnosed as not having bladder cancer, we would still like to keep your samples for further use in approved research.

The sample stored for research will be taken from samples that remain after all the information needed by doctors diagnosing and caring for you have been obtained. The tumour, blood and urine samples are stored under strict security and are given a code, so that researchers receiving the samples do not know your name or any other personal details. Researchers who wish to use the samples that are stored in the bank will only be given access to the samples after their research has been approved by an independent Research Ethics Committee who make sure that the research is in the interest of patients and is carried out safely.

7. What are the possible disadvantages and risks of taking part?

There are no foreseeable risks of taking part. This study will run alongside your routine bladder cancer treatment and follow-up; it will not influence this process.

8. What are the possible benefits of taking part?

There is no intended immediate clinical benefit from taking part in this study. However, the information obtained from this study may result in changes in the future diagnosis, treatment, and follow-up of patients with bladder cancer. These changes may also benefit you.

9. What if new information becomes available?

This study does not influence your routine bladder cancer treatment and follow-up. However, any new discoveries or information relating to this will automatically be incorporated into the standard treatment provided by your doctors.

10. What happens when the research study stops?

When the study stops your routine bladder cancer treatment and follow-up will continue in the normal way, although it may incorporate new discoveries or information generated by this study.

11. What if something goes wrong?

As this study does not influence your routine bladder cancer treatment and follow-up, the normal National Health Service complaints mechanisms should be followed.

12. Will my taking part in this study be kept confidential?

All information which is collected about you during the course of the research will be kept strictly confidential. If you agree to take part in this study we will need you to sign a consent form. You will be given a copy of the consent form and this information sheet to keep.

With your consent, we will be informing your GP about your participation in this study. Authorised professionals, other than those involved directly in your care, may inspect your medical notes. Information contained within your medical notes would be used only for the purposes of collecting information about your treatment and to check that the research is being carried out correctly. These authorised professionals include members of the BCPP (Bladder Cancer Prognosis Programme) research team and regulatory authority representatives.

We would like to collect some contact details from you including your current address, and telephone number. We would like to collect these details so that we can send a questionnaire to you at home and so that we can contact you before an appointment to remind you if you need to bring anything in with you to the hospital. Your contact details will be kept strictly confidential and only members of the BCPP research team would be allowed access to them.

Information on all patients entered into this study will be sent to the BCPP Study Office which is located at The University of Birmingham where it will be retained in secure storage and handled according to the 1998 Data Protection Act. No personally identifiable information will be released from the BCPP study office. Limited clinical information may be passed on to researchers within the UK. It would not be possible to identify any patient from this information and any information provided will be handled according to the normal standard of medical confidentiality and data protection.

13. What will happen to the results of the research study?

Important results from the study will be published as they become available, which may be during the course of the study or after the study has finished, and this could possibly take several years. We intend that any results will be published in peer-reviewed journals or will be presented at meetings involved with this field of cancer research, and these publications will be available upon request from your specialist doctor. You will not be identified in any report or publication.

14. Who is organising and funding the research?

The research is being organised by The Department of Public Health and Epidemiology at The University of Birmingham, in collaboration with the Cancer Research UK Institute for Cancer Studies at The University of Birmingham, The Department of Urology at The Queen Elizabeth Hospital, Birmingham, and all participating Urology Departments within the West Midlands. The research is funded by Cancer Research UK. The doctors conducting this study are not being paid for including and looking after you within this study.

15. Who has reviewed the study?

This study has been reviewed by the Nottingham Multi-centre Research Ethics Committee and by scientific experts at Cancer Research UK.

16. What if I have other concerns or would like further information?

If you have any concerns or other questions about this study or the way it has been carried out, you should contact the Urologist or the BCPP Research Nurse listed below. If you would like advice from someone independent of the study concerning your participation, you should contact <e.g. Urology CNS>.

Finally, thank you for taking the time to read this information sheet and for taking part in the study, if you agree to do so.

Contact Details:

Urologist:<Local investigator> Telephone: <Secretary Contact Number>

BCPP Research Nurse: <Research nurse> Telephone: <Research nurse mobile>

TO BE PRINTED ON INSTITUTION HEADED PAPER

PATIENT CONSENT FORM
(Version Number 1.0 – November 2005)

Title of Project: Bladder Cancer Prognosis Programme (BCPP)

Centre Name: *<Name of Hospital>*
Patient Number: *<Study Number>*

Name of Researcher: ***<Name of Urologist>***

Please initial Inside the box

1. I confirm that I have read and understand the information sheet(s) dated *<Date>* (version *<Number>*) for the above study and have had the opportunity to ask questions. ☐
2. I understand that my participation is voluntary and that I am free to withdraw at any time, without giving a reason, without my medical care or legal rights being affected. ☐
3. I understand that my medical notes may be looked at by members of the BCPP research team and regulatory authority representatives, but understand that strict confidentiality will be maintained. ☐
4. I agree to take part in the above Bladder Cancer Prognosis Programme. ☐
5. I agree for samples of my bladder tissue, blood and urine and toenail clippings to be stored and used for current and future biological research projects which have received appropriate scientific and ethical approval. ☐
6. I consent to my GP being informed of my participation in this study. ☐
7. I agree for my contact details to be stored and used to contact me about aspects of the study either by telephone or by post. ☐

Name of patient	Date	Signature
Name of person taking consent	Date	Signature

(1 for patient; 1 for researcher; 1 to be kept with hospital notes)

- TO BE PRINTED ON INSTITUTION HEADED PAPER

Bladder Cancer Prognosis Programme (BCPP)
Cancer Research UK Bladder Cancer Research Group

**Chief Investigator:**	**Professor KK Cheng**<br>**The University of Birmingham**
**Local Investigator:**	***<Name of urologist>***<br>***<Hospital name>***

GENERAL PRACTITIONER INFORMATION SHEET
(Version Number 1.0 November 2005)

Dear Colleague,

Your patient ……………………… (date of birth ….../...…/………) has been diagnosed with a bladder abnormality, which is suspected to be bladder cancer following initial clinical investigation. We have invited your patient to take part in a clinical research study. The name of this research study is the Bladder Cancer Prognosis Programme (BCPP). The study aims to examine various lifestyle, environmental and biological factors which may be associated with an increased risk of tumour recurrence or progression.

We attach a copy of the information sheet that has been given to your patient

If you wish to obtain any further information, please contact the BCPP Research Nurse or the local investigator (contact details are given below).

Research Nurse:
<Name of Research Nurse> <BCPP mobile number>

Local Investigator:
<Name of local investigator> <Telephone of secretary>

Yours sincerely

<Insert name of local investigator>

APPENDIX 2
BASELINE, POSTAL AND FOLLOW-UP QUESTIONNAIRES

- **Initial Questionnaire**
  (Completed at diagnosis)

- **Postal Questionnaire**
  (Posted to the patient 2 weeks prior to first follow-up, returned by the patient by hand at the follow-up appointment)

- **Food and Micturition Diary**
  (Posted to the patient 2 weeks prior to first follow-up, returned by the patient by hand at the follow-up appointment)

- **Follow-up Questionnaire**
  (Completed at 1st routine follow-up and annual follow-up visits)

Study Number: ☐☐☐☐

BLADDER CANCER PROGNOSIS PROGRAMME

Cancer Research UK Bladder Cancer Group

Initial Questionnaire

<u>CONFIDENTIAL</u>

VERSION: FINAL 1.0 November 2005

PLEASE DESTROY PREVIOUS VERSIONS

MREC APPROVAL: 23rd November 2005

START DATE: 1st January 2006

A programme of research

Conducted by: UNIVERSITY OF BIRMINGHAM

Funded by: CANCER RESEARCH UK

INTRODUCTION

In order to provide better health care for bladder cancer patients, we hope to discover more about how bladder cancer is related to people's circumstances. We are particularly interested in what we can do to prevent bladder cancers from coming back after they have been treated.

The questions that you will be asked will include questions about your lifestyle, your behaviours, your health and the help and support you receive from the people around you.

There may be some questions that you think are unusual. The questions are not used to test you in any way and the responses that you give will not be used to make any judgements of you. There are no right or wrong answers. All of your responses will be treated as strictly confidential and will be used only for medical research.

Some of the questions ask for personal information. If you feel that any of the questions are too personal, do not answer them. However, by answering these questions, you will help us to discover links between lifestyles and health.

I <Research Nurse> will take you through each question and keep a record of your responses. There is no time limit, so if you want a little time to think about any of the questions please do so. Try to answer every question even if the answer is 'I can't remember' or 'I don't know'.

If you have any other questions, or if there is anything that you feel you don't understand please ask me <Research Nurse> at any time.

Thank you for taking the time to participate in this study

CANCER RESEARCH UK	BLADDER CANCER PROGNOSIS PROGRAMME (BCPP)	INITIAL QUESTIONNAIRE<br><br>**PERSONAL DETAILS**

Patient Identifier:<br>*First 3 letters from surname followed by first 2 letters from forename*			☐☐☐☐☐<br>*eg: John Smith = SMIJO*
Date of Birth:<br><br>Sex: *(Please circle)*	D D M M Y Y Y Y<br><br>**M** **F**	Responsible Urologist:<br><br>Hospital No:	________________<br><br>________________
Date of Interview:<br><br>Time of Interview<br>*(24hour)*	D D M M Y Y Y Y<br><br>☐☐ : ☐☐	Name of Interviewer:<br><br>Hospital Name:	________________<br><br>________________
Will the interview take place with the support of a translator			Yes ☐ No ☐

SECTION 1: CONTACT DETAILS

1.1. What is your current postal address?

House Number and street ☐

Location (Town/City) ☐

Country ☐

Country ☐

Postcode (if known) ☐

1.2. What is your current home telephone number? ☐☐☐☐☐-☐☐☐☐☐☐☐
(Including dialling code)

1.3. What is your current mobile telephone number, if you have one? ☐☐☐☐☐☐☐☐☐☐☐

1.4. What is the name of your GP? Dr. ☐

1.5. What is the name of you GP practice? ☐

1.6. What is the practice address?

Number and street name ☐

Location (Town/City) ☐

Postcode (if known) ☐

SECTION 2: GENERAL INFORMATION

MARITAL STATUS

2.1. What is your current marital status? (tick appropriate box)

Single ☐ Married / living with partner ☐ Widowed ☐ Separated ☐ Divorced ☐

ETHNIC ORIGIN

2.2. To which of these groups do you consider you belong to? (tick appropriate box)

White ☐ Black, Caribbean ☐ Black, other ☐ Chinese ☐ Pakistani ☐

Indian ☐ Bangladeshi ☐ Other ☐ 'Other' specify ____________

EDUCATION AND QUALIFICATIONS

2.3. How old were you when you left school? ☐☐

2.4. Do you have any of the following qualifications? *(Tick all applicable)*

School Leaving Certificate	☐	Technical College Exams / City & Guilds	☐	Trade Certificates	☐
GCE "O" LEVEL or GCSE	☐	Completed Apprenticeship	☐	Teaching Diploma, HNC	☐
CSE	☐	Higher National Diploma (HND)	☐	Secretarial College Exams	☐
"A" Level, Highers	☐	Matriculation (University entry exam)	☐	University Degree	☐

Other ☐ *'Other' describe:* ______________________________

SECTION 3: SMOKING BEHAVIOUR

When completing the following questions, please indicate your **CURRENT** smoking behaviours

When answering these questions, the term 'Never smoked' means that you have never smoked **or** have smoked on less than ten occasions during your lifetime.

3.1. Do you currently smoke any form of tobacco? *(tick appropriate box)*

- Yes ☐ *Please complete **PARTS A and B.***
- No, but I used to smoke ☐ *Please complete **PARTS A and B.***
- No, I have never smoked ☐ *Please complete **PART B** only.*

PART A – This section is to be completed by current and ex-smokers only.

FILTER CIGARETTES

3.2. Do you currently smoke filter cigarettes? *(tick appropriate box)*

- Yes ☐ ***Go to question 3.5***
- No, but I used to smoke filter cigarettes ☐ ***Go to question 3.3***
- No, I have never smoked filter cigarettes ☐ ***Go to question 3.10***

3.3. How old were you when you stopped smoking filter cigarettes completely? |__|__| *Years old*

3.4. How old were you when you stopped smoking filter cigarettes regularly? |__|__| *Years old*

3.5. How old were you when you started smoking filter cigarettes? |__|__| *Years old*

3.6. How old were you when you started smoking filter cigarettes daily? |__|__| *Years old*

Never smoked daily ☐

3.7. How many filter cigarettes do/did you smoke on average per week? ☐☐☐

3.8. What brand of filter cigarettes do/did you usually use? ___________________________

3.9. How deep do/did you usually inhale the smoke? *(tick appropriate box)*

- Mouth only ☐
- Throat ☐
- Lung ☐

SMOKING BEHAVIOUR *(Continued)*

NON FILTER CIGARETTES

3.10. Do you currently smoke non filter cigarettes? *(tick appropriate box)*

- Yes ☐ ***Go to question 3.13***
- No, but I used to smoke non filter cigarettes ☐ ***Go to question 3.11***
- No, I have never smoked non filter cigarettes ☐ ***Go to question 3.18***

3.11. How old were you when you stopped smoking non-filter cigarettes completely? |__|__| *Years old*

3.12. How old were you when you stopped smoking non-filter cigarettes regularly? |__|__| *Years old*

3.13. How old were you when you started smoking non-filter cigarettes? |__|__| *Years old*

3.14. How old were you when you started smoking non-filter cigarettes daily? |__|__| *Years old*

Never smoked daily ☐

3.15. How many non filter cigarettes do/did you smoke on average per week? ☐☐☐

3.16. What brand of non filter cigarettes do/did you usually use? ______________________

3.17. How deep do/did you usually inhale the smoke? *(tick appropriate box)*

- Mouth only ☐
- Throat ☐
- Lung ☐

HAND ROLLED CIGARETTES

3.18. Do you currently smoke hand-rolled cigarettes? *(tick appropriate box)*

- Yes ☐ ***Go to question 3.21***
- No, but I used to smoke hand-rolled cigarettes ☐ ***Go to question 3.19***
- No, I never smoked hand-rolled cigarettes ☐ ***Go to question 3.25***

3.19. How old were you when you stopped smoking hand-rolled cigarettes completely? |__|__| *Years old*

3.20. How old were you when you stopped smoking hand-rolled cigarettes regularly? |__|__| *Years old*

3.21. How old were you when you started smoking hand-rolled cigarettes? |__|__| *Years old*

SMOKING BEHAVIOUR *(Continued)*

HAND-ROLLED CIGARETTES *(Continued)*

3.22. How many hand-rolled cigarettes do/did you smoke on average per week? ☐☐☐

3.23. What brand of hand-rolled cigarettes do/did you usually use? ______________________

3.24. How deep do/did you usually inhale the smoke? *(tick appropriate box)*

- Mouth only ☐
- Throat ☐
- Lung ☐

CIGARS

3.25. Do you currently smoke cigars? *(tick appropriate box)*

- Yes ☐ ***Go to question 3.28***
- No, but I used to smoke cigars ☐ ***Go to question 3.26***
- No, I have never smoked cigars ☐ ***Go to question 3.32***

3.26. How old were you when you stopped smoking cigars completely? |__|__| *Years old*

3.27. How old were you when you stopped smoking cigars regularly? |__|__| *Years old*

3.28. How old were you when you started smoking cigars? |__|__| *Years old*

3.29. How many cigars do/did you smoke on average per week? ☐☐☐

3.30. What brand of cigars do/did you usually use? ______________________________

3.31. How deep do/did you inhale the smoke? *(tick appropriate box)*

- Mouth only ☐
- Throat ☐
- Lung ☐

PIPE

3.32. Do you currently smoke a pipe? *(tick appropriate box)*

- Yes ☐ ***Go to question 3.35***
- No, but I used to smoke a pipe ☐ ***Go to question 3.33***
- No, I have never smoked a pipe ☐ ***Go to question 3.39***

SMOKING BEHAVIOUR *(Continued)*

PIPE *(Continued)*

3.33. How old were you when you stopped smoking a pipe completely? |__|__| *Years old*

3.34. How old were you when you stopped smoking a pipe regularly? |__|__| *Years old*

3.35. How old were you when you started smoking a pipe? |__|__| *Years old*

3.36. How many pipes do/did you smoke on average per week? ☐☐☐

3.37. What brand of tobacco do/did you usually use in your pipe? ______________________

3.38. How deep do/did you usually inhale the smoke? *(tick appropriate box)*
- Mouth only ☐
- Throat ☐
- Lung ☐

PART B – This section is to be completed by all patients

PASSIVE SMOKING

3.39. During your childhood (before you were 18), did you live with someone who smoked? *(tick appropriate box)*
- Yes ☐
- No ☐ ***Go to question 3.41***

3.40. For how many years did you live with smokers during your childhood? |__|__| *Years*

3.41. During your adult life (after you were 18), have you lived with someone who smoked? *(tick appropriate box)*
- Yes ☐
- No ☐ ***Go to question 3.44***

3.42. For how many years did you live with smokers during your adulthood? |__|__| *Years*

3.43. What was the usual duration of your exposure? |__|__| *Hours per day*

3.44. Have you ever been exposed during your indoor work to co-workers who smoked in the same room? *(tick appropriate box)*
- Yes ☐
- No, not exposed indoors ☐ ***Go to question 4.1***
- No, I have never worked indoors ☐ ***Go to question 4.1***

3.45. For how many years have you been exposed to smoking co-workers indoors? |__|__| *Years*

3.46. What was the usual duration of your exposure? |__|__| *Hours per day*

3.47. How frequently are you exposed to other people's tobacco smoke in public places (e.g. shopping, restaurants, bars) for a minimum of 5 consecutive minutes?
|__|__| *Times per week*

SECTION 4: DIETARY BEHAVIOURS

The questions in this section ask about your normal diet during the last year

4.1. Please indicate how often on average, **during the past year**, you have eaten each of the food types that are listed below

	Average Use Last Year					
	Never or less than once per month	1-3 per month	Once a week	2-4 per week	5-6 per week	At least once per day
**STAPLE FOODS**						
Bread ………………………………………………..	☐	☐	☐	☐	☐	☐
Potatoes……………………………………….…..	☐	☐	☐	☐	☐	☐
Pasta……………………………………………<br>(eg. Macaroni, Spaghetti)	☐	☐	☐	☐	☐	☐
Rice ……………………………………………	☐	☐	☐	☐	☐	☐
Noodles………………………………………	☐	☐	☐	☐	☐	☐
Wheat …………………………………………….<br>(eg. Whole grain bread)	☐	☐	☐	☐	☐	☐
Cereal……………………………………………..<br>(eg. Oats, bran, corn)	☐	☐	☐	☐	☐	☐
**MEAT**						
Meat (no organs)………………………………..<br>(eg. Pork, Steak, Beef, Lamb)	☐	☐	☐	☐	☐	☐
Organ Meat…………………………………….<br>(eg. Liver, Heart, Kidney)	☐	☐	☐	☐	☐	☐
Chicken………………………………………..	☐	☐	☐	☐	☐	☐
Other Poultry………………………………….<br>(eg. Goose, Duck, Pigeon)	☐	☐	☐	☐	☐	☐
**FISH**						
Dark Fleshed Fish………………………………<br>(eg. Mackerel, Salmon, Anchovies, Sardines, Trout, Tuna, Herring)	☐	☐	☐	☐	☐	☐
White fleshed fish ………………………………<br>(eg. Cod, Haddock, Hake, Halibut, Plaice, Seabass, Skate, Sole)	☐	☐	☐	☐	☐	☐
Seafood………………………………………<br>(eg. Prawn, Crab, Lobster, Cockles, Winkles, Squid, Octopus, Mussels)	☐	☐	☐	☐	☐	☐
**VEGETABLES**						
Fruit Vegetables…………………………<br>(eg. Tomato, Cucumber, Aubergine)	☐	☐	☐	☐	☐	☐

DIETARY BEHAVIOURS

	**Average Use Last Year**					
	**Never or less than once per month**	**1-3 per month**	**Once a week**	**2-4 per week**	**5-6 per week**	**At least once per day**
VEGETABLES *(Continued)*						
Flower vegetables………………………… (eg. Broccoli, Cauliflower)	☐	☐	☐	☐	☐	☐
Leafy vegetables…………………………… (eg. Spinach, Cabbage, Lettuce)	☐	☐	☐	☐	☐	☐
Stem vegetables…………………………… (eg. Asparagus, Celery, Fennel)	☐	☐	☐	☐	☐	☐
Mushrooms…………………………………	☐	☐	☐	☐	☐	☐
Bulbs……………………………………… (eg. Onion, Garlic, Leek, Shallot)	☐	☐	☐	☐	☐	☐
Roots……………………………………… (eg. Beetroot, Swede, Carrot, Parsnip)	☐	☐	☐	☐	☐	☐
FRUIT						
Citrus Fruits……………………………… (eg. Orange, Lemon, Lime, Grapefruit)	☐	☐	☐	☐	☐	☐
Stone Fruits……………………………… (eg. Plum, Apricot, Peach, Cherry)	☐	☐	☐	☐	☐	☐
Soft Fruits………………………………… (eg. Raspberry, Strawberry, Redcurrant, Blackberry)	☐	☐	☐	☐	☐	☐
Fleshy Fruits……………………………… (eg. Apple, Pear, Banana, Pineapple)	☐	☐	☐	☐	☐	☐
Vine Fruits………………………………… (eg. Grape, Melon, Cantaloupe)	☐	☐	☐	☐	☐	☐
DAIRY						
Cream………………………………………	☐	☐	☐	☐	☐	☐
Butter / Margarine…………………………	☐	☐	☐	☐	☐	☐
Yogurt………………………………………	☐	☐	☐	☐	☐	☐
Cheese ……………………………………	☐	☐	☐	☐	☐	☐
Egg …………………………………………	☐	☐	☐	☐	☐	☐
OTHER FOODS						
Pulses……………………………………… (eg. Pea, Bean, Lentil)	☐	☐	☐	☐	☐	☐
Nuts and Seeds……………………………	☐	☐	☐	☐	☐	☐
Soy/Tofu products………………………… (eg. Soy milk, Tofu, Soya meat)	☐	☐	☐	☐	☐	☐
Sweets and snacks………………………… (eg. Crisps, cakes, chocolate)	☐	☐	☐	☐	☐	☐

TEA DRINKING

4.2. Which of the following describes your current tea consumption best?

- I drink tea ☐
- I used to drink tea, but I don't anymore ☐
- I have never drunk tea ☐ ***Go to question 4.8***

4.3. At what age did you start drinking tea? ⎵⎵ *Years old* For as long as I can remember ☐

4.4. At what age did you stop drinking tea? ⎵⎵ *Years old* Have not stopped ☐

4.5. What strength of tea do/did you usually prefer to drink? (tick appropriate box)

- Weak ☐
- Moderate ☐
- Strong ☐
- Very Strong ☐

4.6. Do/did you add milk to your tea? (tick appropriate box)

- Never ☐
- Rarely ☐
- Sometimes ☐
- Usually ☐
- Always ☐

4.7. Do/did you add sugar to your tea? (tick appropriate box)

- Never ☐
- Rarely ☐
- Sometimes ☐
- Usually ☐
- Always ☐

COFFEE DRINKING

4.8. Which of the following describes your current coffee consumption best?

- I drink coffee ☐
- I used to drink coffee, but I don't anymore ☐
- I have never drunk coffee ☐ ***Go to question 4.14***

4.9. At what age did you start drinking coffee? ⎵⎵ *Years old* For as long as I can remember ☐

4.10. At what age did you stop drinking coffee? ⎵⎵ *Years old* Have not stopped ☐

COFFEE DRINKING *(Continued)*

4.11. What strength of coffee do/did you usually prefer to drink? (tick appropriate box)

- Weak ☐
- Moderate ☐
- Strong ☐
- Very Strong ☐

4.12. Do/did you add milk to your coffee? (tick appropriate box)

- Never ☐
- Rarely ☐
- Sometimes ☐
- Usually ☐
- Always ☐

4.13. Do/did you add sugar to your coffee? (tick appropriate box)

- Never ☐
- Rarely ☐
- Sometimes ☐
- Usually ☐
- Always ☐

ALCOHOL DRINKING

4.14. Which of the following describes your alcohol consumption best?

- I drink alcohol ☐
- I used to drink alcohol, but I don't anymore ☐
- I have never drunk alcohol ☐ ***Go to question 4.18***

4.15. How many days a week do/did you usually drink alcohol? ☐

4.16. At what age did you start drinking alcohol? ☐☐ *Years old*

4.17. At what age did you stop drinking alcohol? ☐☐ *Years old* Have not stopped ☐

FLUID INTAKE

4.18. Please indicate how often, during the past year, you have drunk one measure each of the types of drinks that are listed below.

	**Measure**	**Average Use Last Year**<br>Never or less than one measure per month	1-3 per month	One a week	2-4 per week	5-6 per week	At least one per day	How many per day?
ALCOHOLIC DRINKS								
Wine or champagne………	1 small glass	☐	☐	☐	☐	☐	☐ →	⊔⊔
Fortified Wine………………. (eg. Port, Sherry, Cinzano)	1 small glass	☐	☐	☐	☐	☐	☐ →	⊔⊔
Beer…………………………. (eg. Beer, Lager, Stout)	1 Pint	☐	☐	☐	☐	☐	☐ →	⊔⊔
Cider…………………………	1 Pint	☐	☐	☐	☐	☐	☐ →	⊔⊔
Spirits……………………….. (eg. Gin, Brandy, Rum, Vodka, Whisky)	1 pub measure (25cl)	☐	☐	☐	☐	☐	☐ →	⊔⊔
Liqueurs…………………….. (eg. Tia Maria, Cointreau, Baileys, Grand Marnier, etc)	1 pub measure (25cl)	☐	☐	☐	☐	☐	☐ →	⊔⊔
HOT DRINKS								
Coffee………………………..	1 cup	☐	☐	☐	☐	☐	☐ →	⊔⊔
Tea…………………………..	1 cup	☐	☐	☐	☐	☐	☐ →	⊔⊔
Hot Chocolate……………..	1 cup	☐	☐	☐	☐	☐	☐ →	⊔⊔
Ovaltine / Horlicks…………..	1 cup	☐	☐	☐	☐	☐	☐ →	⊔⊔
Soup…………………………	1 Cup/ bowl	☐	☐	☐	☐	☐	☐ →	⊔⊔
SOFT DRINKS								
Fizzy pop…………………… (eg. Lemonade, Cola)	½ pint glass	☐	☐	☐	☐	☐	☐ →	⊔⊔
Pure fruit juice………………. (eg. Orange, Apple, etc)	½ pint glass	☐	☐	☐	☐	☐	☐ →	⊔⊔
Fruit squash or cordial……..	½ pint glass	☐	☐	☐	☐	☐	☐ →	⊔⊔
Milk…………………………..	½ pint glass	☐	☐	☐	☐	☐	☐ →	⊔⊔
Water ………………………..	½ pint glass	☐	☐	☐	☐	☐	☐ →	⊔⊔

SECTION 5: OTHER BEHAVIOURS

ARTIFICIAL SWEETENERS (SUGAR SUBSTITUTES)

5.1. Which of the following describes your artificial sweetener consumption best?
- I use artificial sweeteners ☐
- I used to use artificial sweeteners, but I don't anymore ☐
- I have never used artificial sweeteners ☐ ***Go to question 5.9***

5.2. At what age did you start using artificial sweeteners? ☐☐ *Years old*

5.3. At what age did you stop using artificial sweeteners? ☐☐ *Years old*
☐ Have not stopped

5.4. What is/was the name of your regular brand of sweetener? ____________________

5.5. What form does/did this sweetener come in?
- Pill or tablet ☐
- Granulated powder ☐
- Liquid ☐

5.6. If this sweetener comes in the form of a pill, on average, how many pills per week do/did you use? ☐☐☐

5.7. If this sweetener comes in the form of granulated powder or liquid, on average, how many teaspoons per week do/did you use? ☐☐☐

5.8. Which of the following brands of sweetener have you ever used?

Sweet'n'Low	☐	Splenda	☐
Natreen	☐	Nutrasweet	☐
Canderel	☐	Sweetex	☐
Sunnet	☐	Flix	☐
Shapers	☐	Natrena	☐
Saxin	☐	Hermesetas	☐
Diamin	☐	Other	☐

'Other' Specify? ____

VITAMINS AND SUPPLEMENTS

5.9. Which of the following describes your use of vitamins and supplements best?

- I take vitamins or supplements ☐
- I used to take vitamins or supplements, but I don't anymore ☐
- I have never taken vitamins or supplements ☐ ***Go to Question 5.11***

5.10. Have you ever taken, any of the following vitamins or supplements for a period of 3 months or more?

Type of Vitamin / Supplement	Yes	no	Unknown	What is the name of your medication?	Where do you buy this product?
Example Example Example Example Example Example					
Multi-vitamins	☐	☑	☐	*ABC Plus Tablets*	*Holland and Barrett*
Multi-vitamins	☐	☐	☐		
Folic Acid	☐	☐	☐		
Vitamin B	☐	☐	☐		
Vitamin C	☐	☐	☐		
Vitamin E	☐	☐	☐		
Iron pills	☐	☐	☐		
Cod liver oil	☐	☐	☐		
Magnesium	☐	☐	☐		
Zinc	☐	☐	☐		
Vitamin B12	☐	☐	☐		
Selenium	☐	☐	☐		
Chromium	☐	☐	☐		
Calcium	☐	☐	☐		
Other (1)	☐	☐	☐		
Other (2)	☐	☐	☐		

If you are taking any vitamins or supplements. Please bring the packet in with you on your next hospital visit

HAIR COLOURING

If you have never used either permanent or semi permanent hair colouring, Please go to question 5.15

In this section of the questionnaire, we would like you to tell us about your use of semi - permanent or permanent hair colorants to darken, lighten or change the colour of your hair.

This does not include any temporary hair dyes (dyes that last only 6 to 10 washes)

Semi permanent = Hair dyes that last at least a couple of months but eventually wash out.
Permanent = Hair dyes that do not wash out, they grow out with your hair.

5.11. Which of the following best describes your use of **semi permanent** hair colouring?

- I currently use semi permanent hair colouring ☐
- I have used semi permanent hair colouring in the past, but I don't anymore ☐
- I have never used semi permanent hair colouring ☐

5.12. Which of the following best describes your use of **permanent** hair colouring?

- I currently use permanent hair colouring ☐
- I have used permanent hair colouring in the past, but I don't anymore ☐
- I have never used permanent hair colouring ☐

5.13. Please indicate below, which **semi - permanent** colours you have **ever** applied to your hair, how long you kept your hair that colour and how often did you dye it.

Semi - permanent hair colour	Type of application	In total, for how many years did you dye your hair this colour?	Roughly how many times per year do/did you colour your hair?
Blonde	Whole head	⎿⏌⎿⏌ *years*	⎿⏌⎿⏌ *times per year*
	Sections of hair (Highlights)	⎿⏌⎿⏌ *years*	⎿⏌⎿⏌ *times per year*
Brown	Whole head	⎿⏌⎿⏌ *years*	⎿⏌⎿⏌ *times per year*
	Sections of hair (Lowlights)	⎿⏌⎿⏌ *years*	⎿⏌⎿⏌ *times per year*
Red / other bright colour	Whole head	⎿⏌⎿⏌ *years*	⎿⏌⎿⏌ *times per year*
	Sections of hair	⎿⏌⎿⏌ *years*	⎿⏌⎿⏌ *times per year*
Black	Whole head	⎿⏌⎿⏌ *years*	⎿⏌⎿⏌ *times per year*

HAIR COLOURING *(Continued)*

5.14. Please indicate below, which **permanent** colours you have **ever** applied to your hair, how long you kept your hair that colour and how often did you dye it.

Permanent hair colour	Type of application	In total, for how many years did you dye your hair this colour?	Roughly how many times per year do/did you colour your hair?
Blonde	Whole head	⌴⌴ *years*	⌴⌴ *times per year*
	Sections of hair (Highlights)	⌴⌴ *years*	⌴⌴ *times per year*
Brown	Whole head	⌴⌴ *years*	⌴⌴ *times per year*
	Sections of hair (Lowlights)	⌴⌴ *years*	⌴⌴ *times per year*
Red / other bright colour	Whole head	⌴⌴ *years*	⌴⌴ *times per year*
	Sections of hair	⌴⌴ *years*	⌴⌴ *times per year*
Black	Whole head	⌴⌴ *years*	⌴⌴ *times per year*

INDUSTRIAL CHEMICALS

5.15. Please tell us whether you have ever been exposed to any of the following substances whilst at work and if so for how many years you were exposed.

SUBSTANCE	Yes	No	Unknown	If YES How Many Years were you exposed to this substance
Diesel exhaust fumes	☐	☐	☐	⌴⌴ *years*
Dyes	☐	☐	☐	⌴⌴ *years*
Metal Dust	☐	☐	☐	⌴⌴ *years*
Mineral Oil	☐	☐	☐	⌴⌴ *years*
Pesticides	☐	☐	☐	⌴⌴ *years*

SECTION 6: MEDICATIONS

Please indicate which medications you have taken. Where possible, please also record the name of the of the medication that you have taken

6.1. Have you taken any of the following medications regularly for a period of 3 months or more?

Type of Medicine	Yes	No	Unknown	What is the name of your medication?
Example Example Example Example Example Example				
NSAIDS	☑	☐	☐	***Ibuprofen***
Pain killers				
Paracetamol	☐	☐	☐	
Phenacetin	☐	☐	☐	
Aspirin	☐	☐	☐	
NSAID's (eg. Ibuprofen, Brufen, Diclofenac, Volterol)	☐	☐	☐	
Medicine for high cholesterol (eg. Simvastatin, Pravastatin, Atorvastatin)	☐	☐	☐	

6.2. Have you ever taken any of the following medications?

Type of Medicine	Yes	No	Unknown	What is the name of your medication?
Antidepressants, sleeping pills or sedatives	☐	☐	☐	
Chemotherapy	☐	☐	☐	
Immune Suppressants (eg. Steroid Tablets, Cyclosporin)	☐	☐	☐	
Inhaled Steroids	☐	☐	☐	
**Women Only:** Hormone replacement therapy (HRT)	☐	☐	☐	
**Women Only:** The contraceptive pill	☐	☐	☐	

SECTION 7: MEDICAL HISTORY

The questions in this section ask about medical conditions you might have had.

Please indicate which conditions you have ever had and which you have never had. For each condition you have had, please give your approximate age (in years) when the condition started.

7.1. Do you have, or have ever had any of the following conditions?

	Yes	No	Not Sure	IF YES, PLEASE GIVE AGE WHEN IT FIRST STARTED (in years)
BRAIN and NERVOUS SYSTEM				
Insomnia requiring treatment?......................................	☐	☐	☐	|__|__|
Depression requiring treatment?..................................	☐	☐	☐	|__|__|
HEART and CIRCULATORY SYSTEM				
Arrhythmia (Irregular heartbeat or palpitations), requiring medication or follow-up by a doctor?........... **Please describe**........................	☐	☐	☐	|__|__|
A myocardial infarction (heart attack)? ………………….	☐	☐	☐	|__|__|
Pulmonary embolism (obstruction of a blood vessel in the lungs)?..	☐	☐	☐	|__|__|
Hypertension (high blood pressure) requiring medication?...	☐	☐	☐	|__|__|
Hyperlipidaemia (High blood cholesterol)?....................	☐	☐	☐	|__|__|
Angina pectoris (chest pains due to lack of oxygen to the heart requiring medication)?........................	☐	☐	☐	|__|__|
Migraine?..	☐	☐	☐	|__|__|
Stroke?...	☐	☐	☐	|__|__|
Deep vein thrombosis (Blood clot in head, lung, arm, leg or pelvis)? …………………………………………	☐	☐	☐	|__|__|
Ischemic legs (Atherosclerosis / hardening of blood vessels in the legs)?..	☐	☐	☐	|__|__|
CHEST / ALLERGIES				
Bronchitis / emphysema? …………………………………	☐	☐	☐	|__|__|
Hay fever / eczema?..	☐	☐	☐	|__|__|
Asthma?... **Please describe**........................	☐	☐	☐	|__|__|

MEDICAL HISTORY *(Continued)*

	Yes	No	Not Sure	IF YES, PLEASE GIVE AGE WHEN IT FIRST STARTED (in years)
**DIGESTIVE SYSTEM**				
Gallstones? …………………………………………………	☐	☐	☐	|__|__|
Have you had your gall bladder removed?....................	☐	☐	☐	|__|__|
**Please describe**...				
Liver disease? ……………………………………………	☐	☐	☐	|__|__|
**Please describe**...				
Intestinal polyps?..	☐	☐	☐	|__|__|
Bilharzia (parasitic worms) of the bowel?.......................	☐	☐	☐	|__|__|
**BONES**				
Arthritis requiring drug treatment for more than 3 months?...	☐	☐	☐	|__|__|
Fracture of the wrist after age 20?................................	☐	☐	☐	|__|__|
Fracture of the spine?...	☐	☐	☐	|__|__|
Osteoporosis (brittle bones disease)?...........................	☐	☐	☐	|__|__|
Fracture of the hip?...	☐	☐	☐	|__|__|
**CONGENITAL ABNORMALITIES**				
Renal tract abnormalities?...	☐	☐	☐	|__|__|
**Please describe**...				
Any other congenital abnormality or birth defect?...........	☐	☐	☐	|__|__|
**Please describe**...				
**OTHER**				
Tuberculosis?...	☐	☐	☐	|__|__|
Psychiatric illness other than insomnia or depression?..	☐	☐	☐	|__|__|
**Please describe**...				
Diabetes controlled by diet?...	☐	☐	☐	|__|__|
Diabetes controlled by insulin injection?........................	☐	☐	☐	|__|__|
Diabetes controlled by pills or tablets?…………………...	☐	☐	☐	|__|__|
**TUMOURS**				
Benign growth (non cancer)?..	☐	☐	☐	|__|__|
**Please describe**...				
Cancer, other than your current bladder cancer?............	☐	☐	☐	|__|__|
**Please describe**...				
Radiotherapy to lower abdomen or genitals?............	☐	☐	☐	|__|__|
Any kind of chemotherapy?......................................	☐	☐	☐	|__|__|
**Please describe**...				

MEDICAL HISTORY *(Continued)*

	**Yes**	**No**	**Not Sure**	**<u>IF YES,</u> PLEASE GIVE AGE WHEN IT FIRST STARTED (in years)**
URINARY SYSTEM				
Have you ever had kidney stones?..........................	☐	☐	☐	⎵⎵
Have you ever had stones in the bladder?................	☐	☐	☐	⎵⎵
Have you had a urinary infection (cystitis) that required antibiotics on more than 1 occasion?......	☐	☐	☐	⎵⎵
Have you had any injury requiring a catheter in the bladder for more than 1 month?...........................	☐	☐	☐	⎵⎵
Have you ever had treatment for Bilharzia (schistosomiasis) of the bladder? …………………	☐	☐	☐	⎵⎵
Have you ever had treatment for genital (infective) warts?..	☐	☐	☐	⎵⎵
**Men Only:** Have you had treatment for an enlarged prostate?..	☐	☐	☐	⎵⎵
Have you had treatment for an overactive bladder?	☐	☐	☐	⎵⎵
Were you born with an abnormality that affected your bladder or your control of your bladder?<br>**Please describe**...	☐	☐	☐	

7.2. Please tell us if there are any chemicals or drugs that you have taken, that you feel may have contributed to your developing bladder cancer?

____

____

____

REPRODUCTIVE HISTORY

Women only:

How many <u>successful</u> pregnancies have you had? ⎵⎵

How many <u>unsuccessful</u> pregnancies have you had? ⎵⎵

At what age did you start menstruation? ⎵⎵ *years old*

At what age did you stop menstruation? ⎵⎵ *years old* Have not stopped ☐

SECTION 8: SOCIAL SUPPORT (to be completed by the patient)

Here is a list of some things that other people do for us or give us that may be helpful or supportive. Please read each statement carefully and put a tick the box that is closest to your situation.

Example Example Example Example Example Example

	As much as I would like				Much less than I would like
I get enough vacation time……………………………	☐	☑	☐	☐	☐

If you put a check where we have, it means that you get *almost* as much vacation time as you would like, but not quite as much as you would like.

		As much as I would like				Much less than I would like
8.1.	I have people who care what happens to me…….	☐	☐	☐	☐	☐
8.2.	I get love and affection……………………………..	☐	☐	☐	☐	☐
8.3.	I get chance to talk to someone I trust about problems at work or with my housework ………..	☐	☐	☐	☐	☐
8.4.	I get chance to talk to someone I trust about my personal and family problems……………………..	☐	☐	☐	☐	☐
8.5.	I get chance to talk about money matters ……….	☐	☐	☐	☐	☐
8.6.	I get invitations to go out and do things with other people ………………………………………………..	☐	☐	☐	☐	☐
8.7.	I get useful advice about important things in life ...	☐	☐	☐	☐	☐
8.8.	I get help when I am sick in bed …………………..	☐	☐	☐	☐	☐

SECTION 9: GENERAL HEALTH (to be completed by the patient)

We are interested in some things about you and your health. Please answer all of the questions yourself by ticking the box that best applies to you. There are no “right” or “wrong” answers. The answers that you provide will remain strictly confidential.

	Not at all	A little	Quite a bit	Very much
9.1. Do you have any trouble doing strenuous activities, like carrying a heavy shopping bag or a suitcase?......	1	2	3	4
9.2. Do you have any trouble taking a long walk?...........	1	2	3	4
9.3. Do you have any trouble taking a short walk outside of the house?..	1	2	3	4
9.4. Do you need to stay in bed or a chair during the day?...	1	2	3	4
9.5. Do you need help with eating, dressing, washing yourself or using the toilet?...	1	2	3	4

**During the past week:**	Not at all	A little	Quite a bit	Very much
9.6. Were you limited in doing either your work or other daily activities?...	1	2	3	4
9.7. Were you limited in pursuing your hobbies or other leisure time activities?..	1	2	3	4
9.8. Were you short of breath?..	1	2	3	4
9.9. Have you had pain?...	1	2	3	4
9.10. Did you need to rest?..	1	2	3	4
9.11. Have you had trouble sleeping?.......................	1	2	3	4
9.12. Have you felt weak?.......................................	1	2	3	4
9.13. Have you lacked appetite?...............................	1	2	3	4

GENERAL HEALTH *(Continued)*

**During the past week:**	**Not at all**	**A little**	**Quite a bit**	**Very much**
9.14. Have you felt nauseated?...	1	2	3	4
9.15. Have you vomited?...	1	2	3	4
9.16. Have you been constipated?....................................	1	2	3	4
9.17. Have you had diarrhea?..	1	2	3	4
9.18. Were you tired?...	1	2	3	4
9.19. Did pain interfere with your daily activities?...............	1	2	3	4
9.20. Have you had difficulty in concentrating on things, like reading a newspaper or watching television?..........	1	2	3	4
9.21. Did you feel tense?...	1	2	3	4
9.22. Did you worry?..	1	2	3	4
9.23. Did you feel irritable?..	1	2	3	4
9.24. Did you feel depressed?...	1	2	3	4
9.25. Have you had difficulty remembering things?............	1	2	3	4
9.26. Has your physical condition or medical treatment interfered with your <u>family</u> life?......................................	1	2	3	4
9.27. Has your physical condition or medical treatment interfered with your <u>social</u> life?......................................	1	2	3	4
9.28. Has your physical condition or medical treatment caused you financial difficulties?....................................	1	2	3	4

For the following questions, please circle the number between 1 and 7 that best applies to you

9.29. How would you rate your overall health during the past week?

1 2 3 4 5 6 7

Very Poor ………………………………………………………………………………………….. Excellent

9.30. How would you rate your overall quality of life during the past week?

1 2 3 4 5 6 7

Very Poor ………………………………………………………………………………………….. Excellent

TO BE PRINTED ON HOSPITAL LETTERHEADED PAPER.

<Patient Title> <Patient Surname>
<Patient Address 1>
<Patient Address 2>
<Patient Address 3>
<Patient Address 4>
<Patient Address 5>
<Patient Post Code>

<Date>

Dear <Patient Title> <Patient Surname>

RE: Bladder Cancer Prognosis Programme – postal information request

We would like to thank you once again for taking part in the Bladder Cancer Prognosis Programme.

As explained to you by your research nurse, we would like you to complete a questionnaire and a diary at home. Completing them at home allows you time to gather the information requested and if necessary to ask other family members or friends for their help to fill it in.

Enclosed in this pack are a **dairy** and a **questionnaire**. Each of these documents has full instructions to tell you how to fill them in. Please take time to read the instructions carefully and contact your research nurse on the telephone number below if you have any queries.

The Diary is to be used to collect information on what you eat and drink during the course of a normal week and also the number of times that you pass water during the course of 3 days. Please start completing the diary as soon as possible after receiving this letter (ideally this will be the next day). It is important that you allow at least 1 week to fill it in before you come back to hospital for your follow-up appointment.

The questionnaire asks about your employment and residential history and your family's history of cancer.

Please bring both the **diary** and the **questionnaire** back to the research nurse at the hospital when you are seen at your **next follow-up appointment on <Date of Appointment>**. Your research nurse will go through the questionnaire and diary with you on your return and will be happy to answer any questions.

This information is very important to us and will help us to look into some of the lifestyle factors that can influence the way in which bladder cancers behave.

Thank you again for your continued participation.

Yours sincerely,

<Urologist>

Study Number: ☐☐☐☐

BLADDER CANCER PROGNOSIS PROGRAMME

Cancer Research UK Bladder Cancer Group

Postal Questionnaire

CONFIDENTIAL

VERSION: FINAL 1.0 November 2005

PLEASE DESTROY PREVIOUS VERSIONS

MREC APPROVAL: 23rd November 2005

START DATE: 1st January 2006

A programme of research

Conducted by: UNIVERSITY OF BIRMINGHAM

Funded by: CANCER RESEARCH UK

INTRODUCTION

As part of the Bladder Cancer Prognosis Programme, we would like to collect information about where you have lived and which types of jobs you have had. We would also like you to tell us about the members of your immediate family and whether or not they have ever had cancer. There are **three** sections in this questionnaire.

- Section 1: Occupational History
- Section 2: Residential History
- Section 3: Family History of Cancer

We would like you to go through each section at a time, reading the instructions at the beginning of each section and looking at the examples that we have provided which, will help you to know how to fill in the questionnaire.

The questions in section 3 ask for personal family information. If you feel that any of the questions are too personal, do not answer them. The questions are not used to test you in any way and the responses that you give will not be used to make any judgements of you. There are no right or wrong answers. All of your responses will be treated as strictly confidential and will be used only for medical research.

You may find that some of the information we are asking you for may not be available (e.g. some postcodes may not exist for some of your past addresses). Do not worry, please remember to provide us with as much detail as possible. Try to answer every question even if the answer is ‘I can’t remember’ or ‘I don’t know’.

Please bring the completed questionnaire with you to your next hospital follow-up appointment and give it to the research nurse.

If you have any questions or if there is anything that you feel you don’t understand, please telephone your research nurse at any time on <research nurse mobile>.

Thank you again for taking the time to participate in this study.

SECTION ONE: OCCUPATIONAL HISTORY

In this section we would like you to tell us about your **most recent job** and all of your **previous jobs** that you have had for one year or more

Here are 3 examples of how to fill in this section of the questionnaire

Most Recent Job # ***Example Example Example Example Example Example***

Time period that you worked there: From year: 1972 To year: 1996

Job Title:	DRIVER
Description of Activities:	HGV DRIVER
Name of Organisation:	JO BLOGGS BUILDERS
Location (town/city):	BIRMINGHAM
Type of Business:	BUILDING CONTRACTORS

Previous Job # 1 ***Example Example Example Example Example Example***

Time period that you worked there: From year: 1972 To year: 1986

Job Title:	OFFICE CLERK
Description of Activities:	RECEPTION / OFFICE DUTIES
Name of Organisation:	JO BLOGGS ACCOUNTANTS
Location (town/city):	DUDLEY
Type of Business:	ACCOUNTANCY

Previous Job # 2 ***Example Example Example Example Example Example***

Time period that you worked there: From year: 1968 To year: 1972

Job Title:	TEACHER
Description of Activities:	TEACHING ENGLISH
Name of Organisation:	JO BLOGGS COMPREHENSIVE SCHOOL
Location (town/city):	CARDIFF
Type of Business:	

Please continue on to the next page:

OCCUPATIONAL HISTORY: *(continued)*

Using **CAPITAL LETTERS** and completing **ONE** section for each job, please list your **most recent job first**, followed by all of your **previous jobs.**

We have allowed enough sections for **up to 5** jobs. If you have had more than 5 jobs, complete all 5 sections of this part of the questionnaire and then continue on a **separate piece of paper**.

Current or Most Recent Job

Time period that you worked there: From year: ☐☐☐☐ To year: ☐☐☐☐

Job Title:

Description of Activities:

Name of Organisation:

Location (town/city):

Type of Business:

Previous Job # 1

Time period that you worked there: From year: ☐☐☐☐ To year: ☐☐☐☐

Job Title:

Description of Activities:

Name of Organisation:

Location (town/city):

Type of Business:

Previous Job # 2

Time period that you worked there: From year: ☐☐☐☐ To year: ☐☐☐☐

Job Title:

Description of Activities:

Name of Organisation:

Location (town/city):

Type of Business:

Please continue on to the next page:

OCCUPATIONAL HISTORY: *(continued)*

Previous Job # 3

Time period that you worked there: From year: [| | |] To year: [| | |]

Job Title:

Description of Activities:

Name of Organisation:

Location (town/city):

Type of Business:

Previous Job # 4

Time period that you worked there: From year: [| | |] To year: [| | |]

Job Title:

Description of Activities:

Name of Organisation:

Location (town/city):

Type of Business:

Previous Job # 5

Time period that you worked there: From year: [| | |] To year: [| | |]

Job Title:

Description of Activities:

Name of Organisation:

Location (town/city):

Type of Business:

Please continue on a separate piece of paper if needed:

SECTION TWO: RESIDENTIAL HISTORY

In this section we would like you to tell us about **your previous addresses**. We only need you to tell us about previous addresses at which you have lived for a period of more than **ONE YEAR** during your life. You do not need to tell us your current address.

Here are **3 examples** of how to fill in this section of the questionnaire

Previous Address # 1: ***Example Example Example Example Example Example***

Time period that you lived there: From year: 1951 To year: 1968

House Number & Street:	220
	HAGLEY ROAD
Location (town/city):	BIRMINGHAM
County:	WEST MIDLANDS
Country:	ENGLAND
Postcode (if known):	B15 IMM

Previous Address # 2: ***Example Example Example Example Example Example***

Time period that you lived there: From year: 1968 To year: 1972

House Number & Street:	350
	NETAJI SUBHAS ROAD
Location (town/city):	CALCUTTER
County:	WEST BENGAL
Country:	INDIA
Postcode (if known):	

Previous Address # 3: ***Example Example Example Example Example Example***

Time period that you lived there: From year: 1972 To year: 1986

House Number & Street:	FLAT 1A, HIGHFIELD TOWER
	7 CHAPEL ASH LANE
Location (town/city):	WOLVERHAMPTON
County:	WEST MIDLANDS
Country:	ENGLAND
Postcode (if known):	WV10 4HT

Please continue on to the next page:

RESIDENTIAL HISTORY: *(continued)*

Using **CAPITAL LETTERS** and completing **ONE** section for each address, please list your all of your **PREVIOUS ADDRESSES** that you have lived at for **more than a year. We do not need you to list your current address.**

We have allowed enough sections for **up to 6** addresses. If you have had more than 6 addresses, complete all 6 sections of this part of the questionnaire and then continue on a **separate piece of paper**.

PREVIOUS ADDRESS # 1

Time period that you lived there: From year: ☐☐☐☐ To year: ☐☐☐☐

House Number & Street:

Location (town/city):

County:

Country:

Postcode (if known):

PREVIOUS ADDRESS # 2

Time period that you lived there: From year: ☐☐☐☐ To year: ☐☐☐☐

House Number & Street:

Location (town/city):

County:

Country:

Postcode (if known):

PREVIOUS ADDRESS # 3

Time period that you lived there: From year: ☐☐☐☐ To year: ☐☐☐☐

House Number & Street:

Location (town/city):

County:

Country:

Postcode (if known):

Please continue onto the next page:

RESIDENTIAL HISTORY: *(continued)*

PREVIOUS ADDRESS # 4

Time period that you lived there: From year: To year:

House Number & Street:

Location (town/city):

County:

Country:

Postcode (if known):

PREVIOUS ADDRESS # 5

Time period that you lived there: From year: To year:

House Number & Street:

Location (town/city):

County:

Country:

Postcode (if known):

PREVIOUS ADDRESS # 6

Time period that you lived there: From year: To year:

House Number & Street:

Location (town/city):

County:

Country:

Postcode (if known):

Please continue on a separate piece of paper if needed:

SECTION THREE: FAMILY HISTORY OF CANCER

In the following sections we are asking for information on **ALL** members of your **immediate biological family** (i.e. blood relations) and their **medical history** of **cancer.**

You **DO NOT** need to tell us about any **adopted** or **step-relations.** Please tell us whether your **immediate family members** have had **cancer**, which **type of cancer** and their **age** at which the **cancer was diagnosed (**if known).

Here are 6 examples of how to complete this section of the questionnaire:

Example Example Example Example Example Example Example Example

Relation: FATHER

Year of Birth: 1 | 9 | 1 | 1

Have they ever had cancer: Yes ☑ No ☐ Don't know ☐

If Yes, Type of cancer: LUNG

Age at which cancer was diagnosed(if known): 7 | 2 Years old

Example Example Example Example Example Example Example Example

Relation: MOTHER

Year of Birth: 1 | 9 | 1 | 5

Have they ever had cancer: Yes ☐ No ☑ Don't know ☐

If Yes, Type of cancer:

Age at which cancer was diagnosed(if known): | Years old

Example Example Example Example Example Example Example Example

Relation: BROTHER ☑ SISTER ☐

Year of Birth: 1 | 9 | 5 | 1

Have they ever had cancer: Yes ☐ No ☑ Don't know ☐

If Yes, Type of cancer:

Age at which cancer was diagnosed(if known): | Years old

Please continue on to the next page:

SECTION THREE: *(continued)*

Here are some more examples of how to complete Section 3 of the questionnaire:

Example Example Example Example Example Example Example Example

Relation: BROTHER ☐ SISTER ☑

Year of Birth: 1 9 3 9

Have they ever had cancer: Yes ☑ No ☐ Don't know ☐

If Yes, Type of cancer: OVARIAN CANCER

Age at which cancer was diagnosed(if known): 4 5 Years old

Example Example Example Example Example Example Example Example

Relation: SON ☐ DAUGHTER ☑

Year of Birth: 1 9 6 8

Have they ever had cancer: Yes ☐ No ☑ Don't know ☐

If Yes, Type of cancer:

Age at which cancer was diagnosed(if known): Years old

Example Example Example Example Example Example Example Example

Relation: SON ☐ DAUGHTER ☑

Year of Birth: 1 9 7 2

Have they ever had cancer: Yes ☑ No ☐ Don't know ☐

If Yes, Type of cancer: BREAST CANCER

Age at which cancer was diagnosed(if known): Years old

Please continue on to the next page:

SECTION THREE: *(continued)*

In this section we would like you to tell us about your **PARENTS**. Please fill in the table below using **CAPITAL LETTERS** and **completing ONE section** for **EACH parent.**

Relation: **FATHER**

Year of Birth: ☐☐☐☐

Have they ever had cancer: Yes ☐ No ☐ Don't know ☐

If Yes, Type of cancer: ____________________

Age at which cancer was diagnosed(if known): ☐☐ Years old

Relation: **MOTHER**

Year of Birth: ☐☐☐☐

Have they ever had cancer: Yes ☐ No ☐ Don't know ☐

If Yes, Type of cancer: ____________________

Age at which cancer was diagnosed(if known): ☐☐ Years old

Please continue on to the next page:

SECTION THREE: *(continued)*

In this section we would like you to tell us how many **siblings** you have in your family. We would also like to ask you about your **brothers** and **sisters** medical history of cancer. You **DO NOT** need to tell us about any **adopted** or **step-relations.**

Please fill in the table below using **CAPITAL LETTERS**. Complete **ONE section** for **EACH sibling** by **ticking** the appropriate **box** to **indicate brother** or **sister.**

We have allowed enough sections for **up to 6** siblings. If you have more than 6 siblings, complete all 6 sections of this part of the questionnaire and then continue on a **separate piece of paper**.

HOW MANY SIBLINGS DO YOU HAVE: BROTHERS ☐☐ **SISTERS** ☐☐

Sibling # 1

Relation: Brother ☐ Sister ☐

Year of Birth: ☐☐☐☐

Have they ever had cancer: Yes ☐ No ☐ Don't know ☐

If Yes, Type of cancer: ________

Age at which cancer was diagnosed(if known): ☐☐ Years old

Sibling # 2

Relation: Brother ☐ Sister ☐

Year of Birth: ☐☐☐☐

Have they ever had cancer: Yes ☐ No ☐ Don't know ☐

If Yes, Type of cancer: ________

Age at which cancer was diagnosed(if known): ☐☐ Years old

Please continue on to the next page:

SECTION THREE *(continued)*

Sibling # 3

Relation: Brother ☐ Sister ☐

Year of Birth: ☐☐☐☐

Have they ever had cancer: Yes ☐ No ☐ Don't know ☐

If Yes, Type of cancer: ________

Age at which cancer was diagnosed(if known): ☐☐ Years old

Sibling # 4

Relation: Brother ☐ Sister ☐

Year of Birth: ☐☐☐☐

Have they ever had cancer: Yes ☐ No ☐ Don't know ☐

If Yes, Type of cancer: ________

Age at which cancer was diagnosed(if known): ☐☐ Years old

Sibling # 5

Relation: Brother ☐ Sister ☐

Year of Birth: ☐☐☐☐

Have they ever had cancer: Yes ☐ No ☐ Don't know ☐

If Yes, Type of cancer: ________

Age at which cancer was diagnosed(if known): ☐☐ Years old

Sibling # 6

Relation: Brother ☐ Sister ☐

Year of Birth: ☐☐☐☐

Have they ever had cancer: Yes ☐ No ☐ Don't know ☐

If Yes, Type of cancer: ________

Age at which cancer was diagnosed(if known): ☐☐ Years old

Please continue on a separate piece of paper if needed:

SECTION THREE *(continued)*

In this section we would like you to tell us **how** many children you have had. We would also like you to tell us about all of your children's (i.e. blood relations) **medical history of cancer.** You **DO NOT** need to tell us about any **adopted** or **step-relations.**

Please fill in the table below using **CAPITAL LETTERS**. Then complete **ONE** section for EACH child, ticking the appropriate box to indicate son or daughter.

We have allowed enough sections for **up to 6** children. If you have more than 6 children, complete all 6 sections of this part of the questionnaire and then continue on a **separate piece of paper**.

HOW MANY CHILDREN HAVE YOU HAD? SONS ☐☐ **DAUGHTERS** ☐☐

Child # 1

Relation: Son ☐ Daughter ☐

Year of Birth: ☐☐☐☐

Have they ever had cancer: Yes ☐ No ☐ Don't know ☐

If Yes, Type of cancer: ____________

Age at which cancer was diagnosed(if known): ☐☐ Years old

Child # 2

Relation: Son ☐ Daughter ☐

Year of Birth: ☐☐☐☐

Have they ever had cancer: Yes ☐ No ☐ Don't know ☐

If Yes, Type of cancer: ____________

Age at which cancer was diagnosed(if known): ☐☐ Years old

Please continue on to the next page:

SECTION THREE: *(continued)*

Child # 3

Relation: Son ☐ Daughter ☐

Year of Birth: ☐☐☐☐

Have they ever had cancer: Yes ☐ No ☐ Don't know ☐

If Yes, Type of cancer: ____________

Age at which cancer was diagnosed(if known): ☐☐ Years old

Child # 4

Relation: Son ☐ Daughter ☐

Year of Birth: ☐☐☐☐

Have they ever had cancer: Yes ☐ No ☐ Don't know ☐

If Yes, Type of cancer: ____________

Age at which cancer was diagnosed(if known): ☐☐ Years old

Child # 5

Relation: Son ☐ Daughter ☐

Year of Birth: ☐☐☐☐

Have they ever had cancer: Yes ☐ No ☐ Don't know ☐

If Yes, Type of cancer: ____________

Age at which cancer was diagnosed(if known): ☐☐ Years old

Child # 6

Relation: Son ☐ Daughter ☐

Year of Birth: ☐☐☐☐

Have they ever had cancer: Yes ☐ No ☐ Don't know ☐

If Yes, Type of cancer: ____________

Age at which cancer was diagnosed(if known): ☐☐ Years old

Please continue on a separate piece of paper if needed

Study Number:

BLADDER CANCER PROGNOSIS PROGRAMME

Cancer Research UK Bladder Cancer Group

7-Day Lifestyle Diary

CONFIDENTIAL

VERSION: FINAL 1.0 November 2005
PLEASE DESTROY PREVIOUS VERSIONS

MREC APPROVAL: 23rd November 2005
START DATE: 1st January 2006

A programme of research

Conducted by: UNIVERSITY OF BIRMINGHAM

Funded by: CANCER RESEARCH UK

INTRODUCTION

This Diary is to be used to collect information on what you eat and drink during the course of a normal week and also the number of times that you pass water during the course of 3 days. Please start completing the diary as soon as possible after receiving it (ideally this will be the next day). It is important that you allow at least 1 week to fill it in before you come back to hospital for your follow-up appointment.

The first section of this diary is to be used to record the number of times you visit the toilet to pass water, during the course of 3 days. There is a page to complete for each of the 3 days (pages 2-4) At the top of each page you should write in the date and the day of the week. You should then write the number of times during each hour of the day you have passed water. For example. If you visited the toilet to pass water twice between 9 and 10 am, you would write ‘2’ in the box provided. You don’t need to write anything in the boxes next to the hours that you did not visit the toilet.

The second section of this diary is to be used to record what you eat and drink over a period of 7 days. You should start filling this in at the same time as the first section. There are several pages of guidance notes and examples on how to complete the food diary. Please take time to read them carefully and to follow the instructions.

Please bring the completed questionnaire with you to your next hospital follow-up appointment and give it to the research nurse.

If you have any questions or if there is anything that you feel you don’t understand, please telephone your research nurse at any time on <research nurse mobile>.

Thank you again for taking the time to participate in this study.

3 Day Micturition Diary

As part of the study we are interested in how often you pass water. We would like you to keep a diary for 3 days recording how frequent you visit the toilet to pass water.

Day No. 1

DATE ☐☐☐☐ 2 0 ☐☐ DAY OF WEEK ☐

Time of Day	Number of times you passed water
Between 12 midnight –1am…………	☐☐ times
Between 1am –2am…………………..	☐☐ times
Between 2am-3am……………………	☐☐ times
Between 3am-4am……………………	☐☐ times
Between 4am-5am……………………	☐☐ times
Between 5am-6am……………………	☐☐ times
Between 6am-7am……………………	☐☐ times
Between 7am-8am……………………	☐☐ times
Between 8am-9am……………………	☐☐ times
Between 9am-10am…………………..	☐☐ times
Between 10am-11am…………………	☐☐ times
Between 11am and 12 Noon………..	☐☐ times
Between 12 Noon –1pm……………..	☐☐ times
Between 1pm –2pm…………………..	☐☐ times
Between 2pm-3pm……………………	☐☐ times
Between 3pm-4pm……………………	☐☐ times
Between 4pm-5pm……………………	☐☐ times
Between 5pm-6pm……………………	☐☐ times
Between 6pm-7pm……………………	☐☐ times
Between 7pm-8pm……………………	☐☐ times
Between 8pm-9pm……………………	☐☐ times
Between 9pm-10pm…………………..	☐☐ times
Between 10pm-11pm…………………	☐☐ times
Between 11pm and 12 Midnight…….	☐☐ times

Day No. 2

DATE ☐☐ ☐☐ 2 0 ☐☐ DAY OF WEEK ☐

Time of Day	Number of times you passed water
Between 12 midnight –1am…………	☐☐ times
Between 1am –2am…………………..	☐☐ times
Between 2am-3am……………………	☐☐ times
Between 3am-4am……………………	☐☐ times
Between 4am-5am……………………	☐☐ times
Between 5am-6am……………………	☐☐ times
Between 6am-7am……………………	☐☐ times
Between 7am-8am……………………	☐☐ times
Between 8am-9am……………………	☐☐ times
Between 9am-10am…………………..	☐☐ times
Between 10am-11am…………………	☐☐ times
Between 11am and 12 Noon………..	☐☐ times
Between 12 Noon –1pm……………..	☐☐ times
Between 1pm –2pm…………………..	☐☐ times
Between 2pm-3pm……………………	☐☐ times
Between 3pm-4pm……………………	☐☐ times
Between 4pm-5pm……………………	☐☐ times
Between 5pm-6pm……………………	☐☐ times
Between 6pm-7pm……………………	☐☐ times
Between 7pm-8pm……………………	☐☐ times
Between 8pm-9pm……………………	☐☐ times
Between 9pm-10pm…………………..	☐☐ times
Between 10pm-11pm…………………	☐☐ times
Between 11pm and 12 Midnight…….	☐☐ times

Day No. 3

DATE ☐☐ ☐☐ 2 0 ☐☐ DAY OF WEEK ______

Time of Day	Number of times you passed water
Between 12 midnight –1am…………	☐☐ times
Between 1am –2am…………………..	☐☐ times
Between 2am-3am……………………	☐☐ times
Between 3am-4am……………………	☐☐ times
Between 4am-5am……………………	☐☐ times
Between 5am-6am……………………	☐☐ times
Between 6am-7am……………………	☐☐ times
Between 7am-8am……………………	☐☐ times
Between 8am-9am……………………	☐☐ times
Between 9am-10am…………………..	☐☐ times
Between 10am-11am…………………	☐☐ times
Between 11am and 12 Noon………..	☐☐ times
Between 12 Noon –1pm………………	☐☐ times
Between 1pm –2pm…………………..	☐☐ times
Between 2pm-3pm……………………	☐☐ times
Between 3pm-4pm……………………	☐☐ times
Between 4pm-5pm……………………	☐☐ times
Between 5pm-6pm……………………	☐☐ times
Between 6pm-7pm……………………	☐☐ times
Between 7pm-8pm……………………	☐☐ times
Between 8pm-9pm……………………	☐☐ times
Between 9pm-10pm…………………..	☐☐ times
Between 10pm-11pm…………………	☐☐ times
Between 11pm and 12 Midnight…….	☐☐ times

FOOD DIARY

We would like you to keep this diary of everything you eat and drink over the next seven days.

It is very important that you do not adjust what you eat and drink just because you are keeping a record. Please continue to eat whatever you wish.

Instructions

As you will see, each day is marked in sections, beginning with the first thing in the morning and ending with bedtime. For each part of the day write down all food and drink consumed, the amounts, and description if necessary. If nothing is eaten or drunk during a part of the day, draw a line through that section. Record everything at the time of eating, not from memory at the end of the day.

On the next eight pages is a list of popular foods and drinks. Next to each item is the sort of thing we need to know so that we can tell what it is made of and how much you had. This list cannot cover all the foods and drinks, so try to relate to a similar item if any that you have eaten are missing. Please give as much detail as you can. There is an example on page 17.

For some foods you may find it easier to describe how much you had by comparing it to one of the photographs on pages 13 to 17.

Many packet foods have weights printed on them, so please use these to show how much you ate.

At the end of each day there is a list of snacks and drinks that can easily be forgotten. Please write any extra items in here if you have not already recorded them in some other part of the day.

FOR EACH ITEM THAT YOU EAT OR DRINK PLEASE READ THE FOLLOWING FOR DETAILS REQUIRED:

Always state what sort of **oil** or **fat** was used for baking, frying etc.

Give **brand and full name** of products where possible.

For **meals/snacks eaten away from home**, please note where these items were eaten, giving name and/or type of restaurant, café, pub etc., where appropriate.

Please could you answer the questions at the back (pages 53-56) **after** you have completed the seven days of the diary.

Please remember to provide us with as much detail as you possibly can.

**WHERE POSSIBLE, ALWAYS STATE WHAT SORT OF OIL OR FAT WAS USED FOR BAKING, FRYING ETC.**		
**Food/Drink**	**Description & Preparation**	**Amount**
Home-made dishes	Please say what the dish is called and give recipe or ingredients, including amounts, if possible	tablespoons, or one of the pictures
Ready-made meals	What sort: e.g. pizzas, microwave dishes, slimmers' meals etc. Please give main ingredients and nutrition information on packet, and enclose label or bar code if possible.	Weight from packet
Meals eaten away from home	What sort: e.g. pizzas, Chinese, Indian dishes, fish and chips, hamburgers, hot dogs etc. Please say what the dish is called and give ingredients where possible. Give name of the restaurant if it is a well known chain.	Tablespoons, number or one of the pictures

**WHERE POSSIBLE, ALWAYS STATE WHAT SORT OF OIL OR FAT WAS USED FOR BAKING, FRYING ETC.**		
**Food/Drink**	**Description & Preparation**	**Amount**
Bacon	Lean or streaky; fried or grilled rashers	Number
Baked beans	Standard or reduced sugar/salt	Tablespoons, tin size or picture 12
Beefburger (hamburger)	Home-made, from a packet or take away; fried, microwaved or grilled; well done, rare etc; large or small; with or without bread roll	Number
Beer	Stout, bitter, lager, keg, draught, bottled, canned, low alcohol, strong, home-made; give brand if possible; % alcohol	Number of pints/half pints, cans/bottles, including size
Biscuits	Plain; savoury; cheese, crispbread, sweet, chocolate, wafer, home-made; size; include biscuits like Kit-Kat and Penguin; write in the name and brand if you can	Number
Bread (see also sandwiches)	Wholemeal, white or brown; currant, fruit, malt; large or small loaf; thick, medium or thin slices; sliced or unsliced; give brand if possible	Number of slices
Bread rolls	Wholemeal, white or brown; alone or with filling (see sandwiches); size; crusty or soft; give brand if possible	Number of rolls
Breakfast cereal, bran, wheatgerm	What sort; cornflakes, Weetabix, muesli etc.; give brand if possible	Number of biscuits, tablespoons or picture 1
Bun	What sort; iced currant; sweet or plain; large or small; give name and brand if possible	Number
Butter for bread	Ordinary or low fat dairy spread, write in the name and brand if you can	Thick, average, thin spread

WHERE POSSIBLE, ALWAYS STATE WHAT SORT OF OIL OR FAT WAS USED FOR BAKING, FRYING ETC.		
**Food/Drink**	**Description & Preparation**	**Amount**
Cake-small	What sort; cream, iced; sort of filling; give name and brand if possible	Number
Cake-large	What sort; cream, iced; sort of filling; give name and brand if possible	Slices or pictures 15 or 16
Cheese	What sort; cream, cottage, hard, soft; low fat; write in the name if you can	Tablespoons or picture 2
Chips	Fresh, frozen, oven, microwave or crinkle cut; type of fat for cooking; give brand if possible	Picture 7
Chocolate	What sort; plain, milk, white, diabetic; give name and brand if possible	Number or bar weight
Chops	What sort; lean or fatty; large or small; fried, grilled, baked etc; well done, rare etc.	Number
Cider	Sweet, dry, vintage, low alcohol; % alcohol	Pints and half pints; number of cans/bottles; including size
Coffee	With/without milk, what sort, half milk/half water; all milk, ground, instant, decaffeinated/caffeinated; strong, average or weak	Cups or mugs
Condiments	Pepper, salt or substitute	½ or ¼ teaspoon, pinch etc.
Cooking oil	Type; brand name	Teaspoons
Cream	Half, single, sour, whipping, double, clotted; low fat; fresh or substitute; sweetened or unsweetened	Tablespoons
Crisps	Brand name; low fat; low salt	Packet weight
Egg	How was it cooked; boiled, fried, scrambled, poached, omelette, etc.	Number

WHERE POSSIBLE, ALWAYS STATE WHAT SORT OF OIL OR FAT WAS USED FOR BAKING, FRYING ETC.		
**Food/Drink**	**Description & Preparation**	**Amount**
Fish	What sort; fried, boiled, grilled, poached, microwaved; pickled, smoked or salted; with batter or breadcrumbs; tinned with oil or tomato sauce; size; give brand name where applicable	Helping, number or picture 6
Fish cakes or finger	What sort; large, medium or small size; fried or grilled; give brand name if possible	Number
Fruit-fresh	What sort and variety e.g. Cox apple; cored; with or without skin	Number
Fruit-stewed or canned	What sort and variety e.g. Bramley apple; with or without sugar; in fruit juice or syrup	Tablespoons or in tin size
Fruit-juice	What sort; sweetened or unsweetened	Glasses or cups
Gravy	Thick or thin, instant or packet, made with or without dripping, meat juices etc.	Tablespoons
Herbs	Type, fresh or dried	½ or ¼ teaspoons
Honey, jam	Type, specify if low sugar	Teaspoons
Ice-cream	Dairy or non-dairy; flavour or variety	tablespoons
Liver, kidney	Pig, lamb, ox; fried or stewed	Picture 4 or 5
Margarine	Hard, soft, polyunsaturated, low fat, very low fat; give name and brand if possible	Thick, average or thin spread
Marmalade	Type and brand; specify if low sugar	Teaspoons; thick, average or thin spread
Mayonnaise	Give name and brand; state if low fat	Teaspoons
Meat pie, pastie, pastry	What sort; individual or helping; size; fat used for pastry; give brand name where possible	Number or picture 3

WHERE POSSIBLE, ALWAYS STATE WHAT SORT OF OIL OR FAT WAS USED FOR BAKING, FRYING ETC.		
**Food/Drink**	**Description & Preparation**	**Amount**
Meats	What sort; lean or fatty; fried, microwaved, grilled, roast, barbecued; well done or rare etc; with or without gravy, cut used; pickled, smoked or salted	Slices, helping or pictures 4 or 5
Milk-for drinking on its own or for cereals	Full cream, silver top, semi-skimmed, skimmed, sterilized, UHT, flavoured, powdered, soya	Pints, glasses or cups
Minced beef	On its own, with vegetables, fatty or lean	Tablespoons or picture 5
Peanuts	Dry roasted or ordinary salted	Packet weight
Porridge	With sugar or honey; with milk, cream or water	Bowls
Potatoes	Baked, boiled; with or without skin; mashed, creamed, fried/chips, instant, roast; with butter, margarine etc.	Tablespoons, or pictures 10 or 11
Pudding	What sort and brand; e.g. steamed sponge; with fruit; pie (what sort); jelly; blancmange; mousse; instant desserts; milk puddings, give recipe	Tablespoons, slices or pictures 3, 15 or 17
Rice	Brown or white; boiled or fried; rice pudding	Tablespoons or picture 8
Salad	Describe ingredients, with dressing; what sort of dressing (e.g. oil and vinegar, salad cream, mayonnaise)	Tablespoons or picture 14
Sandwiches and rolls	Wholemeal, white or brown bread; type of filling; butter or margarine; large or small loaf; thick, medium or thin slices	Number of rolls or slices of bread
Sauce-cold	What sort; e.g. tomato ketchup, brown sauce, soy sauce; salad cream; sweet or savoury	Tablespoons or picture 12

WHERE POSSIBLE, ALWAYS STATE WHAT SORT OF OIL OR FAT WAS USED FOR BAKING, FRYING ETC.		
**Food/Drink**	**Description & Preparation**	**Amount**
Sauce-hot	(for vegetables, meat or fish; puddings) what sort; savoury or sweet; thick or thin, give recipe if possible	Tablespoons or picture 12
Sausages	What sort; e.g. pork, beef, pork and beef; low fat; large or small; how cooked	Number
Sausage rolls	Large or small, type of pastry	Number
Scones	What sort; with currants, sweet or plain; cheese	Number
Snacks-in packet	What sort; e.g. cheese straws, Twiglets, pretzels (give brand name)	Packet weight or number
Soft drinks	Squash, undiluted or diluted; fizzy drinks; low calorie; give brand name	Glasses or cans
Soup	What sort; canned, packet, instant or vending machine, home-made; give brand name	Tablespoons; bowl or mug
Soya, Quorn	TVP, mince, burgers or tofu	Number or pictures 4 or 5
Spaghetti, other pasta	Canned, boiled; white, wholemeal; in sauce; give name and brand where applicable	Tablespoons or picture 9
Spices	Type	½ or ¼ teaspoons
Spreads	On bread, what sort of bread	½ or ¼ teaspoons; thick, average or thin spread
Spirits	What sort; e.g. whisky, gin, vodka, rum; at home or in pub	Single measures as in pub
Sugar	Added to cereals, tea, coffee, fruit etc; what sort; e.g. white, brown, Demerara	Heaped or level teaspoons

WHERE POSSIBLE, ALWAYS STATE WHAT SORT OF OIL OR FAT WAS USED FOR BAKING, FRYING ETC.		
**Food/Drink**	**Description & Preparation**	**Amount**
Sweets	What sort; e.g. toffees or boiled sweets; diabetic; give name and brand if possible	Number or packet size
Tea	With or without milk; what sort; herb, fruit, decaffeinated/caffeinated; tea bag or leaves; strong, average or weak	Cups or mugs
Vegetables	What sort and variety; with butter, other fat or sauce; fresh, frozen or canned; how cooked e.g. fried, boiled, microwaved or raw	Tablespoons or pictures 12, 13 or 14
Water	State whether tap, filtered, or bottled; give name and brand where applicable	Glasses or bottle size
Wine, sherry, port	White, red; sweet, medium, dry; low alcohol	Glasses or bottle size
Yoghurt, fromage frais	What sort; e.g. with fruit, natural, plain; flavour; low fat, Greek, creamy, soya; give full name and brand if possible	Carton weight/size or tablespoons

Use the pictures to help you to indicate the size of the portion you have eaten. Write down the picture number and size nearest to you own helping e.g. 2a, 3b, 1c etc.

The pictures could also be used for foods not shown e.g. fruit crumble might be a similar portion to shepherd's pie, fruit cake similar to veal and ham pie, and baked beans similar to peas.

Remember that the picture sizes are much smaller than life size. The arrow across the middle of the page indicates a dinner plate 10 inches in width.

1a

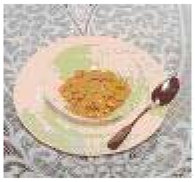

1b

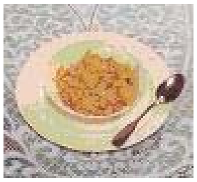

1c

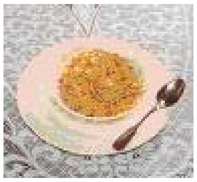

2a

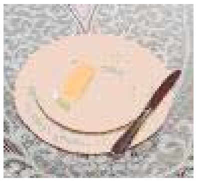

2b

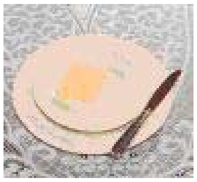

2c

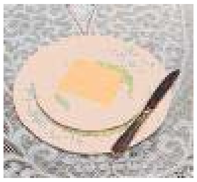

3a

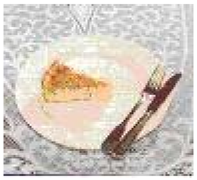

3b

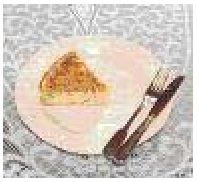

3c

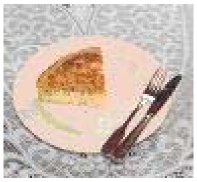

4a

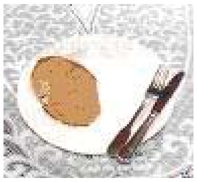

4b

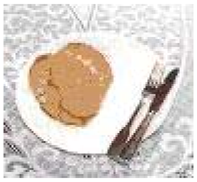

4c

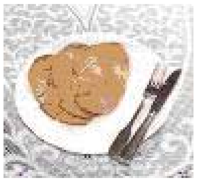

5a

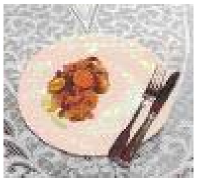

5b

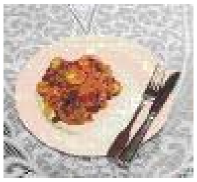

5c

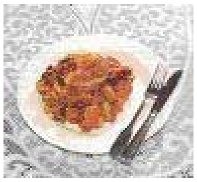

6a

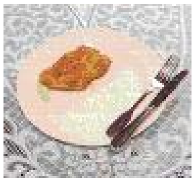

6b

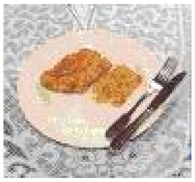

6c

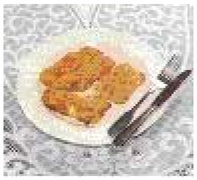

7a

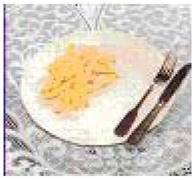

7b

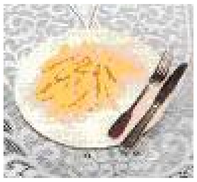

7c

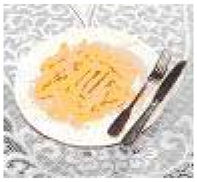

8a

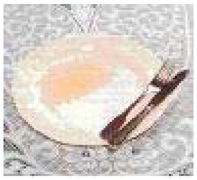

8b

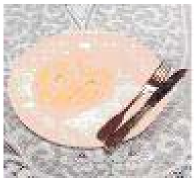

8c

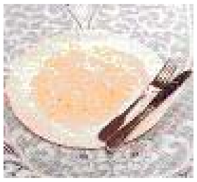

9a

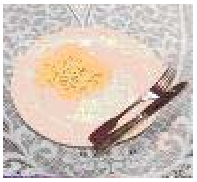

9b

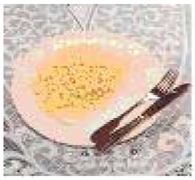

9c

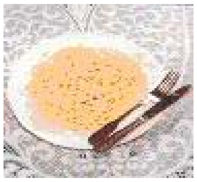

10a

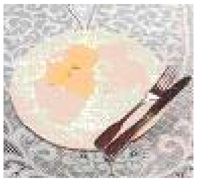

10b

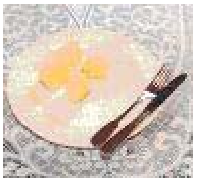

10c

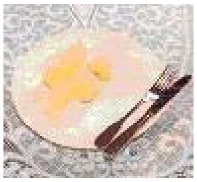

11a

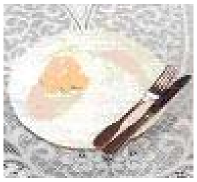

11b

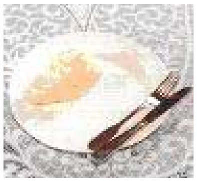

11c

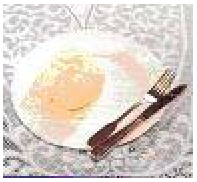

10" Plate

12a

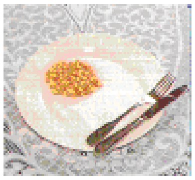

12b

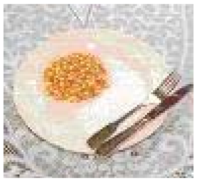

12c

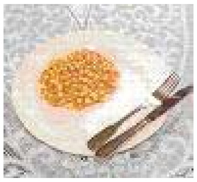

13a

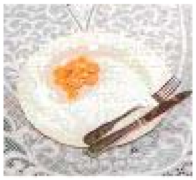

13b

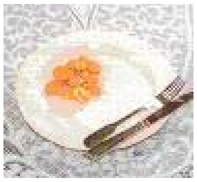

13c

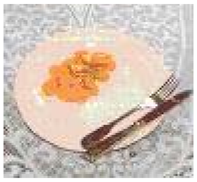

16a

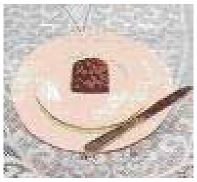

16b

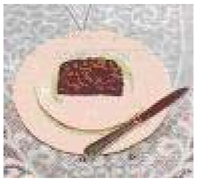

16c

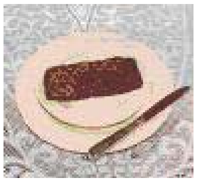

17a

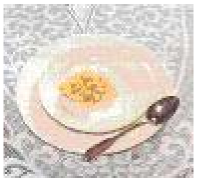

17b

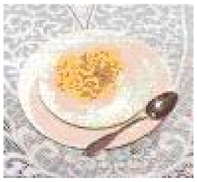

17c

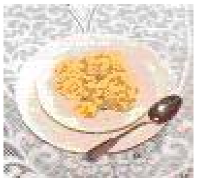

10" Plate

14a

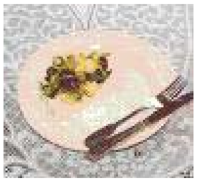

14b

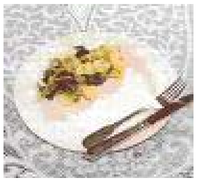

14c

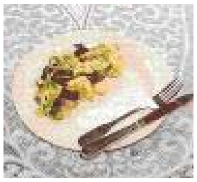

15a

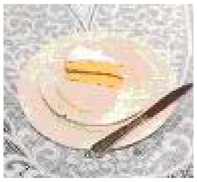

15b

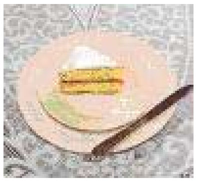

15c

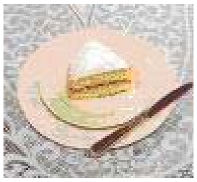

LUNCH

Food/Drink	Description and Preparation	Amount
Soup	*Campbell's condensed cream of mushroom, diluted half and half with water*	*1 medium bowl*
Bread	*White, large loaf Sunblest*	*2 medium slices*
Butter	*Anchor salted*	*Thick spread on each slice*
Biscuits	*Jacob's Cream Crackers*	*4*
Cheese	*Tesco matured cheddar*	*2a*
Tomatoes	*Fresh*	*2 medium*
Cake	*Home-made Victoria sponge with jam filling (see recipe)*	*15b*
Tea	Tetley's tea bag, weak	*2 cups*
Milk	Silver top, full cream	*1 tablespoon in each cup*
Sugar	*White granulated*	*2 heaped teaspoons in each*

EXAMPLE

Day No. 1

DATE [ ][ ] [ ][ ] [2][0][ ][ ] DAY OF WEEK [ ]

BEFORE BREAKFAST

Food/Drink	Description and Preparation	Amount

BREAKFAST

Food/Drink	Description and Preparation	Amount

MID MORNING- between breakfast time and lunch time

Food/Drink	Description and Preparation	Amount

LUNCH

Food/Drink	Description and Preparation	Amount

TEA		
**Food/Drink**	Description and Preparation	Amount
**EVENING MEAL**		
Food/Drink	Description and Preparation	Amount

LATER EVENING- up to last thing at night		
Food/Drink	Description and Preparation	Amount

BETWEEN MEALS, SNACKS AND DRINKS if not already written in before		
Food/Drink	Description and Preparation	Amount
Chocolate		
Toffees, sweets		
Crisps, peanuts		
Other snacks		
Beer, wine		
Sherry, Spirits		
Other cold drinks		
Tea, coffee		
Other hot drinks		
Ice cream		
Anything else?		

Space to write in the recipe or ingredients of any home-made dishes, take-away meals etc. that you have mentioned but not described previously. Where applicable, please list amounts of ingredients and brand names. For recipes, take-away meals etc., **please indicate amount/proportion actually consumed by yourself.**

END OF DAY No. 1

Day No. 2

DATE _ _ / _ _ / 2 0 _ _ **DAY OF WEEK** ____

BEFORE BREAKFAST

Food/Drink	Description and Preparation	Amount

BREAKFAST

Food/Drink	Description and Preparation	Amount

MID MORNING- between breakfast time and lunch time		
Food/Drink	Description and Preparation	Amount

LUNCH		
Food/Drink	Description and Preparation	Amount

TEA		
Food/Drink	Description and Preparation	Amount

EVENING MEAL		
Food/Drink	Description and Preparation	Amount

LATE EVENING- up to last thing at night		
Food/Drink	Description and Preparation	Amount

BETWEEN MEALS, SNACKS AND DRINKS if not already written in before		
Food/Drink	Description and Preparation	Amount
Chocolate		
Toffees, sweets		
Crisps, peanuts		
Other snacks		
Beer, wine		
Sherry, Spirits		
Other cold drinks		
Tea, coffee		
Other hot drinks		
Ice cream		
Anything else?		

Space to write in the recipe or ingredients of any home-made dishes, take-away meals etc. that you have mentioned but not described previously. Where applicable, please list amounts of ingredients and brand names. For recipes, take-away meals etc., **please indicate amount/proportion actually consumed by yourself.**
**END OF DAY No. 2**

Day No. 3

DATE ☐☐ ☐☐ 2 0 ☐☐ DAY OF WEEK

BEFORE BREAKFAST		
Food/Drink	Description and Preparation	Amount

BREAKFAST		
Food/Drink	Description and Preparation	Amount

MID MORNING- between breakfast time and lunch time		
Food/Drink	Description and Preparation	Amount

LUNCH		
Food/Drink	Description and Preparation	Amount

TEA		
Food/Drink	Description and Preparation	Amount
**EVENING MEAL**		
Food/Drink	Description and Preparation	Amount

LATER EVENING- up to last thing at night		
Food/Drink	Description and Preparation	Amount

BETWEEN MEALS, SNACKS AND DRINKS if not already written in before		
Food/Drink	Description and Preparation	Amount
Chocolate		
Toffees, sweets		
Crisps, peanuts		
Other snacks		
Beer, wine		
Sherry, Spirits		
Other cold drinks		
Tea, coffee		
Other hot drinks		
Ice cream		
Anything else?		

Space to write in the recipe or ingredients of any home-made dishes, take-away meals etc. that you have mentioned but not described previously. Where applicable, please list amounts of ingredients and brand names. For recipes, take-away meals etc., **please indicate amount/proportion actually consumed by yourself.**

END OF DAY No. 3

Day No. 4

DATE __ __ / __ __ / 2 0 __ __ **DAY OF WEEK** ________

BEFORE BREAKFAST

Food/Drink	Description and Preparation	Amount

BREAKFAST

Food/Drink	Description and Preparation	Amount

MID MORNING- between breakfast time and lunch time		
Food/Drink	Description and Preparation	Amount
**LUNCH**		
Food/Drink	Description and Preparation	Amount

TEA		
Food/Drink	Description and Preparation	Amount
**EVENING MEAL**		
Food/Drink	Description and Preparation	Amount

LATER EVENING- up to last thing at night		
Food/Drink	Description and Preparation	Amount

BETWEEN MEALS, SNACKS AND DRINKS if not already written in before		
Food/Drink	Description and Preparation	Amount
Chocolate		
Toffees, sweets		
Crisps, peanuts		
Other snacks		
Beer, wine		
Sherry, Spirits		
Other cold drinks		
Tea, coffee		
Other hot drinks		
Ice cream		
Anything else?		

Space to write in the recipe or ingredients of any home-made dishes, take-away meals etc. that you have mentioned but not described previously. Where applicable, please list amounts of ingredients and brand names. For recipes, take-away meals etc., **please indicate amount/proportion actually consumed by yourself.**

END OF DAY No. 4

Day No. 5		
DATE ☐☐ ☐☐ 2 0 ☐☐	DAY OF WEEK	
**BEFORE BREAKFAST**		
Food/Drink	Description and Preparation	Amount
**BREAKFAST**		
Food/Drink	Description and Preparation	Amount

**MID MORNING- between breakfast time and lunch time**		
Food/Drink	Description and Preparation	Amount
**LUNCH**		
Food/Drink	Description and Preparation	Amount

TEA		
Food/Drink	Description and Preparation	Amount
**EVENING MEAL**		
Food/Drink	Description and Preparation	Amount

LATER EVENING- up to last thing at night		
Food/Drink	Description and Preparation	Amount

BETWEEN MEALS, SNACKS AND DRINKS if not already written in before		
Food/Drink	Description and Preparation	Amount
Chocolate		
Toffees, sweets		
Crisps, peanuts		
Other snacks		
Beer, wine		
Sherry, Spirits		
Other cold drinks		
Tea, coffee		
Other hot drinks		
Ice cream		
Anything else?		

Space to write in the recipe or ingredients of any home-made dishes, take-away meals etc. that you have mentioned but not described previously. Where applicable, please list amounts of ingredients and brand names. For recipes, take-away meals etc., **please indicate amount/proportion actually consumed by yourself.**

END OF DAY No. 5

Day No. 6

DATE ☐☐ ☐☐ 20☐☐ **DAY OF WEEK** ________

BEFORE BREAKFAST

Food/Drink	Description and Preparation	Amount

BREAKFAST

Food/Drink	Description and Preparation	Amount

MID MORNING- between breakfast time and lunch time		
Food/Drink	Description and Preparation	Amount

LUNCH		
Food/Drink	Description and Preparation	Amount

TEA		
Food/Drink	Description and Preparation	Amount

EVENING MEAL		
Food/Drink	Description and Preparation	Amount

LATER EVENING- up to last thing at night		
Food/Drink	Description and Preparation	Amount

BETWEEN MEALS, SNACKS AND DRINKS if not already written in before		
Food/Drink	Description and Preparation	Amount
Chocolate		
Toffees, sweets		
Crisps, peanuts		
Other snacks		
Beer, wine		
Sherry, Spirits		
Other cold drinks		
Tea, coffee		
Other hot drinks		
Ice cream		
Anything else?		

Space to write in the recipe or ingredients of any home-made dishes, take-away meals etc. that you have mentioned but not described previously. Where applicable, please list amounts of ingredients and brand names. For recipes, take-away meals etc., **please indicate amount/proportion actually consumed by yourself.**

END OF DAY No. 6

Day No. 7		
DATE ☐☐ ☐☐ 2 0 ☐☐	DAY OF WEEK	
**BEFORE BREAKFAST**		
Food/Drink	Description and Preparation	Amount
**BREAKFAST**		
Food/Drink	Description and Preparation	Amount

MID MORNING- between breakfast time and lunch time		
Food/Drink	Description and Preparation	Amount
**LUNCH**		
Food/Drink	Description and Preparation	Amount

TEA		
Food/Drink	Description and Preparation	Amount
**EVENING MEAL**		
Food/Drink	Description and Preparation	Amount

LATER EVENING- up to last thing at night		
Food/Drink	Description and Preparation	Amount

BETWEEN MEALS, SNACKS AND DRINKS *if not already written in before*		
Food/Drink	Description and Preparation	Amount
Chocolate		
Toffees, sweets		
Crisps, peanuts		
Other snacks		
Beer, wine		
Sherry, Spirits		
Other cold drinks		
Tea, coffee		
Other hot drinks		
Ice cream		
Anything else?		

Space to write in the recipe or ingredients of any home-made dishes, take-away meals etc. that you have mentioned but not described previously. Where applicable, please list amounts of ingredients and brand names. For recipes, take-away meals etc., **please indicate amount/proportion actually consumed by yourself.**

END OF DAY No. 7

GENERAL QUESTIONS ABOUT YOUR FOOD/DRINK LAST WEEK

1. Which type of milk did you most often use last week? **Select one only**

Full Cream, silver ☐ Semi-skimmed, red/white ☐
Skimmed, fat free ☐ Channel Islands, gold ☐
Sterilized ☐ Dried milk ☐
Soya ☐ State type ______ Homogenized ☐
None ☐
Other ☐ State type ______

2. How much milk do you usually have in tea?

A lot ☐ Average ☐ Hardly any ☐
None ☐ I did not drink tea ☐ Hardly any ☐

3. How much milk do you usually have in coffee?

A lot ☐ Average ☐ Hardly any ☐
None ☐ I did not drink coffee ☐ Hardly any ☐

4. Did you drink decaffeinated tea?

Always ☐ Sometimes ☐ Never ☐

5. Did you drink decaffeinated coffee?

Always ☐ Sometimes ☐ Never ☐

6. Which types of fat did you use last week for baking, frying, spreading and salads? **What did you use it for?**

	Brand name & type used	Baking	Frying	Spreading	Salads
Butter		☐	☐	☐	☐
Low fat spread		☐	☐	☐	☐
Very low fat spread		☐	☐	☐	☐
Polyunsaturated margarine		☐	☐	☐	☐
Other soft margarine		☐	☐	☐	☐
Hard margarine		☐	☐	☐	☐
Vegetable oils		☐	☐	☐	☐
White vegetable fat		☐	☐	☐	☐
Lard		☐	☐	☐	☐
Dripping		☐	☐	☐	☐
Other		☐	☐	☐	☐

7. Which type of bread did you eat most often last week? Select one only

White ☐ Soft grain ☐ Granary ☐

Brown, wheatgerm, Hovis ☐ Wholemeal ☐

Other ☐ *Name of 'Other'* [ ]

8. If you ate butter, margarine or spread last week, please tick boxes below to show whether you ate it on toast, bread or in sandwiches:

	Always	Sometimes	Never	Don't Know
Toast	☐	☐	☐	☐
Bread	☐	☐	☐	☐
Sandwiches	☐	☐	☐	☐

9. How thickly did you eat spread, butter, margarine etc. on bread or biscuits?

Thick ☐ Medium ☐ Thin ☐ None ☐

10. If you ate grilled, fried, barbequed or roast meat last week, how well cooked was it?

**Beef, lamb, pork**		**Poultry**	
Well done/dark brown	☐	Well done/dark brown	☐
Medium	☐	Medium	☐
Lightly cooked/rare	☐	Lightly cooked/rare	☐
Did not eat beef, lamb, pork cooked by these methods	☐	Did not eat poultry cooked by these methods	☐

11. If you ate meat last week, what did you do with the visable fat?

Ate all of the fat ☐ Ate most of the fat ☐

Ate some of the fat ☐ Ate as little as possible ☐

Did not eat meat ☐

12. If you ate poultry last week, did you eat the skin?

Yes ☐ No ☐ Sometimes ☐

Don't know ☐ Did not eat poultry ☐

13. If you had gravy last week, were the meat juices, pan residues or dripping put into the gravy?

Yes ☐ No ☐ Sometimes ☐

Don't know ☐ Didn't have gravy ☐

14. Was salt usually added to your food during cooking last week?

Yes ☐ No ☐ Don't know ☐

Did you usually add salt to your food at the table last week?

Yes ☐ No ☐ Don't know ☐

Did you regularly use a salt substitute (e.g. LoSalt) last week?

Yes ☐ No ☐ Don't know ☐

If YES, which brand [ ]

15. Did you eat the skin on fruit? Please tick boxes.

	**Skin eaten**	**Skin not eaten**	**Fruit not eaten**
**Apple**	☐	☐	☐
**Pear**	☐	☐	☐

16. Please name any vitamins, minerals or other food supplements taken on each day of last week. Please write down all the details from each packet/container, and enclose label(s) giving ingredients and individual amounts where possible.

Brand	Name (please list full name)	Amount taken per day- number of pills, capsules or teaspoons	Tick box (es) to show which day(s) supplement was taken last week						
			M	T	W	T	F	S	S
Healthcrafts	*Multivitamins with iron and calcium*	*1 tablet*	✓	✓	✓		✓	✓	✓

EXAMPLE

17. Please complete the table below to show which types of water you consumed last week. Tick which type was used most often for both HOT and COLD drinks.

Water Type	Hot drinks	Cold drinks
Tap water		
Filtered water-hard water filter		
Filtered water-other		
Bottled water-please write down brand		
Other water-please write down name		

18. Were any of the following foods which you ate last week produced organically (without pesticides)? Please tick the necessary box(es).

- Vegetables, homegrown ☐
- Vegetables, purchased ☐
- Fruit, homegrown ☐
- Fruit, purchased ☐
- Milk and dairy products ☐
- Cereals, bread or cereal products ☐
- Meat ☐
- No organic foods eaten ☐

This space has been left for you to tell us about anything else which you feel is important about your food/drink intake last week.

…………………………………………………………………………………………
…………………………………………………………………………………………
…………………………………………………………………………………………
…………………………………………………………………………………………
…………………………………………………………………………………………
…………………………………………………………………………………………
…………………………………………………………………………………………
…………………………………………………………………………………………
…………………………………………………………………………………………

This document is reproduced with the permission of the EPIC Study Team in Cambridge

Study Number: □□□□

BLADDER CANCER PROGNOSIS PROGRAMME

Cancer Research UK Bladder Cancer Group

Follow-up Questionnaire

CONFIDENTIAL

VERSION: FINAL 1.0 November 2005

PLEASE DESTROY PREVIOUS VERSIONS

MREC APPROVAL: 23rd November 2005

START DATE: 1st January 2006

A programme of research

Conducted by: UNIVERSITY OF BIRMINGHAM

Funded by: CANCER RESEARCH UK

	BLADDER CANCER PROGNOSIS PROGRAMME (BCPP)	FOLLOW-UP QUESTIONNAIRE<br><br>**INTRODUCTION & INTERVIEW DETAILS**
CANCER RESEARCH UK		

Patient Identifier:<br>*First 3 letters from the surname followed by first 2 letters from the forename*			☐☐☐☐☐<br>*eg: John Smith = SMIJO*
Date of Interview:	D D M M Y Y Y Y	Name of Interviewer:	___________________
Time Interview Started<br>*(24hour)*	☐☐ : ☐☐	Hospital Name:	___________________
Will the interview take place with the support of a translator			Yes ☐ No ☐

INTRODUCTION

Some time ago we asked you to tell us some information about yourself and your lifestyle as part of the research study called the Bladder Cancer Prognosis Programme. In order for us to have really good information for our study, we need to know if any of the things we asked you about before have changed since you completed the last questionnaire.

We would therefore like to take you through some questions that ask about any changes that have occurred since last time, so that we can add this new information to the existing information. The questionnaire is much shorter than the first one and will take approximately half an hour.

The questions that you will be asked will include questions about your lifestyle, your behaviours, your health and the help and support you receive from the people around you.

There may be some questions that you think are unusual. The questions are not used to test you in any way and the responses that you give will not be used to make any judgements of you. There are no right or wrong answers. All of your responses will be treated as strictly confidential and will be used only for medical research.

Some of the questions ask for personal information. If you feel that any of the questions are too personal, do not answer them. However, by answering these questions, you will help us to discover links between lifestyles and health.

I <research nurse> will take you through each question and keep a record of your responses. There is no time limit, so if you want a little time to think about any of the questions please do so. Try to answer every question even if the answer is ‘I can’t remember’ or ‘I don’t know’.

If you have any other questions or if there is anything that you feel you don’t understand, please ask me <research nurse> at any time.

Thank you again for taking the time to participate in this study

SECTION 1: SMOKING BEHAVIOUR

1.1. Have you smoked at all since <month and year of last questionnaire>

Yes ☐ ***Go to question 1.2***

No ☐ ***Go to question 4.1***

1.2. Please tell us during which months since <month and year of last questionnaire> you have smoked each of the following types of tobacco and how frequently.
(Only fill in the months between last questionnaire and current questionnaire)

	FILTER CIGARETTES			NON-FILTER / HAND-ROLLED CIGARETTES		
	Have you smoked Filter Cigarettes at all during the following months?		If ***Yes***, How many per week?	Have you smoked Non-Filter or hand rolled Cigarettes at all during the following months?		If ***Yes***, How many per week?
	**YES**	**NO**		**YES**	**NO**	
January	☐	☐	|__|__|	☐	☐	|__|__|
February	☐	☐	|__|__|	☐	☐	|__|__|
March	☐	☐	|__|__|	☐	☐	|__|__|
April	☐	☐	|__|__|	☐	☐	|__|__|
May	☐	☐	|__|__|	☐	☐	|__|__|
June	☐	☐	|__|__|	☐	☐	|__|__|
July	☐	☐	|__|__|	☐	☐	|__|__|
August	☐	☐	|__|__|	☐	☐	|__|__|
September	☐	☐	|__|__|	☐	☐	|__|__|
October	☐	☐	|__|__|	☐	☐	|__|__|
November	☐	☐	|__|__|	☐	☐	|__|__|
December	☐	☐	|__|__|	☐	☐	|__|__|
January	☐	☐	|__|__|	☐	☐	|__|__|
February	☐	☐	|__|__|	☐	☐	|__|__|
March	☐	☐	|__|__|	☐	☐	|__|__|
April	☐	☐	|__|__|	☐	☐	|__|__|
May	☐	☐	|__|__|	☐	☐	|__|__|
June	☐	☐	|__|__|	☐	☐	|__|__|
July	☐	☐	|__|__|	☐	☐	|__|__|
August	☐	☐	|__|__|	☐	☐	|__|__|
September	☐	☐	|__|__|	☐	☐	|__|__|
October	☐	☐	|__|__|	☐	☐	|__|__|
November	☐	☐	|__|__|	☐	☐	|__|__|
December	☐	☐	|__|__|	☐	☐	|__|__|

SMOKING BEHAVIOUR *(Continued)*

	CIGARS			PIPE		
	Have you smoked Cigars at all during the following months?		If ***Yes***, How many per week?	Have you smoked the Pipe at all during the following months?		If ***Yes***, How many per week?
	**YES**	**NO**		**YES**	**NO**	
January	☐	☐	__ __	☐	☐	__ __
February	☐	☐	__ __	☐	☐	__ __
March	☐	☐	__ __	☐	☐	__ __
April	☐	☐	__ __	☐	☐	__ __
May	☐	☐	__ __	☐	☐	__ __
June	☐	☐	__ __	☐	☐	__ __
July	☐	☐	__ __	☐	☐	__ __
August	☐	☐	__ __	☐	☐	__ __
September	☐	☐	__ __	☐	☐	__ __
October	☐	☐	__ __	☐	☐	__ __
November	☐	☐	__ __	☐	☐	__ __
December	☐	☐	__ __	☐	☐	__ __
January	☐	☐	__ __	☐	☐	__ __
February	☐	☐	__ __	☐	☐	__ __
March	☐	☐	__ __	☐	☐	__ __
April	☐	☐	__ __	☐	☐	__ __
May	☐	☐	__ __	☐	☐	__ __
June	☐	☐	__ __	☐	☐	__ __
July	☐	☐	__ __	☐	☐	__ __
August	☐	☐	__ __	☐	☐	__ __
September	☐	☐	__ __	☐	☐	__ __
October	☐	☐	__ __	☐	☐	__ __
November	☐	☐	__ __	☐	☐	__ __
December	☐	☐	__ __	☐	☐	__ __

SECTION 2: DIETARY BEHAVIOURS

The questions in this section ask about your normal diet since *<month and year of last questionnaire>*.

2.1. Please indicate how often on average, since *<month and year of last questionnaire>*, you have eaten each of the food types that are listed below

	**Average Use Since** *<month and year of last questionnaire>*					
	**Never or less than once per month**	**1-3 per month**	**Once a week**	**2-4 per week**	**5-6 per week**	**At least once per day**
STAPLE FOODS						
Bread………………………………………...	☐	☐	☐	☐	☐	☐
Potatoes……………………………………..	☐	☐	☐	☐	☐	☐
Pasta………………………………………… (eg. Macaroni, spaghetti)	☐	☐	☐	☐	☐	☐
Rice………………………………………….	☐	☐	☐	☐	☐	☐
Noodles……………………………………...	☐	☐	☐	☐	☐	☐
Wheat……………………………………….. (eg. Whole grain bread)	☐	☐	☐	☐	☐	☐
Cereal………………………………………. (eg. Oats, bran, corn)	☐	☐	☐	☐	☐	☐
MEAT						
Meat (no organs)…………………………. (eg. pork, steak, beef, lamb)	☐	☐	☐	☐	☐	☐
Organ Meat…………………………………. (eg. Liver, Heart, Kidney)	☐	☐	☐	☐	☐	☐
Chicken………………………………………	☐	☐	☐	☐	☐	☐
Other Poultry………………………………... (eg. Goose, Duck, Pigeon)	☐	☐	☐	☐	☐	☐
FISH						
Dark fleshed fish……………………………… (eg. mackerel, salmon, tuna, anchovies)	☐	☐	☐	☐	☐	☐
White fleshed fish…………………………… (eg. cod, haddock, hake, halibut, sea bass, skate, sole, plaice)	☐	☐	☐	☐	☐	☐
Seafood……………………………………… (eg. Prawn, Crab, Lobster, Cockles, Winkles, Squid, Octopus, Mussels)	☐	☐	☐	☐	☐	☐
VEGETABLES						
Fruit Vegetables………………………… (eg. Tomato, Cucumber, Aubergine)	☐	☐	☐	☐	☐	☐

DIETARY BEHAVIOURS (Continued)

	**Average Use Since** *<month and year of last questionnaire>*					
	Never or less than once per month	1-3 per month	Once a week	2-4 per week	5-6 per week	At least once per day
VEGETABLES (Continued)						
Flower vegetables……………………………<br>(eg. Broccoli, Cauliflower)	☐	☐	☐	☐	☐	☐
Leafy vegetables……………………………….<br>(eg. Spinach, Cabbage, Lettuce)	☐	☐	☐	☐	☐	☐
Stem vegetables……………………………….<br>(eg. Asparagus, Celery, Fennel)	☐	☐	☐	☐	☐	☐
Mushrooms……………………………………..	☐	☐	☐	☐	☐	☐
Bulbs…………………………………………….<br>(eg. Onion, Garlic, Leek, Shallot)	☐	☐	☐	☐	☐	☐
Roots…………………………………………….<br>(eg. Beetroot, Swede, Carrot, Parsnip)	☐	☐	☐	☐	☐	☐
FRUIT						
Citrus Fruits…………………………………….<br>(eg. Orange, Lemon, Lime, Grapefruit)	☐	☐	☐	☐	☐	☐
Stone Fruits…………………………………….<br>(eg. Plum, Apricot, Peach, Cherry)	☐	☐	☐	☐	☐	☐
Soft Fruits……………………………………….<br>(eg. Raspberry, Strawberry, Redcurrant, Blackberry)	☐	☐	☐	☐	☐	☐
Fleshy Fruits…………………………………….<br>(eg. Apple, Pear, Banana, Pineapple)	☐	☐	☐	☐	☐	☐
Vine Fruits……………………………………….<br>(eg. Grape, Melon, Cantaloupe)	☐	☐	☐	☐	☐	☐
DAIRY						
Cream…………………………………………...	☐	☐	☐	☐	☐	☐
Butter / Margarine……………………………..	☐	☐	☐	☐	☐	☐
Yogurt…………………………………………..	☐	☐	☐	☐	☐	☐
Cheese …………………………………………	☐	☐	☐	☐	☐	☐
Egg ……………………………………………..	☐	☐	☐	☐	☐	☐
OTHER FOODS						
Pulses…………………………………………...<br>(eg. Pea, Bean, Lentil)	☐	☐	☐	☐	☐	☐
Nuts and Seeds……………………………….	☐	☐	☐	☐	☐	☐
Soy/Tofu products……………………………<br>(eg. Soy milk, Tofu, Soya meat)	☐	☐	☐	☐	☐	☐
Sweets and snacks…………………………….<br>(eg. Crisps, cakes, ice cream, chocolate)	☐	☐	☐	☐	☐	☐

FLUID INTAKE

2.2. Please indicate how often, *<month and year of last questionnaire>*, you have drunk one measure each of the types of drinks that are listed below.

	Measure	Never or less than one measure per month	1-3 per month	One a week	2-4 per week	5-6 per week	At least one per day	How many per day?
		Average Use Since <month and year of last questionnaire>						
**ALCOHOLIC DRINKS**								
Wine or champagne………	1 small glass	☐	☐	☐	☐	☐	☐ →	☐☐
Fortified Wine………………. (eg. Port, Sherry, Cinzano)	1 small glass	☐	☐	☐	☐	☐	☐ →	☐☐
Beer…………………………. (eg. Beer, Lager, Stout)	1 Pint	☐	☐	☐	☐	☐	☐ →	☐☐
Cider…………………………	1 Pint	☐	☐	☐	☐	☐	☐ →	☐☐
Spirits……………………….. (eg. Gin, Brandy, Rum, Vodka, Whisky)	1 pub measure (25cl)	☐	☐	☐	☐	☐	☐ →	☐☐
Liqueurs…………………….. (eg. Tia Maria, Cointreau, Baileys, Grand Marnier)	1 pub measure (25cl)	☐	☐	☐	☐	☐	☐ →	☐☐
**HOT DRINKS**								
Coffee………………………	1 cup	☐	☐	☐	☐	☐	☐ →	☐☐
Tea…………………………..	1 cup	☐	☐	☐	☐	☐	☐ →	☐☐
Hot Chocolate………………	1 cup	☐	☐	☐	☐	☐	☐ →	☐☐
Ovaltine / Horlicks…………..	1 cup	☐	☐	☐	☐	☐	☐ →	☐☐
Soup…………………………	1 Cup / Bowl	☐	☐	☐	☐	☐	☐ →	☐☐
**SOFT DRINKS**								
Fizzy pop…………………… (eg. Lemonade, Cola)	½ pint glass	☐	☐	☐	☐	☐	☐ →	☐☐
Pure fruit juice………………. (eg. Orange, Apple, etc)	½ pint glass	☐	☐	☐	☐	☐	☐ →	☐☐
Fruit squash or cordial……..	½ pint glass	☐	☐	☐	☐	☐	☐ →	☐☐
Milk…………………………..	½ pint glass	☐	☐	☐	☐	☐	☐ →	☐☐
Water ………………………..	½ pint glass	☐	☐	☐	☐	☐	☐ →	☐☐

SECTION 3: VITAMINS AND SUPPLEMENTS

The questions in this section ask about your use of vitamins and dietary supplements. If you are participating in the Randomised Trial connected with this research, we do not need you to tell us about the vitamins that you have been given as part of the trial.

3.1. Have you taken, any of the following vitamins or supplements since *<month and year of last questionnaire>*?

Type of Vitamin / Supplement	Yes	no	Unknown	What is the name of your medication?	Where do you buy this product?
Example Example Example Example Example Example					
Multi-vitamins	☐	☑	☐	***ABC Plus Tablets***	***Holland and Barrett***
Multi-vitamins	☐	☐	☐		
Folic Acid	☐	☐	☐		
Vitamin B	☐	☐	☐		
Vitamin C	☐	☐	☐		
Vitamin E	☐	☐	☐		
Iron pills	☐	☐	☐		
Cod liver oil	☐	☐	☐		
Magnesium	☐	☐	☐		
Zinc	☐	☐	☐		
Vitamin B12	☐	☐	☐		
Selenium	☐	☐	☐		
Chromium	☐	☐	☐		
Calcium	☐	☐	☐		
Other (1)	☐	☐	☐		
Other (2)	☐	☐	☐		

If you are taking any vitamins or supplements. Please bring the packet in with you on your next hospital visit

CANCER RESEARCH UK	BLADDER CANCER PROGNOSIS PROGRAMME (BCPP)	FOLLOW-UP QUESTIONNAIRE<br>**MEDICATIONS**<br>Section 4

SECTION 4: MEDICATIONS

Please indicate which medications you have taken since *<month and year of last questionnaire>*. Where possible, please also record the name of the of the medication that you have taken

4.1. Have you taken any of the following medications since *<month and year of last questionnaire>*, regularly for a period of 1 month or more?

Type of Medicine	Yes	No	Unknown	What is the name of your medication?
Example Example Example Example Example Example				
NSAID's	☑	☐	☐	***Ibuprofen***

Pain killers				
Paracetamol	☐	☐	☐	
Aspirin	☐	☐	☐	
NSAID's (eg. Ibuprofen, Brufen, Diclofenac, Volterol)	☐	☐	☐	
Medicine for high cholesterol (eg. Simvastatin, Pravastatin, Atorvastatin)	☐	☐	☐	

4.2. Have you taken any of the following medications at any time since *<month and year of last questionnaire>*?

Type of Medicine	Yes	No	Unknown	What is the name of your medication?
Antidepressants, sleeping pills or sedatives	☐	☐	☐	
Chemotherapy	☐	☐	☐	
Immune Suppressants (eg. Steroid Tablets, Cyclosporin)	☐	☐	☐	
Inhaled Steroids	☐	☐	☐	
**Women Only:** Hormone replacement therapy (HRT)	☐	☐	☐	
**Women Only:** The contraceptive pill	☐	☐	☐	

	BLADDER CANCER PROGNOSIS PROGRAMME (BCPP)	FOLLOW-UP QUESTIONNAIRE<br>**MEDICAL HISTORY**<br>Section 5

SECTION 5: MEDICAL HISTORY

The questions in this section ask about medical conditions you might have had since *<month and year of last questionnaire>*.

5.1. Have you been diagnosed with an illness or medical condition other than your bladder cancer since <month and year of last questionnaire>.

Condition #1

Name of Condition: ______________________

Month of diagnosis: ☐☐ Month (eg. Jan = 01) ☐☐☐☐ Year (eg. 2005)

Condition #2

Name of Condition: ______________________

Month of diagnosis: ☐☐ Month (eg. Jan = 01) ☐☐☐☐ Year (eg. 2005)

Condition #3

Name of Condition: ______________________

Month of diagnosis: ☐☐ Month (eg. Jan = 01) ☐☐☐☐ Year (eg. 2005)

Condition #4

Name of Condition: ______________________

Month of diagnosis: ☐☐ Month (eg. Jan = 01) ☐☐☐☐ Year (eg. 2005)

Condition #5

Name of Condition: ______________________

Month of diagnosis: ☐☐ Month (eg. Jan = 01) ☐☐☐☐ Year (eg. 2005)

Condition #6

Name of Condition: ______________________

Month of diagnosis: ☐☐ Month (eg. Jan = 01) ☐☐☐☐ Year (eg. 2005)

SECTION 6: STANDARD GAMBLE
- **Only Suitable for Patients Undergoing Cystoscopic Surveillance**
- **Not to be included in reassessment following recurrence or progression**

In this section, we would like you to consider a made-up situation and tell us how you would respond if it were a real situation.

Imagine that there is a new test available which claims to predict whether you will remain free of bladder cancer. The new test does not require you to have any internal examination of your bladder but will simply test samples of your blood, urine or bladder tissue that have already been collected.

Your urologist advises that if you take this test today, you will not have to undergo yearly cystoscopy anymore for the rest of your life, although you will have all other follow-up and treatment just as you are now.

However, the new test may not be 100% accurate, and so there is a chance that your bladder cancer could come back even if the test says it won't. So by taking the new test, and not having regular cystoscopies, this inaccuracy may mean if your cancer does come back, it might be missed. There is no way of knowing which patients will be predicted correctly by this new test.

Your urologist offers you the choice between having the test and no more cystoscopies or to continue with regular cystoscopies. Your urologist will support whatever decision you make.

	**YES**	**NO**
6.1. Would you take the new test today if you knew that out of **1 thousand** patients …		
…the test correctly predicts no recurrence in all 1000 patients (i.e. 100% accurate)	☐	☐
…the test correctly predicts no recurrence for 999 patient but misses 1 (i.e. 99.9% accurate)	☐	☐
…the test correctly predicts no recurrence for 990 patients but misses 10 (i.e. 99% accurate).	☐	☐
…the test correctly predicts no recurrence for 970 patients but misses 30 (i.e. 97% accurate).	☐	☐
…the test correctly predicts no recurrence for 950 patients but misses 50 (i.e. 95% accurate).	☐	☐
…the test correctly predicts no recurrence for 900 patients but misses 100 (i.e. 90% accurate).	☐	☐

STANDARD GAMBLE *(Continued)*

- **Only Suitable for Patients Undergoing Cystoscopic Surveillance**
- **Not to be included in reassessment following recurrence or progression**

	**YES**	**NO**
…the test correctly predicts no recurrence for 800 patients but misses 200. (i.e. 80% accurate).	☐	☐
…the test correctly predicts no recurrence for 700 patients but misses 300 (i.e. 70% accurate).	☐	☐
…the test correctly predicts no recurrence for 600 patients but misses 400 (i.e. 60% accurate).	☐	☐
…the test correctly predicts no recurrence for 500 patients but misses 500 (i.e. 50% accurate).	☐	☐
…the test correctly predicts no recurrence for 400 patients but misses 600 (i.e. 40% accurate).	☐	☐
…the test correctly predicts no recurrence for 300 patients but misses 700. (i.e. 30% accurate)	☐	☐
…the test correctly predicts no recurrence for 200 patients but misses 800. (i.e. 20% accurate)	☐	☐
…the test correctly predicts no recurrence for 100 patients but misses 900. (i.e. 10% accurate)	☐	☐
…the test correctly predicts no recurrence for 0 patients but misses 1000. (i.e. 100% inaccurate)	☐	☐

	BLADDER CANCER PROGNOSIS PROGRAMME (BCPP)	FOLLOW-UP QUESTIONNAIRE<br>**SOCIAL SUPPORT**<br>Section 7
CANCER RESEARCH UK		

SECTION 7: SOCIAL SUPPORT **- (to be completed by the patient)**

Here is a list of some things that other people do for us or give us that may be helpful or supportive. Please read each statement carefully and put a tick the box that is closest to your situation.

Example Example Example Example Example Example

	As much as I would like				Much less than I would like
I get enough vacation time……………………………	☐	☑	☐	☐	☐

If you put a check where we have, it means that you get *almost* as much vacation time as you would like, but not quite as much as you would like.

		As much as I would like				Much less than I would like
7.1.	I have people who care what happens to me…….	☐	☐	☐	☐	☐
7.2.	I get love and affection……………………………..	☐	☐	☐	☐	☐
7.3.	I get chance to talk to someone I trust about problems at work or with my housework …………	☐	☐	☐	☐	☐
7.4.	I get chance to talk to someone I trust about my personal and family problems……………………..	☐	☐	☐	☐	☐
7.5.	I get chance to talk about money matters ………..	☐	☐	☐	☐	☐
7.6.	I get invitations to go out and do things with other people ………………………………………………..	☐	☐	☐	☐	☐
7.7.	I get useful advice about important things in life ...	☐	☐	☐	☐	☐
7.8.	I get help when I am sick in bed …………………..	☐	☐	☐	☐	☐

SECTION 8: GENERAL HEALTH - (to be completed by the patient)

We are interested in some things about you and your health. Please answer all of the questions yourself by circling the number between 1 and 4 that best applies to you. There are no "right" or "wrong" answers. The answers that you provide will remain strictly confidential.

		Not at all	A little	Quite a bit	Very much
8.1.	Do you have any trouble doing strenuous activities, like carrying a heavy shopping bag or a suitcase?.....	1	2	3	4
8.2.	Do you have any trouble taking a long walk?...........	1	2	3	4
8.3.	Do you have any trouble taking a short walk outside of the house?...	1	2	3	4
8.4.	Do you need to stay in bed or a chair during the day?..	1	2	3	4
8.5.	Do you need help with eating, dressing, washing yourself or using the toilet?.......................................	1	2	3	4

**During the past week:**		Not at all	A little	Quite a bit	Very much
8.6.	Were you limited in doing either your work or other daily activities?...	1	2	3	4
8.7.	Were you limited in pursuing your hobbies or other leisure time activities?...	1	2	3	4
8.8.	Were you short of breath?.......................................	1	2	3	4
8.9.	Have you had pain?...	1	2	3	4
8.10.	Did you need to rest?..	1	2	3	4
8.11.	Have you had trouble sleeping?......................	1	2	3	4
8.12.	Have you felt weak?......................................	1	2	3	4
8.13.	Have you lacked appetite?.............................	1	2	3	4

GENERAL HEALTH *(Continued)* - **(to be completed by the patient)**

**During the past week:**	**Not at all**	**A little**	**Quite a bit**	**Very much**
8.14. Have you felt nauseated?..	1	2	3	4
8.15. Have you vomited?..	1	2	3	4
8.16. Have you been constipated?....................................	1	2	3	4
8.17. Have you had diarrhoea?...	1	2	3	4
8.18. Were you tired?...	1	2	3	4
8.19. Did pain interfere with your daily activities?..............	1	2	3	4
8.20. Have you had difficulty in concentrating on things, like reading a newspaper or watching television?..........	1	2	3	4
8.21. Did you feel tense?..	1	2	3	4
8.22. Did you worry?...	1	2	3	4
8.23. Did you feel irritable?...	1	2	3	4
8.24. Did you feel depressed?..	1	2	3	4
8.25. Have you had difficulty remembering things?...........	1	2	3	4
8.26. Has your physical condition or medical treatment interfered with your <u>family</u> life?...................................	1	2	3	4
8.27. Has your physical condition or medical treatment interfered with your <u>social</u> life?...................................	1	2	3	4
8.28. Has your physical condition or medical treatment caused you financial difficulties?................................	1	2	3	4

For the following questions, please circle the number between 1 and 7 that best applies to you

8.29. How would you rate your overall health during the past week?

1 2 3 4 5 6 7

<u>Very Poor</u> ……………………………………………………………………………………….. <u>Excellent</u>

8.30. How would you rate your overall quality of life during the past week?

1 2 3 4 5 6 7

<u>Very Poor</u> ……………………………………………………………………………………… <u>Excellent</u>

GENERAL HEALTH *(Continued)* **- (to be completed by the patient)**

**During the past week:**		**Not at all**	**A little**	**Quite a bit**	**Very much**
8.31.	Did you have a fever?..	1	2	3	4
8.32.	Did you feel ill or unwell?...	1	2	3	4
8.33.	Did you have trouble arranging your life around repeated bladder treatment appointments (cystoscopies or instillations)?....................................	1	2	3	4
8.34.	Did you worry about having repeated bladder treatment appointments (cystoscopies or instillations)?................	1	2	3	4
8.35.	Were you worried about your health in the future?......	1	2	3	4
8.36.	Did you worry about results of examinations or tests?	1	2	3	4
8.37.	Did you worry about possible future treatments?.........	1	2	3	4
8.38.	Did you have a bloated feeling in your abdomen?........	1	2	3	4
8.39.	Have you had flatulence or gas?.................................	1	2	3	4
8.40.	Have you felt physically less attractive as a result of your illness or treatment?..	1	2	3	4
8.41.	Have you been dissatisfied with your body?..............	1	2	3	4
8.42.	Have you felt less feminine/masculine as a result of your illness or treatment?..	1	2	3	4

PLEASE ANSWER QUESTIONS 8.42-8.49 ONLY IF YOU DO NOT HAVE A UROSTOMY

8.43.	Have you had to urinate frequently **during the day**?	1	2	3	4
8.44.	Have you had to urinate frequently **at night**?.............	1	2	3	4
8.45.	When you felt the urge to pass urine, did you have to hurry to get to the toilet?...	1	2	3	4
8.46.	Was it difficult for you to get enough sleep, because you needed to get up frequently at night to urinate?....	1	2	3	4
8.47.	Have you had difficulty going out of the house, because you need to be close to a toilet?...................	1	2	3	4
8.48.	Have you had any unintentional release (leakage) of urine?...	1	2	3	4
8.49.	Have you had pain or a burning feeling when urinating?..	1	2	3	4

GENERAL HEALTH *(Continued)* - (to be completed by the patient)

PLEASE ANSWER QUESTIONS 8.50-8.55 ONLY IF YOU HAVE A UROSTOMY

During the past week:	Not at all	A little	Quite a bit	Very much
8.50. Has urine leaked from your urostomy bag?..............	1	2	3	4
8.51. Did you have problems with caring for your urostomy?	1	2	3	4
8.52. Was your skin around the urostomy irritated?.............	1	2	3	4
8.53. Have you felt embarrassed because of your urostomy?	1	2	3	4
8.54. Have you been dependent on others for caring for your urosomy?..	1	2	3	4
8.55. Did you frequently have to change the urostomy bag?..	1	2	3	4

PLEASE ANSWER QUESTION 8.56 ONLY IF YOU HAVE USED A CATHETER DURING THE PAST WEEK

8.56. Have you had problems with self catheterization? (inserting a tube into the bladder to pass urine)……….	1	2	3	4

During the past 4 weeks:	Not at all	A little	Quite a bit	Very much
8.57. To what extent were you interested in sex?..............	1	2	3	4
8.58. To what extent were you sexually active (with or without sexual intercourse)?......................................	1	2	3	4
8.59. **For men only:** Did you have difficulty gaining or maintaining an erection?..	1	2	3	4
8.60. **For men only:** Did you have ejaculation problems (e.g. dry ejaculation)? …………………………………	1	2	3	4

**Please answer the following 4 questions only if you have been sexually active during the past 4 weeks:**	Not at all	A little	Quite a bit	Very much
8.61. Have you felt uncomfortable about being sexually intimate? ………………………………………………	1	2	3	4
8.62. Have you worried that you may contaminate your partner during sexual contact with the bladder treatment that you have been receiving?....................	1	2	3	4
8.63. To what extent was sex enjoyable for you?..............	1	2	3	4
8.64. **For women only:** did you have a dry vagina or other problems during intercourse?...............................	1	2	3	4

APPENDIX 3
MOLECULAR MARKERS TO BE EXAMINED

FGFR3

FGFR3 belongs to a family of structurally related tyrosine kinase receptors encoded by four different genes, FGFR1-4.[119] The wild type FGFR3 gene is expressed in both normal urothelium and bladder TCC. van Rhijn et al demonstrated that FGFR3 mutations occurred in 34 of 53 pTa tumours, and did not occur in TCCs of stage pT1 or above. Furthermore, all tumours with FGFR3 mutations were grade 1 or 2 only. In addition, recurrence at 12 months was far more frequent in those patients whose initial tumours expressed the wild type FGFR3 gene (61% vs. 21%, p=0.004).[120] Similar results were also found by Billerey et al.[119] FGFR3 mutations thus appear to be associated with favourable prognostic features, and it is suggested that FGFR3-mutated TCCs shed cells less easily and/or have a lower proliferation rate than non-mutated tumours.[120] Interestingly, FGFR3 mutations and TP53 mutations appear to be mutually exclusive, defining separate pathways of transitional cell carcinogenesis.[121,122] We therefore hypothesise that the presence of FGFR3 mutations is an independent predictor of a lower risk of recurrence and progression.

EGFR

The epidermal growth factor receptor (EGFR) is a member of the tyrosine kinase receptor family, a group all encoded by the c-*erb*B oncogenes. It is the activation of the tyrosine kinase domain of EGFR that initiates signal transduction following ligand binding, with ligands including EGF, transforming growth factor-alpha (TGF-α), amphiregulin, betacellulin, heparin-binding EGF-like factor (HB-EGF), and epiregulin. Pathological expression of EGFR leads to uncontrolled cell proliferation, and also increased angiogenesis and reduced apoptosis.[123] Overexpression of EGFR in bladder cancer is widely-reported to be associated with high tumour stage, tumour progression, and poor clinical outcome.[123,124] Inhibition of EGFR activity is considered to be a way of improving the prognosis of patients with bladder cancer and tyrosine kinase inhibition is one approach that is currently being extensively studied in this and other malignancies.[123,125] The combined use of gefitinib (“Iressa”, ZD1839), a small molecule tyrosine kinase inhibitor which targets the epidermal growth factor receptor, with radiotherapy significantly diminishes bladder cancer cell proliferation, both *in vitro* and *in vivo*, compared with either treatment modality used on its own.[126] We hypothesise that overexpression of EGFR is an independent predictor of the risk of progression.

pRB

Most antiproliferative signals utilise the retinoblastoma protein (pRB)[127], with hypophosphorylated pRB blocking proliferation.[128] Altered pRB expression is found to be associated with tumour progression and worse disease-free survival in a number of studies.[129-132] We hypothesise that altered pRB expression is an independent predictor of the risk of progression.

p53

The p53 gene is the most common target of genetic alteration identified in human cancers, and cells with inactivated p53 have a selective growth advantage.[133] Normal p53 function can be lost in a number of ways but most commonly by loss of a chromosomal region containing one allele of the gene and a subtle mutation involving the other allele (found on chromosome 17p) [reviewed by Nakamura[134]]. p53 is considered to be the “guardian of the genome” as it can induce either apoptosis or DNA repair.[135] Following DNA damage, increased levels of p53 protein causes cell cycle arrest and subsequently induces apoptosis.[136-138] p53-target genes have a number of other physiological roles including inhibiting angiogenesis, ameliorating oxidative stress, and immuno-surveillance.[134] p53 protein is constitutively expressed in all cell types but does not accumulate due to its rapid degradation by the proteasome, the degradation initiated by Mdm2.[139] The majority of p53 mutations are stabilising, allowing p53 protein to accumulate and immunostain. Up to 10% of bladder tumours will have p53 gene mutations and approximately 66% of tumours have dysregulated p53 at the protein level, which is strongly related to tumour invasiveness. [140] Typically, p53 alterations are associated with advanced bladder cancers, although there may be a role early in transitional cell carcinogenesis as p53 deficiency predisposes the urothelium to hyperproliferation [141].

The evidence supporting the role of abnormal p53 expression in urothelial carcinogenesis appears very strong [117,142-144], and that its effect has not been assessed in a multivariate manner with the other potentially important markers (except with pRB[117]). In this context, we therefore hypothesise that abnormal p53 expression is an independent predictor of recurrence and progression.

Our results will complement the combined retrospective analysis by The International Study Initiative on Bladder Cancer[145] (which does not include other markers).

Ki-67

Ki-67 expression (detected with the MIB-1 antibody) is a marker of tumour proliferation. In a study of 31 patients who had undergone radical cystectomy for bladder cancer Tsuji et al demonstrated that patients whose tumour samples had a high Ki-67 index (>32%) had a

significantly worse prognosis than those with a lower index.[146] Liukkonen et al studied 207 patients with Ta/T1 bladder cancer followed up for 4.9 years. Using multivariate analysis they demonstrated that MIB-1 score was an independent predictor of progressive disease and cancer-specific survival.[147] Similarly, Pfister et al studied 319 patients with newly-diagnosed Ta/T1 bladder cancer and found that a Ki-67 index greater than 10% was an independent predictor of tumour recurrence among patients with large tumours (> 3cm).[148] We hypothesise that the proliferative index, as assessed by Ki-67 expression stained with MIB-1, is an independent predictor of recurrence and progression.

VEGF

Vascular Endothelial Growth Factor (VEGF) is a pro-angiogenic signal[149], with sustained angiogenesis being an important factor in allowing proliferative lesions to increase their capability for growth and invasion. Sustained angiogenesis also appears to play an important role both early and late in bladder transitional cell carcinogenesis.[14] High expression of VEGF mRNA in 55 Ta/T1 bladder TCCs was associated with earlier recurrence and progression.[150] High pre-operative urinary VEGF levels are associated with recurrence.[151] We hypothesise that high expression of VEGF is an independent predictor of recurrence and progression.

CK20

Cytokeratins are components of the intermediate filaments of the cell cytoskeleton and are characteristic of epithelial cells.[152] In normal urothelium CK20 expression is restricted to the superficial umbrella cells and occasional intermediate cells. Loss of this restriction was demonstrated in 31 of 36 cases of urothelial dysplasia (86%), with positive expression in all layers of the urothelium.[153] A normal CK20 expression appears to be predictive of tumour non-recurrence. Harnden et al showed that out of 58 consecutive patients with non-invasive papillary bladder tumours 10 had a normal pattern of CK20 expression and none of these 10 tumours developed further recurrence during the median follow-up of 18 months. By contrast, 30 of 41 evaluable patients with tumours that showed abnormal CK20 expression (73%) developed further tumours, with a median time to a second tumour of 6 months.[154] Similarly, Alsheikh et al showed that in 16 patients with tumours with a normal pattern of CK20 expression, four experienced a recurrence, in contrast to the 15 of 30 patients with abnormal CK20 staining who experienced one or more recurrences.[155] We hypothesise that normal CK20 expression is an independent predictor of a lower risk of recurrence.

APPENDIX 4
SITE PARTICIPATION FORM

Responsible Urologist (Principal Investigator): …………………………………………

Centre Name: ………………………………………………………………………..

Address: ………………………………………………………………………..

………………………………………………………………………..

Tel: ……………………………….. Fax: …………………………………

E-mail: ………………………………………………………………………..

- ❖ We want to participate in the Bladder Cancer Prognosis Programme (BCPP).
- ❖ We will enter all patients meeting the BCPP eligibility criteria.
- ❖ We will undertake to respect the BCPP protocol, but our medical responsibility takes precedence over the protocol.
- ❖ We will provide all of the necessary information and specimens.

(Please provide a name and signature for each of the clinical team members listed below. This list should include all other Urologists at your centre)

<u>All other Urologists:</u>

1. Name: …………………………………. Signature: ,,

2. Name: …………………………………. Signature: ,,

3. Name: …………………………………. Signature: ,,

4. Name: …………………………………. Signature: ,,

5. Name: …………………………………. Signature: ,,

6. Name: …………………………………. Signature: ,,

(Continue on another sheet if necessary)

Pathologist: Name: …………………………….. Signature: …………………………...

Oncologist: Name: …………………………….. Signature: …………………………..

<u>Please return this form to:</u> The BCPP Study Office, Room G08, Public Health Building, Department of Public Health and Epidemiology, The University of Birmingham, B15 2TT

APPENDIX 5
BCPP PATIENT PATHWAY

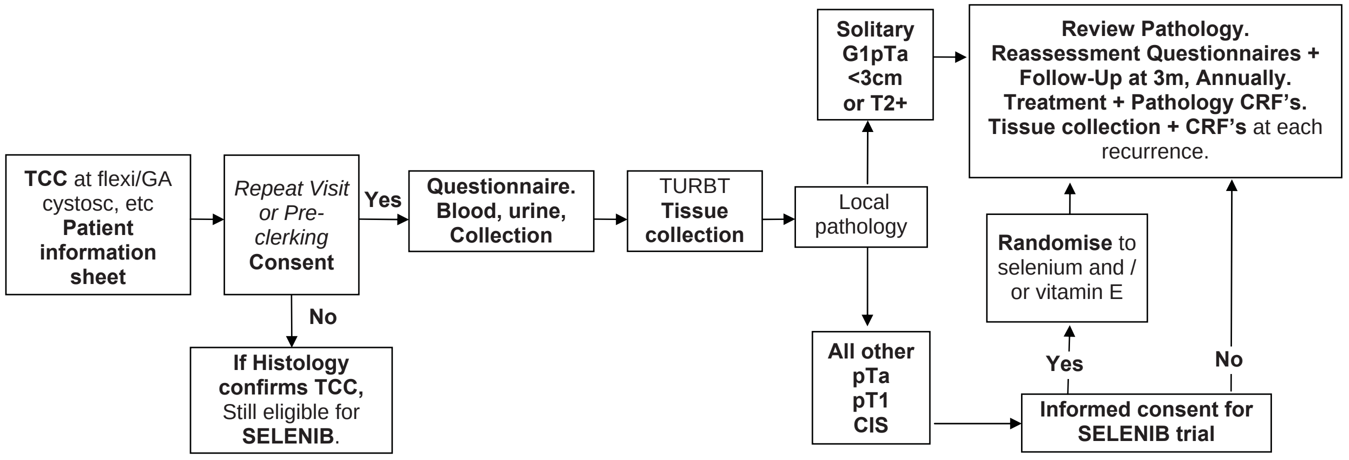

APPENDIX 6
SCHEDULE OF CRF RETURN

Form Number	Form Name	Enter / Return by
Form 1a	Registration	At time of obtaining consent
Form 1b	Cystoscopy	Within 1 week of registration
Form 2a	TURBT	Within 2 weeks of surgery
Form 2b	Cystectomy & Staging Investigations	Within 2 weeks of surgery
Form 3a	Local Pathology	Within 2 weeks of surgery (This must be prior to SELENIB randomisation)
Form 3b	Review Pathology	Not specified
Form 4b	Pre and Post Operative Treatment	within 1 week of end of treatment
Form 5	End of primary treatment	within 1 week of end of treatment
Form 6	Follow-up	Within 1 week of follow-up visit
Form 7	Recurrence / Progression	Within 24 hours of detection
Form 8a	Recurrence Local Pathology	Within 2 weeks or surgery
Form 8a	Recurrence Review Pathology	Not specified
Form 9	Death	Within 24 hours of detection

APPENDIX 7
TOENAIL COLLECTION PROTOCOL

TO BE PRINTED ON HOSPITAL LETTERHEADED PAPER.

<Patient Title> <Patient Surname>
<Patient Address 1>
<Patient Address 2>
<Patient Address 3>
<Patient Address 4>
<Patient Address 5>
<Patient Post Code>

Dear <Patient Title> <Patient Surname>

RE: Bladder Cancer Prognosis Programme – toenail collection request

We would like to thank you once again for taking part in the Bladder Cancer Prognosis Programme. As explained by the research nurse we have asked you to collect samples of your toenails and bring them back to us at your next hospital visit.

Why use toenails?
Many of the minerals that you need for good health find their way from your diet into parts of your body such as your hair and nails. Nails can therefore provide a painless and easy way of investigating the levels of these various natural minerals contained within the body. Toenails are usually covered up and are therefore less exposed to chemicals and other substances than hair or fingernails.

As part of the Bladder Cancer Prognosis Programme we would like to collect clippings from **all of your toenails**, so that we can look at the levels of these minerals amongst people such as yourself who have bladder cancer or a bladder abnormality. The toenail samples will be used for the purpose of current and future laboratory research.

Collection of your toenails for the Bladder Cancer Prognosis Programme
We would like you to read the attached instructions carefully, then to follow the instructions step by step. Please **do not** collect any nail clippings if your GP has advised you not to cut your own nails, if you are receiving treatment for the inflammation of blood vessels, if you are taking medication for high blood pressure (eg. Warfarin) or if you are a diabetic.

If you have any queries or concerns, please telephone the research nurse on <research nurse mobile>.

Once you have collected the toenails, please bring them back to the research nurse at the hospital, when you attend for your next appointment on <date>.

Thank you again for your continued participation.

Yours sincerely

<Urologist>

SPECIMEN NO: ☐☐☐☐☐

BLADDER CANCER PROGNOSIS PROGRAMME

Cancer Research UK Bladder Cancer Group

Toenail Collection Instruction Leaflet

FINAL VERSION 1.0 November 2005

PLEASE DESTROY PREVIOUS VERSIONS

MREC APPROVAL:
START DATE:

A programme of research

Conducted by: UNIVERSITY OF BIRMINGHAM

Funded by: CANCER RESEARCH UK

TOENAIL COLLECTION INSTRUCTIONS

Please read and carefully follow the instructions below. You may need to ask a family member or friend to help you.

First of all, please answer the following questions by circling either Yes or No.

1. Are you currently receiving treatment for inflamed blood vessels?	**YES / NO**
2. Are you taking tablets to thin your blood (e.g. Warfarin)?	**YES / NO**
3. Are you a diabetic?	**YES / NO**
4. Has your GP advised you not to cut your nails (for any reason)?	**YES / NO**

If you have answered **YES** to **any** of the above questions, **do not** collect any nail clippings and tell your research nurse at your next appointment.

If you have answered **NO** to **all** of the above questions, collect your toenails by following the instructions below;

Instructions

1. The toenail clippings should be cut immediately after bathing or showering using nail clippers or nail scissors routinely used for that purpose. Any nail polish on the nails should be removed with nail polish remover prior to bathing or showering.
2. After bathing or showering, take care to thoroughly rinse your feet to remove soap and shampoo from the nails. Use a soft nail brush to remove any debris from under the nails. Dry the nails with a tissue or towel.
3. Using a clean pair of nail scissors or nail clippers, carefully trim the nail straight across, to a comfortable length, so that the nail is level with the ends of the toes.
4. **DO NOT** cut down the sides of the nail. File the edges of the toenails smooth with an emery board.
5. Ensure, where possible, that you have taken clippings from every toe.
6. If you are **UNABLE** to obtain toenail clippings or if your toenails are not long enough to clip, you can alternatively collect all of your fingernail clippings in the same way as described above. Fingernails should only be collected where toenail collection is impossible.

Please telephone the research nurse on <Research Nurse mobile> **if you are unable to collect either toenail or fingernail clippings for any reason.**

(Continues on next page)

TOENAIL COLLECTION INSTRUCTIONS (Continued)

Instructions *(continued from previous page)*

7. Put the toenail clippings into the plastic bag provided and seal the bag.
8. Tick the boxes on the diagram page to indicate which toes (or fingers) you have taken clippings from
9. Fold up this instruction leaflet by folding it in half, then in half again.
10. Put the plastic envelope and the folded instruction leaflet into the paper envelope.
11. Peel off the paper backing of the self adhesive strip and stick down the envelope flap.
12. Write the date that you cut your nails on the front of the paper envelope in the space provided

Bring the envelope back to the research nurse at the hospital, when you attend for your next appointment.

CHECK LIST

- Check that it is OK for you to clip your toenails by answering the first 4 questions on the toenail collection instructions page

If you answered **YES** to **any** of the questions - do not collect any nail samples and tell your research nurse at your next appointment.

If you answered **NO** to **all** of the questions;

- collect the toenail samples according to the instructions above
- place all of the toenail clippings into the plastic bag and seal
- indicate on the diagram overleaf which nail clippings have been collected
- Put the folded instruction leaflet and the sealed plastic bag containing your nail clippings inside the paper envelope.
- Write the date that the nails were clipped on the paper envelope in the space provided
- Bring the paper envelope to the research nurse at the hospital on your next visit.

**DIAGRAM PAGE**	**Page 3**

Please indicate by ticking the boxes on the diagram below, which toes you have taken clippings from.

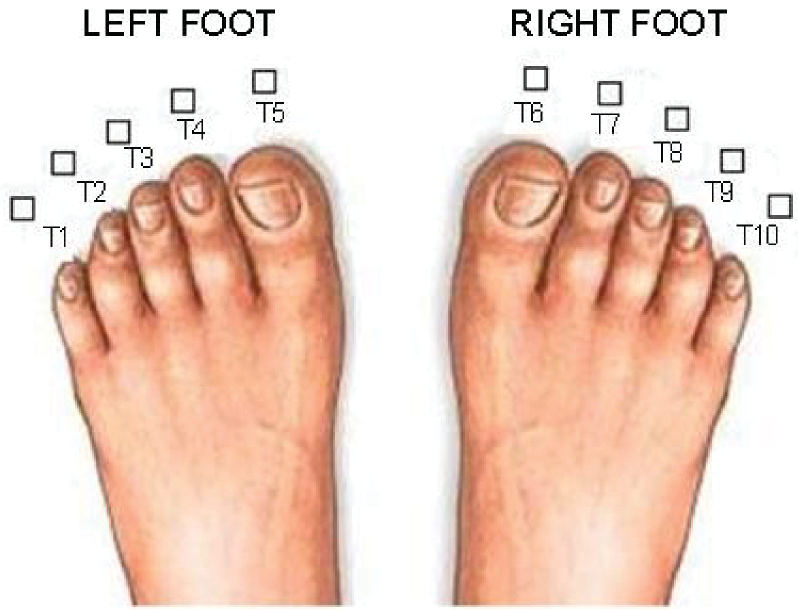

If you have not been able to collect toenail clippings, please indicate on the diagram below, which fingers you have taken clippings from.

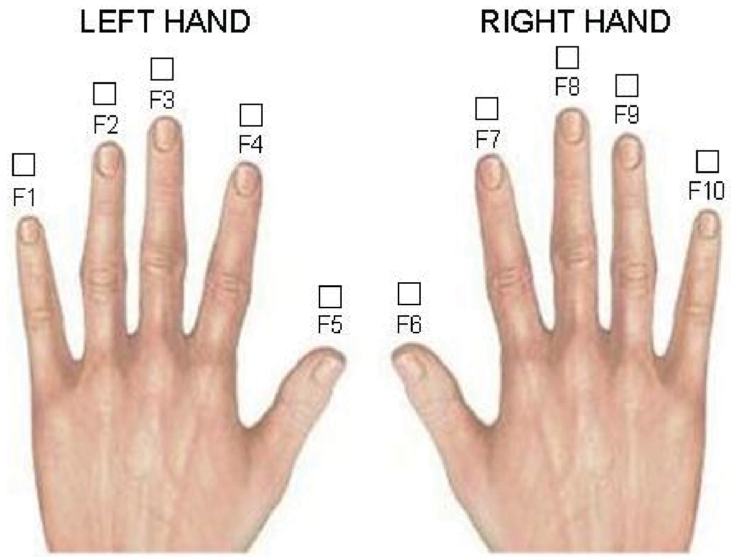

Please fold up this instruction leaflet. Put the folded leaflet and the plastic bag containing your nail clippings into the paper envelope provided. Seal the paper envelope and return it to the research nurse on your next hospital visit.

APPENDIX 8
DECLARATION OF HELSINKI

DECLARATION OF HELSINKI (SOUTH AFRICA REVISION 1996)

HUMAN EXPERIMENTATION

In 1964, the World Medical Association drew up a code of ethics on human experimentation. This code, known as the Declaration of Helsinki, as amended by the 29th World Medical Assembly, Tokyo, Japan in 1975, the 35th World Medical Assembly, Venice, Italy, in 1983 and the 41st World Medical Assembly, Hong Kong in 1989 and the 48th General Assembly, South Africa in 1996 reads:

It is the mission of the medical doctor to safeguard the health of the people. His or her knowledge and conscience are dedicated to the fulfilment of this mission.

The Declaration of Geneva of the World Medical Association binds the physician with the words, "The health of my patient will be my first consideration", and the International Code of Medical Ethics declares that "A physician shall act only in the patient's interest when providing medical care which might have the effect of weakening the physical and mental condition of the patient."

The purpose of biomedical research involving human subjects must be to improve diagnostic, therapeutic and prophylactic procedures and the understanding of the aetiology and pathogenesis of disease.

In current medical practice most diagnostic, therapeutic and prophylactic procedures involve hazards. This applies especially to biomedical research.

Medical Progress is based on research which ultimately must rest in part on experimentation involving human subjects.

In the fields of biomedical research a fundamental distinction must be recognised between medical research in which the aim is essentially diagnostic or therapeutic for a patient, and medical research, the essential object of which is purely scientific and without implying direct diagnostic or therapeutic value to the person subjected to the research.

Special caution must be exercised in the conduct of research which may affect the environment, and the welfare of animals used for research must be respected.

Because it is essential that the results of the laboratory experiments be applied to human beings to further scientific knowledge and to help suffering humanity, the World Medical Association has prepared the following recommendations as a guide to every physician in biomedical research involving human subjects. They should be kept under review in the future. It must be stressed that the standards as drafted are only a guide to physicians all over the world. Physicians are not relieved from criminal, civil or ethical responsibilities under the laws of their own country.

1. Basic principles

(1) Biomedical research involving human subjects must conform to generally accepted scientific principles and should be based on adequately performed laboratory and animal experimentation and a thorough knowledge of the scientific literature.

(2) The design and performance of each procedure involving human subjects should be clearly formulated in an experimental protocol which should be transmitted for consideration, comment and guidance to a specially appointed committee independent of the investigator and the sponsor provided that this independent committee is in conformity with the laws and regulations of the country in which the research experiment is performed.

(3) Biomedical research involving human subjects should be conducted only by scientifically qualified persons and under the supervision of a clinically competent medical person. The responsibility for the human subject must always rest with the medically qualified person and never rest on the subject of the research, even though the subject has given his or her consent.

(4) Biomedical research involving human subjects cannot legitimately be carried out unless the importance of the objective is in proportion to the inherent risk to the subject.

(5) Every biomedical research project involving human subjects should be preceded by careful assessment of predictable risk in comparison with foreseeable benefits to the subject or to others. Concern for the interests of the subject must always prevail over the interests of science and society.

(6) The right of the research subject to safeguard his or her integrity must always be respected. Every precaution should be taken to respect the privacy of the subject and to minimise the impact of the trial on the subject's physical and mental integrity and on the personality of the subject.

(7) Physicians should abstain from engaging in research projects involving human subjects unless they are satisfied that the hazards involved are believed to be predictable. Physicians should cease any investigation if the hazards are found to outweigh the potential benefits.

(8) In publication of the results of his or her research, the physician is obliged to preserve the accuracy of the results. Reports of experimentation not in accordance with the principles laid down in this Declaration should not be accepted for publication.

(9) In any research on human beings, each potential subject must be adequately informed of the aims, methods, anticipated benefits and potential hazards of the trial and the discomfort it may entail. He or she should be informed that he or she is at liberty to abstain from participation in the trial and that he or she is free to withdraw his or her consent to participation at any time. The physician should then obtain the subject's freely given informed consent, preferably in writing.

(10) When obtaining informed consent for the research project the physician should be particularly cautious if the subject is in a dependent relationship to him or her or may consent under duress. In that case the informed consent should be obtained by a physician who is not engaged in the investigation and who is completely independent of this official relationship.

(11) In case of legal incompetence, informed consent should be obtained from the legal guardian in accordance with national legislation. Where physical or mental incapacity makes it impossible to obtain informed consent, or when the subject is a minor, permission from the responsible relative replaces that of the subject in accordance with national legislation.

Whenever the minor child is in fact able to give a consent, the minor's consent must be obtained in addition to the consent the minor's legal guardian.

(12) The research protocol should always contain a statement of the ethical considerations involved and should indicate that the principles enunciated in the present Declaration are complied with.

II Medical Research combined with professional care (Clinical research)

(1) In the treatment of the sick person, the physician must be free to use a new diagnostic and therapeutic measure, if in his or her judgement it offers hope of saving life, reestablishing health or alleviating suffering.

(2) The potential benefits, hazards and discomfort of a new method should be weighed against the advantages of the best current diagnostic and therapeutic method.

In any medical trial, every patient - including those of a control group, if any - should be assured of the best-proven diagnostic and therapeutic method. This does not exclude the use of inert placebo in studies where no proven diagnostic or therapeutic method exists.

(4) The refusal of the patient to participate in a trial must never interfere with the physician-patient relationship.

(5) If the physician considers it essential not to obtain informed consent, the specific reasons for this proposal should be stated in the experimental protocol for transmission to the independent committee (1.2).

(6) The physician can combine medical research with professional care, the objective being the acquisition of new medical knowledge, only to the extent that medical research is justified by its potential diagnostic or therapeutic value for the patient.

III Non-therapeutic biomedical research involving human subjects (Non-clinical biomedical research)

(1) In the purely scientific application of medical research carried out on a human being, it is the duty of the physician to remain the protector of the life and health of that person on whom biomedical research is being carried out.

(2) The subjects should be volunteers - either healthy persons or patients for whom the experimental design is not related to the patient's illness.

(3) The investigator or the investigating team should discontinue the research if in his/her or their judgement it may, if continued, be harmful to the individual.

(4) In research on man, the interest of science and society should never take precedence over considerations related to the well being of the patient.

APPENDIX 9
REFERENCES

Reference List

(1) ONS. Cancer statistics – registrations of cancer diagnosed in 2000. England. London: ONS, 2003.

(2) Steward BW KP. World Cancer Report. Lyon: WHO-IARC; 2003.

(3) Morrison AS, Proppe KH, Verhoek WG, Aoki K, Leck I, Ohno Y et al. Histologic features of bladder cancer in Boston, USA, Manchester, UK, and Nagoya, Japan. Int J Cancer 1982; 30(6):701-705.

(4) Eble JN, Young RH. Carcinoma of the urinary bladder: a review of its diverse morphology. Semin Diagn Pathol 1997; 14(2):98-108.

(5) Gephardt GN, Baker PB. Interinstitutional comparison of bladder carcinoma surgical pathology report adequacy. A College of American Pathologists Q-Probes Study of 7234 bladder biopsies and curettings in 268 institutions. Arch Pathol Lab Med 1995; 119(8):681-685.

(6) British Association of Urological Surgeons Section of Oncology audit data (unpublished). London: BAUS; 2000.

(7) Wallace DM, Bryan RT, Dunn JA, Begum G, Bathers S. Delay and survival in bladder cancer. BJU Int 2002; 89(9):868-878.

(8) Tolley DA, Parmar MK, Grigor KM, Lallemand G, Benyon LL, Fellows J et al. The effect of intravesical mitomycin C on recurrence of newly diagnosed superficial bladder cancer: a further report with 7 years of follow up. J Urol 1996; 155(4):1233-1238.

(9) Solsona E, Iborra I, Ricos JV, Monros JL, Casanova J, Dumont R. Effectiveness of a single immediate mitomycin C instillation in patients with low risk superficial bladder cancer: short and long-term followup. J Urol 1999; 161(4):1120-1123.

(10) Hinotsu S, Akaza H, Ohashi Y, Kotake T. Intravesical chemotherapy for maximum prophylaxis of new early phase superficial bladder carcinoma treated by transurethral resection: a combined analysis of trials by the Japanese Urological Cancer Research Group using smoothed hazard function. Cancer 1999; 86(9):1818-1826.

(11) Sylvester RJ, van der MEIJDEN AP, Lamm DL. Intravesical bacillus Calmette-Guerin reduces the risk of progression in patients with superficial bladder cancer: a meta-analysis of the published results of randomized clinical trials. J Urol 2002; 168(5):1964-1970.

(12) Bryan RT ea. Molecular pathways in bladder cancer. BJUI. In press 2004.

(13) Botteman MF, Pashos CL, Redaelli A, Laskin B, Hauser R. The health economics of bladder cancer: a comprehensive review of the published literature. Pharmacoeconomics 2003; 21(18):1315-1330.

(14) Riley GF, Potosky AL, Lubitz JD, Kessler LG. Medicare payments from diagnosis to death for elderly cancer patients by stage at diagnosis. Med Care 1995; 33(8):828-841.

(15) Silverman DT ea. Cancer epidemiology and prevention. In: Fraumeni JF, editor. New York: Oxford university press, 1996. 156-179.

(16) Ross RK, Jones PA, Yu MC. Bladder cancer epidemiology and pathogenesis. Semin Oncol 1996; 23(5):536-545.

(17) Zeegers MP, Tan FE, Dorant E, van den Brandt PA. The impact of characteristics of cigarette smoking on urinary tract cancer risk: a meta-analysis of epidemiologic studies. Cancer 2000; 89(3):630-639.

(18) Zeegers MP, Goldbohm RA, van den Brandt PA. A prospective study on active and environmental tobacco smoking and bladder cancer risk (The Netherlands). Cancer Causes Control 2002; 13(1):83-90.

(19) Sorahan T, Lancashire RJ, Sole G. Urothelial cancer and cigarette smoking: findings from a regional case-controlled study. Br J Urol 1994; 74(6):753-756.

(20) Nascimento Cea. 2005.
Ref Type: Unpublished Work

(21) La Vecchia C, Negri E. Nutrition and bladder cancer. Cancer Causes Control 1996; 7(1):95-100.

(22) Steinmaus CM, Nunez S, Smith AH. Diet and bladder cancer: a meta-analysis of six dietary variables. Am J Epidemiol 2000; 151(7):693-702.

(23) Steinmaus CM, Nunez S, Smith AH. Diet and bladder cancer: a meta-analysis of six dietary variables. Am J Epidemiol 2000; 151(7):693-702.

(24) Augustsson K, Skog K, Jagerstad M, Dickman PW, Steineck G. Dietary heterocyclic amines and cancer of the colon, rectum, bladder, and kidney: a population-based study. Lancet 1999; 353(9154):703-707.

(25) Helzlsouer KJ, Comstock GW, Morris JS. Selenium, lycopene, alpha-tocopherol, beta-carotene, retinol, and subsequent bladder cancer. Cancer Res 1989; 49(21):6144-6148.

(26) Garland M, Morris JS, Stampfer MJ, Colditz GA, Spate VL, Baskett CK et al. Prospective study of toenail selenium levels and cancer among women. J Natl Cancer Inst 1995; 87(7):497-505.

(27) Nomura A, Heilbrun LK, Morris JS, Stemmermann GN. Serum selenium and the risk of cancer, by specific sites: case-control analysis of prospective data. J Natl Cancer Inst 1987; 79(1):103-108.

(28) Knekt P, Aromaa A, Maatela J, Alfthan G, Aaran RK, Hakama M et al. Serum selenium and subsequent risk of cancer among Finnish men and women. J Natl Cancer Inst 1990; 82(10):864-868.

(29) Zeegers MP, Goldbohm RA, Bode P, van den Brandt PA. Prediagnostic toenail selenium and risk of bladder cancer. Cancer Epidemiol Biomarkers Prev 2002; 11(11):1292-1297.

(30) Michaud DS, Hartman TJ, Taylor PR, Pietinen P, Alfthan G, Virtamo J et al. No Association between toenail selenium levels and bladder cancer risk. Cancer Epidemiol Biomarkers Prev 2002; 11(11):1505-1506.

(31) Zeegers MP, Kellen E, Buntinx F, van den Brandt PA. The association between smoking, beverage consumption, diet and bladder cancer: a systematic literature review. World J Urol 2004; 21(6):392-401.

(32) Zeegers MP, Goldbohm RA, Bode P, van den Brandt PA. Prediagnostic toenail selenium and risk of bladder cancer. Cancer Epidemiol Biomarkers Prev 2002; 11(11):1292-1297.

(33) Helzlsouer KJ, Comstock GW, Morris JS. Selenium, lycopene, alpha-tocopherol, beta-carotene, retinol, and subsequent bladder cancer. Cancer Res 1989; 49(21):6144-6148.

(34) Garland M, Morris JS, Stampfer MJ, Colditz GA, Spate VL, Baskett CK et al. Prospective study of toenail selenium levels and cancer among women. J Natl Cancer Inst 1995; 87(7):497-505.

(35) Nomura A, Heilbrun LK, Morris JS, Stemmermann GN. Serum selenium and the risk of cancer, by specific sites: case-control analysis of prospective data. J Natl Cancer Inst 1987; 79(1):103-108.

(36) Knekt P, Aromaa A, Maatela J, Alfthan G, Aaran RK, Hakama M et al. Serum selenium and subsequent risk of cancer among Finnish men and women. J Natl Cancer Inst 1990; 82(10):864-868.

(37) Helzlsouer KJ, Comstock GW, Morris JS. Selenium, lycopene, alpha-tocopherol, beta-carotene, retinol, and subsequent bladder cancer. Cancer Res 1989; 49(21):6144-6148.

(38) Garland M, Morris JS, Stampfer MJ, Colditz GA, Spate VL, Baskett CK et al. Prospective study of toenail selenium levels and cancer among women. J Natl Cancer Inst 1995; 87(7):497-505.

(39) Nomura A, Heilbrun LK, Morris JS, Stemmermann GN. Serum selenium and the risk of cancer, by specific sites: case-control analysis of prospective data. J Natl Cancer Inst 1987; 79(1):103-108.

(40) Knekt P, Aromaa A, Maatela J, Alfthan G, Aaran RK, Hakama M et al. Serum selenium and subsequent risk of cancer among Finnish men and women. J Natl Cancer Inst 1990; 82(10):864-868.

(41) Steinmaus CM, Nunez S, Smith AH. Diet and bladder cancer: a meta-analysis of six dietary variables. Am J Epidemiol 2000; 151(7):693-702.

(42) Michaud DS, Spiegelman D, Clinton SK, Rimm EB, Willett WC, Giovannucci E. Prospective study of dietary supplements, macronutrients, micronutrients, and risk of bladder cancer in US men. Am J Epidemiol 2000; 152(12):1145-1153.

(43) Hafner C eal. Evidence for oligoclonality and tumor spread by intraluminal seeding in multifocal urothelial carcinomas of the upper and lower urinary tract. Oncogene 2001;(20):4910-5].

(44) Hartmann A, Rosner U, Schlake G, Dietmaier W, Zaak D, Hofstaedter F et al. Clonality and genetic divergence in multifocal low-grade superficial urothelial carcinoma as determined by chromosome 9 and p53 deletion analysis. Lab Invest 2000; 80(5):709-718.

(45) Michaud DS, Spiegelman D, Clinton SK, Rimm EB, Curhan GC, Willett WC et al. Fluid intake and the risk of bladder cancer in men. N Engl J Med 1999; 340(18):1390-1397.

(46) Zeegers MP, Dorant E, Goldbohm RA, van den Brandt PA. Are coffee, tea, and total fluid consumption associated with bladder cancer risk? Results from the Netherlands Cohort Study. Cancer Causes Control 2001; 12(3):231-238.

(47) Mills PK, Beeson WL, Phillips RL, Fraser GE. Bladder cancer in a low risk population: results from the Adventist Health Study. Am J Epidemiol 1991; 133(3):230-239.

(48) Donat SM, Bayuga S, Herr HW, Berwick M. Fluid intake and the risk of tumor recurrence in patients with superficial bladder cancer. J Urol 2003; 170(5):1777-1780.

(49) Zeegers MP, Volovics A, Dorant E, Goldbohm RA, van den Brandt PA. Alcohol consumption and bladder cancer risk: results from The Netherlands Cohort Study. Am J Epidemiol 2001; 153(1):38-41.

(50) IARC Monogr Eval Carcinog Risks Hum. Coffee, tea, mate, methylxanthines and methylglioxal. Lyon: IARC; 1991.

(51) Zeegers MP, Dorant E, Goldbohm RA, van den Brandt PA. Are coffee, tea, and total fluid consumption associated with bladder cancer risk? Results from the Netherlands Cohort Study. Cancer Causes Control 2001; 12(3):231-238.

(52) Zeegers MP, Dorant E, Goldbohm RA, van den Brandt PA. Are coffee, tea, and total fluid consumption associated with bladder cancer risk? Results from the Netherlands Cohort Study. Cancer Causes Control 2001; 12(3):231-238.

(53) Weihrauch MR, Diehl V. Artificial sweeteners--do they bear a carcinogenic risk? Ann Oncol 2004; 15(10):1460-1465.

(54) Takayama S, Renwick AG, Johansson SL, Thorgeirsson UP, Tsutsumi M, Dalgard DW et al. Long-term toxicity and carcinogenicity study of cyclamate in nonhuman primates. Toxicol Sci 2000; 53(1):33-39.

(55) Lindley MG. New developments in low-calorie sweeteners. World Rev Nutr Diet 1999; 85:44-51.:44-51.

(56) International Agency for Research on Cancer. Some chemicals that cause tumors of the kidney or urinary in rodents and some other substances. IARC monographs on the evaluation of carcinogenic risks to humans 1999; 73.

(57) Pommer W, Bronder E, Klimpel A, Helmert U, Greiser E, Molzahn M. Urothelial cancer at different tumour sites: role of smoking and habitual intake of analgesics and laxatives. Results of the Berlin Urothelial Cancer Study. Nephrol Dial Transplant 1999; 14(12):2892-2897.

(58) Castelao JE, Yuan JM, Gago-Dominguez M, Yu MC, Ross RK. Non-steroidal anti-inflammatory drugs and bladder cancer prevention. Br J Cancer 2000; 82(7):1364-1369.

(59) Piper JM, Tonascia J, Matanoski GM. Heavy phenacetin use and bladder cancer in women aged 20 to 49 years. N Engl J Med 1985; 313(5):292-295.

(60) Overall evaluations of carcinogenicity: an updating of IARC Monographs volumes 1 to 42. IARC Monogr Eval Carcinog Risks Hum Suppl 1987; 7:1-440.:1-440.

(61) Rosenberg L, Rao RS, Palmer JR, Strom BL, Zauber A, Warshauer ME et al. Transitional cell cancer of the urinary tract and renal cell cancer in relation to acetaminophen use (United States). Cancer Causes Control 1998; 9(1):83-88.

(62) Derby LE, Jick H. Acetaminophen and renal and bladder cancer. Epidemiology 1996; 7(4):358-362.

(63) Piper JM, Tonascia J, Matanoski GM. Heavy phenacetin use and bladder cancer in women aged 20 to 49 years. N Engl J Med 1985; 313(5):292-295.

(64) Steineck G, Wiholm BE, Gerhardsson d, V. Acetaminophen, some other drugs, some diseases and the risk of transitional cell carcinoma. A population-based case-control study. Acta Oncol 1995; 34(6):741-748.

(65) Some chemicals that cause tumors of the kidney or urinary bladder in rodents and some other substances. IARC Monogr Eval Carcinog Risks Hum 1999; 73.

(66) Pommer W, Bronder E, Klimpel A, Helmert U, Greiser E, Molzahn M. Urothelial cancer at different tumour sites: role of smoking and habitual intake of analgesics and laxatives. Results of the Berlin Urothelial Cancer Study. Nephrol Dial Transplant 1999; 14(12):2892-2897.

(67) Kaye JA, Myers MW, Jick H. Acetaminophen and the risk of renal and bladder cancer in the general practice research database. Epidemiology 2001; 12(6):690-694.

(68) Friis S, Nielsen GL, Mellemkjaer L, McLaughlin JK, Thulstrup AM, Blot WJ et al. Cancer risk in persons receiving prescriptions for paracetamol: a Danish cohort study. Int J Cancer 2002; 97(1):96-101.

(69) Castelao JE, Yuan JM, Gago-Dominguez M, Yu MC, Ross RK. Non-steroidal anti-inflammatory drugs and bladder cancer prevention. Br J Cancer 2000; 82(7):1364-1369.

(70) Castelao JE, Yuan JM, Gago-Dominguez M, Yu MC, Ross RK. Non-steroidal anti-inflammatory drugs and bladder cancer prevention. Br J Cancer 2000; 82(7):1364-1369.

(71) Sorensen HT, Friis S, Norgard B, Mellemkjaer L, Blot WJ, McLaughlin JK et al. Risk of cancer in a large cohort of nonaspirin NSAID users: a population-based study. Br J Cancer 2003; 88(11):1687-1692.

(72) Langman MJ, Cheng KK, Gilman EA, Lancashire RJ. Effect of anti-inflammatory drugs on overall risk of common cancer: case-control study in general practice research database. BMJ 2000; 320(7250):1642-1646.

(73) Schreinemachers DM, Everson RB. Aspirin use and lung, colon, and breast cancer incidence in a prospective study. Epidemiology 1994; 5(2):138-146.

(74) Kaye JA, Myers MW, Jick H. Acetaminophen and the risk of renal and bladder cancer in the general practice research database. Epidemiology 2001; 12(6):690-694.

(75) Pommer W, Bronder E, Klimpel A, Helmert U, Greiser E, Molzahn M. Urothelial cancer at different tumour sites: role of smoking and habitual intake of analgesics and laxatives. Results of the Berlin Urothelial Cancer Study. Nephrol Dial Transplant 1999; 14(12):2892-2897.

(76) Knight A, Askling J, Ekbom A. Cancer incidence in a population-based cohort of patients with Wegener's granulomatosis. Int J Cancer 2002; 100(1):82-85.

(77) Travis LB, Curtis RE, Glimelius B, Holowaty EJ, Van Leeuwen FE, Lynch CF et al. Bladder and kidney cancer following cyclophosphamide therapy for non-Hodgkin's lymphoma. J Natl Cancer Inst 1995; 87(7):524-530.

(78) Pedersen-Bjergaard J, Ersboll J, Hansen VL, Sorensen BL, Christoffersen K, Hou-Jensen K et al. Carcinoma of the urinary bladder after treatment with cyclophosphamide for non-Hodgkin's lymphoma. N Engl J Med 1988; 318(16):1028-1032.

(79) Overall evaluations of carcinogenicity: an updating of IARC Monographs volumes 1 to 42. IARC Monogr Eval Carcinog Risks Hum Suppl 1987; 7:1-440.:1-440.

(80) Overall evaluations of carcinogenicity: an updating of IARC Monographs volumes 1 to 42. IARC Monogr Eval Carcinog Risks Hum Suppl 1987; 7:1-440.:1-440.

(81) Golka K, Wiese A, Assennato G, Bolt HM. Occupational exposure and urological cancer. World J Urol 2004; 21(6):382-391.

(82) Boffetta P, Jourenkova N, Gustavsson P. Cancer risk from occupational and environmental exposure to polycyclic aromatic hydrocarbons. Cancer Causes Control 1997; 8(3):444-472.

(83) Boffetta P, Silverman DT. A meta-analysis of bladder cancer and diesel exhaust exposure. Epidemiology 2001; 12(1):125-130.

(84) Kogevinas M, 't MA, Cordier S, Ranft U, Gonzalez CA, Vineis P et al. Occupation and bladder cancer among men in Western Europe. Cancer Causes Control 2003; 14(10):907-914.

(85) Cantor KP, Lynch CF, Hildesheim ME, Dosemeci M, Lubin J, Alavanja M et al. Drinking water source and chlorination byproducts. I. Risk of bladder cancer. Epidemiology 1998; 9(1):21-28.

(86) King WD, Marrett LD. Case-control study of bladder cancer and chlorination by-products in treated water (Ontario, Canada). Cancer Causes Control 1996; 7(6):596-604.

(87) McGeehin MA, Reif JS, Becher JC, Mangione EJ. Case-control study of bladder cancer and water disinfection methods in Colorado. Am J Epidemiol 1993; 138(7):492-501.

(88) Cantor KP, Hoover R, Hartge P, Mason TJ, Silverman DT, Altman R et al. Bladder cancer, drinking water source, and tap water consumption: a case-control study. J Natl Cancer Inst 1987; 79(6):1269-1279.

(89) Wilkins JR, III, Comstock GW. Source of drinking water at home and site-specific cancer incidence in Washington County, Maryland. Am J Epidemiol 1981; 114(2):178-190.

(90) Chen YC, Su HJ, Guo YL, Hsueh YM, Smith TJ, Ryan LM et al. Arsenic methylation and bladder cancer risk in Taiwan. Cancer Causes Control 2003; 14(4):303-310.

(91) Chiou HY, Chiou ST, Hsu YH, Chou YL, Tseng CH, Wei ML et al. Incidence of transitional cell carcinoma and arsenic in drinking water: a follow-up study of 8,102 residents in an arseniasis-endemic area in northeastern Taiwan. Am J Epidemiol 2001; 153(5):411-418.

(92) Smith AH, Goycolea M, Haque R, Biggs ML. Marked increase in bladder and lung cancer mortality in a region of Northern Chile due to arsenic in drinking water. Am J Epidemiol 1998; 147(7):660-669.

(93) Hopenhayn-Rich C, Biggs ML, Smith AH. Lung and kidney cancer mortality associated with arsenic in drinking water in Cordoba, Argentina. Int J Epidemiol 1998; 27(4):561-569.

(94) Some drinking-water disinfectants and contaminants, including arsenic. IARC Monogr Eval Carcinog Risks Hum 2004; 84:1-477.:1-477.

(95) McGeehin MA, Reif JS, Becher JC, Mangione EJ. Case-control study of bladder cancer and water disinfection methods in Colorado. Am J Epidemiol 1993; 138(7):492-501.

(96) Ward MH, Cantor KP, Riley D, Merkle S, Lynch CF. Nitrate in public water supplies and risk of bladder cancer. Epidemiology 2003; 14(2):183-190.

(97) Brenner DJ, Curtis RE, Hall EJ, Ron E. Second malignancies in prostate carcinoma patients after radiotherapy compared with surgery. Cancer 2000; 88(2):398-406.

(98) Neugut AI, Ahsan H, Robinson E, Ennis RD. Bladder carcinoma and other second malignancies after radiotherapy for prostate carcinoma. Cancer 1997; 79(8):1600-1604.

(99) Inskip PD, Monson RR, Wagoner JK, Stovall M, Davis FG, Kleinerman RA et al. Cancer mortality following radium treatment for uterine bleeding. Radiat Res 1990; 123(3):331-344.

(100) Boice JD, Jr., Engholm G, Kleinerman RA, Blettner M, Stovall M, Lisco H et al. Radiation dose and second cancer risk in patients treated for cancer of the cervix. Radiat Res 1988; 116(1):3-55.

(101) Edmonds CJ, Smith T. The long-term hazards of the treatment of thyroid cancer with radioiodine. Br J Radiol 1986; 59(697):45-51.

(102) Piper JM, Matanoski GM, Tonascia J. Bladder cancer in young women. Am J Epidemiol 1986; 123(6):1033-1042.

(103) Franklyn JA, Maisonneuve P, Sheppard M, Betteridge J, Boyle P. Cancer incidence and mortality after radioiodine treatment for hyperthyroidism: a population-based cohort study. Lancet 1999; %19;353(9170):2111-2115.

(104) Rothman N, Bhatnagar VK, Hayes RB, Zenser TV, Kashyap SK, Butler MA et al. The impact of interindividual variation in NAT2 activity on benzidine urinary metabolites and urothelial DNA adducts in exposed workers. Proc Natl Acad Sci U S A 1996; 93(10):5084-5089.

(105) Wada S, Yoshimura R, Masuda C, Hase T, Ikemoto S, Kishimoto T et al. Are tobacco use and urine pH indicated as risk factors for bladder carcinoma? Int J Urol 2001; 8(3):106-109.

(106) Hartge P, Harvey EB, Linehan WM, Silverman DT, Sullivan JW, Hoover RN et al. Unexplained excess risk of bladder cancer in men. J Natl Cancer Inst 1990; 82(20):1636-1640.

(107) Cantor KP, Lynch CF, Johnson D. Bladder cancer, parity, and age at first birth. Cancer Causes Control 1992; 3(1):57-62.

(108) Pelucchi C, La Vecchia C, Negri E, Dal Maso L, Franceschi S. Smoking and other risk factors for bladder cancer in women. Prev Med 2002; 35(2):114-120.

(109) Pelucchi C, La Vecchia C, Negri E, Dal Maso L, Franceschi S. Smoking and other risk factors for bladder cancer in women. Prev Med 2002; 35(2):114-120.

(110) Mahboubi AO, Ahlvin RC, Mahboubi EO. Familial aggregation of urothelial carcinoma. J Urol 1981; 126(5):691-692.

(111) Sharma SK, Bapna BC, Singh SM. Familial profile of transitional cell carcinoma. Br J Urol 1976; 48(6):442.

(112) McCullough DL, Lamma DL, McLaughlin AP, III, Gittes RF. Familial transitional cell carcinoma of the bladder. J Urol 1975; 113(5):629-635.

(113) Fraumeni Jr JF TL. Malignant bladder tumors in a man and his three sons. J Am Med Assoc 1967; 201(507).

(114) Kantor AF, Hartge P, Hoover RN, Fraumeni JF, Jr. Familial and environmental interactions in bladder cancer risk. Int J Cancer 1985; 35(6):703-706.

(115) Kantor AF, Hartge P, Hoover RN, Fraumeni JF, Jr. Familial and environmental interactions in bladder cancer risk. Int J Cancer 1985; 35(6):703-706.

(116) Howe GR, Burch JD, Miller AB, Cook GM, Esteve J, Morrison B et al. Tobacco use, occupation, coffee, various nutrients, and bladder cancer. J Natl Cancer Inst 1980; 64(4):701-713.

(117) Nomura A, Kolonel LN, Yoshizawa CN. Smoking, alcohol, occupation, and hair dye use in cancer of the lower urinary tract. Am J Epidemiol 1989; 130(6):1159-1163.

(118) Claude J, Kunze E, Frentzel-Beyme R, Paczkowski K, Schneider J, Schubert H. Life-style and occupational risk factors in cancer of the lower urinary tract. Am J Epidemiol 1986; 124(4):578-589.

(119) Hartge P, Hoover R, Altman R, Austin DF, Cantor KP, Child MA et al. Use of hair dyes and risk of bladder cancer. Cancer Res 1982; 42(11):4784-4787.

(120) Henley SJ, Thun MJ. Use of permanent hair dyes and bladder-cancer risk. Int J Cancer 2001; 94(6):903-906.

(121) Hennekens CH, Speizer FE, Rosner B, Bain CJ, Belanger C, Peto R. Use of permanent hair dyes and cancer among registered nurses. Lancet 1979; 1(8131):1390-1393.

(122) La VC, Tavani A. Epidemiological evidence on hair dyes and the risk of cancer in humans. Eur J Cancer Prev 1995; 4(1):31-43.

(123) La VC, Tavani A. Epidemiological evidence on hair dyes and the risk of cancer in humans. Eur J Cancer Prev 1995; 4(1):31-43.

(124) Gago-Dominguez M, Castelao JE, Yuan JM, Yu MC, Ross RK. Use of permanent hair dyes and bladder-cancer risk. Int J Cancer 2001; 91(4):575-579.

(125) Bryan RT, Wallace DM. 'Superficial' bladder cancer - time to uncouple pT1 tumours from pTa tumours. BJU Int 2002; 90(9):846-852.

(126) Cox D. Regression models and life-tables. J R Statist Soc B 1972; 34:187-220.

(127) Statistical, Software. Release 8.0 [College Station, Texas: STATA Corporation; 2003.

(128) Schoenfeld D. Partial residuals for the proportional hazards regression model. Biometrika 1982; 69:239-241.

(129) Agresti A. An Introduction to categorical data analysis. John Wiley and sons.; 1996.

(130) Jolly K, Bradley F, Sharp S, Smith H, Thompson S, Kinmonth AL et al. Randomised controlled trial of follow up care in general practice of patients with myocardial infarction and angina: final results of the Southampton heart integrated care project (SHIP). The SHIP Collaborative Group. BMJ 1999; 318(7185):706-711.

(131) Botteman MF, Pashos CL, Hauser RS, Laskin BL, Redaelli A. Quality of life aspects of bladder cancer: a review of the literature. Qual Life Res 2003; 12(6):675-688.

(132) Bohle A, Balck F, von WJ, Jocham D. The quality of life during intravesical bacillus Calmette-Guerin therapy. J Urol 1996; 155(4):1221-1226.

(133) Mack D, Frick J. Quality of life in patients undergoing bacille Calmette-Guerin therapy for superficial bladder cancer. Br J Urol 1996; 78(3):369-371.

(134) Schover LR. Sexuality and fertility in urologic cancer patients. Cancer 1987; 60(3 Suppl):553-558.

(135) Vriesema JL, Poucki MH, Kiemeney LA, Witjes JA. Patient opinion of urinary tests versus flexible urethrocystoscopy in follow-up examination for superficial bladder cancer: a utility analysis. Urology 2000; 56(5):793-797.

(136) Aaronson NK, Ahmedzai S, Bergman B, Bullinger M, Cull A, Duez NJ et al. The European Organization for Research and Treatment of Cancer QLQ-C30: a quality-of-life instrument for use in international clinical trials in oncology. J Natl Cancer Inst 1993; 85(5):365-376.

(137) Von Neumann J ea. Theory of games and economic behavior. Princeton: Princeton University Press; 1944.

(138) Fayers PM ea, on behalf of the EORTC Quality of Life Study Group. The EORTC QLQ-C30 Scoring Manual (2nd Edition). Brussels: European Organization for Research and Treatment of Cancer, 1999.

(139) NCI. Priorities of the kidney/bladder cancers progress review group. 2003. Bethesda, US Department of Health and Human Services.
Ref Type: Report

(140) Knowles MA. What we could do now: molecular pathology of bladder cancer. Mol Pathol 2001; 54(4):215-221.

(141) Knowles MA. What we could do now: molecular pathology of bladder cancer. Mol Pathol 2001; 54(4):215-221.

(142) Altman DG, Lyman GH. Methodological challenges in the evaluation of prognostic factors in breast cancer. Breast Cancer Res Treat 1998; 52(1-3):289-303.

(143) Ntzani EE, Ioannidis JP. Predictive ability of DNA microarrays for cancer outcomes and correlates: an empirical assessment. Lancet 2003; 362(9394):1439-1444.

(144) Schmitz-Drager BJ, Goebell PJ, Ebert T, Fradet Y. p53 immunohistochemistry as a prognostic marker in bladder cancer. Playground for urology scientists? Eur Urol 2000; 38(6):691-699.

(145) Knowles MA. What we could do now: molecular pathology of bladder cancer. Mol Pathol 2001; 54(4):215-221.

(146) Chatterjee SJ, Datar R, Youssefzadeh D, George B, Goebell PJ, Stein JP et al. Combined effects of p53, p21, and pRb expression in the progression of bladder transitional cell carcinoma. J Clin Oncol 2004; 22(6):1007-1013.

(147) Billerey C, Chopin D, ubriot-Lorton MH, Ricol D, Gil Diez de MS, Van RB et al. Frequent FGFR3 mutations in papillary non-invasive bladder (pTa) tumors. Am J Pathol 2001; 158(6):1955-1959.

(148) Graf E, Schmoor C, Sauerbrei W, Schumacher M. Assessment and comparison of prognostic classification schemes for survival data. Stat Med 1999; 18(17-18):2529-2545.

(149) Royston P, Sauerbrei W. A new measure of prognostic separation in survival data. Stat Med 2004; 23(5):723-748.

(150) Royston P, Sauerbrei W. Stability of multivariable fractional polynomial models with selection of variables and transformations: a bootstrap investigation. Stat Med 2003; 22(4):639-659.

(151) Altman DG, Royston P. What do we mean by validating a prognostic model? Stat Med 2000; 19(4):453-473.

(152) Royston P, Parmar MK, Sylvester R. Construction and validation of a prognostic model across several studies, with an application in superficial bladder cancer. Stat Med 2004; 23(6):907-926.
