# Supplementary material for: Selenium and Vitamin E for Prevention of Non–Muscle-Invasive Bladder Cancer Recurrence and Progression: A Randomized Clinical Trial
Source: JAMA Netw Open. 2023 Oct 17;6(10):e2337494. doi: 10.1001/jamanetworkopen.2023.37494 (PMC10582794; doi:10.1001/jamanetworkopen.2023.37494)
Supplement: Supplement 2. — eMethods. Additional Methodological Information eResults. Additional Study Results eTable 1. Tumour Pathology of Patients Within the SELENIB Trial eTable 2. Trial Treatment Compliance eTable 3. Trial Treatment Duration eTable 4. Overview of Standard Treatments Received by Patients Within the SELENIB Trial eTable 5. Intravesical BCG Treatment Received by Patients Within the SELENIB Trial eTable 6. Intravesical Chemotherapy Received by Patients Within the SELENIB Trial eTable 7. Adverse Events by CTCAE Grade eTable 8. Adverse Events Occurrence and Incidence During the SELENIB Trial eTable 9. Serious Adverse Events Occurrence and Incidence During the SELENIB Trial eTable 10. Recurrence-Free Survival Estimates at Yearly Intervals for Each Treatment Comparison eTable 11. Recurrence-Free Interval Cox Model Estimates Adjusted for Prognostic Factors eTable 12. Recurrence-Free Interval Cox Model Estimates Adjusted for EAU Risk Group eFigure. Quality-of-Life Analysis eReferences [file jamanetwopen-e2337494-s002.pdf]

Supplementary Online Content

Bryan RT, Pirrie SJ, Abbotts B, et al. Selenium and vitamin E for prevention of non–muscle-invasive bladder cancer recurrence and progression: a randomized clinical trial. *JAMA Netw Open*. 2023;6(10):e2337494. doi:10.1001/jamanetworkopen.2023.37494

eMethods. Additional Methodological Information
eResults. Additional Study Results
eTable 1. Tumour Pathology of Patients Within the SELENIB Trial
eTable 2. Trial Treatment Compliance
eTable 3. Trial Treatment Duration
eTable 4. Overview of Standard Treatments Received by Patients Within the SELENIB Trial
eTable 5. Intravesical BCG Treatment Received by Patients Within the SELENIB Trial
eTable 6. Intravesical Chemotherapy Received by Patients Within the SELENIB Trial
eTable 7. Adverse Events by CTCAE Grade
eTable 8. Adverse Events Occurrence and Incidence During the SELENIB Trial
eTable 9. Serious Adverse Events Occurrence and Incidence During the SELENIB Trial
eTable 10. Recurrence-Free Survival Estimates at Yearly Intervals for Each Treatment Comparison
eTable 11. Recurrence-Free Interval Cox Model Estimates Adjusted for Prognostic Factors
eTable 12. Recurrence-Free Interval Cox Model Estimates Adjusted for EAU Risk Group
eFigure. Quality of Life Analysis
eReferences

This supplementary material has been provided by the authors to give readers additional information about their work.

© 2023 Bryan RT et al. *JAMA Network Open.*

eMethods

ADDITIONAL METHODOLOGICAL INFORMATION

Study Design and Participants

SELENIB recruited patients newly-diagnosed with NMIBC to a double-blinded, placebo-controlled, 2x2 factorial randomised controlled trial between 17th July 2007 and 10th October 2011 in 10 UK hospitals. The trial was registered on ISRCTN 13889738 (24-May-2006) and the EU Clinical Trials Register with EudraCT number 2005-003021-19 (18-Sep-2006). Registration to both was prospective, prior to randomisation of the first patient on 17-Jul-2007. The current version of the protocol (Version 9.0) is available at https://www.birmingham.ac.uk/Documents/college-mds/cancer-genomic-sciences/Bladder-Cancer-Prognosis-Programme-SELENIB-Trial.pdf

Potentially eligible patients were identified at haematuria clinics on the basis of abnormal cystoscopic findings suggestive of UBC and were invited to participate in the trial following TURBT. Eligible patients were adults (aged≥18 years) with newly-diagnosed pathologically-confirmed UBC of UICC stage Ta, T1 or Tis who were able to give informed consent; patients were required to be randomised within 12-months of initial TURBT. To obtain standardized pathological data, 10% of all diagnostic haematoxylin and eosin-stained slides were reviewed centrally. Patients with a previous diagnosis of cancer of the urethra, bladder, ureter or renal pelvis within the last decade, patients who were pregnant or breastfeeding, patients diagnosed with HIV infection, patients on immunosuppressive therapy following organ transplantation, patients taking cyclosporine, and patients with any other condition that might interfere with the safety of the patient or evaluation of the study objectives, were excluded.

Randomisation, Blinding, and Interventions

Patients were randomly assigned equally to one of four groups: oral selenium (200mcg/day high selenium yeast) and matched vitamin E placebo, vitamin E (200IU/day d-alpha-tocopherol) and matched selenium placebo, selenium and vitamin E, or placebo and placebo. Allocation of treatments were blinded to ensure that neither the patient nor the clinical, pharmacy or research personnel were

© 2023 Bryan RT et al. *JAMA Network Open.*

aware of the outcome of randomisation. Randomised treatment allocation was via an in-house computerised algorithm. Randomisation was stratified by recurrence risk group (high versus low/intermediate) and treatment centre. The method of random permuted blocks was used to ensure balance within each stratum defined by recurrence risk group, and a minimisation approach was used to balance treatments within centres. Risk groups were defined as low-risk (solitary G1 pTa tumour, ≤3cm diameter), intermediate risk (multiple G1 pTa tumours, solitary G1 pTa ≥3cm, G2 pTa, G1 pT1, or 1 or 2 G2 pT1 tumours), or high-risk (G3 pTa tumours, G3 pT1, carcinoma in situ, or 3 or more G2 pT1 tumours) [1].

Patients were instructed to take one tablet (selenium or placebo) and one gel capsule (vitamin E or placebo) once daily with food for a period of up to 5 years. Placebos were manufactured to be identical in appearance, smell and taste to the active agents. Their composition except for the active ingredient was identical. Patients were re-supplied with treatments every six months throughout the duration of the treatment period. All study treatments were manufactured, packed and released by *Pharma Nord ApS*, Tinglykke 4-6, 6500 Vojens, Denmark. Patients were otherwise treated according to contemporaneous European Association of Urology (EAU) guidelines for NMIBC [1].

Procedures

Patients attended a SELENIB follow-up clinic every 6-months for up to 5-years following randomisation. At each follow-up, treatment compliance, toxicity and disease status were recorded (patients taking anticoagulant medication had their blood clotting checked at their anticoagulation clinic at 1 week, 2 weeks, and 4 weeks after commencing trial medication). Serious adverse events (SAEs) were defined as any untoward medical occurrence that resulted in death, was life-threatening, required inpatient hospitalisation or prolongation of existing hospitalisation, resulted in persistent or significant disability/incapacity, or that manifest as a congenital anomaly/birth defect.

© 2023 Bryan RT et al. *JAMA Network Open.*

Outcomes

The primary outcome measure was recurrence-free interval, defined as time from date of study entry to date of recurrence. For patients not observed to have experienced recurrence at the time of analysis, the interval was censored at the date last known to be recurrence-free. Recurrence was defined as new occurrence of UBC at the same or different site as the index primary cancer; recurrences at the first 3-month (90-days post TURBT) check cystoscopy were excluded as the majority of such events would predominantly be due to incomplete resection of the index tumour [2], and have little prospect of being affected by chemoprevention. In these cases, patients were censored at date of randomisation. A number of methods for handling these recurrences were investigated in a sensitivity analysis.

Secondary outcome measures included progression-free interval, defined as time from date of randomisation to date of progression. Progression was defined as recurrence with: an increase in grade from grade 1/grade 2 to grade 3, or an increase in T-stage (determined by histopathology), or; the new occurrence of carcinoma in situ (CIS) in a bladder previously free from CIS, or; the new occurrence of multiple urothelial tumours following the initial diagnosis of a solitary urothelial tumour. Progression was also reported if there was the need for a cystectomy because of refractory disease, or the new development of nodal and/or distant metastases (determined by imaging). For those patients not observed to have experienced progression by the time of analysis, the interval was censored at the date last known to be progression-free.

Other secondary outcome measures included: overall survival time, defined as time from date of randomisation to the date of death from any cause. Patients alive at the time of analysis were censored at the date last known to be alive; incidence of other clinically-diagnosed malignancies, incidence of cardiovascular events and incidence of diabetes: calculated by dividing the total number of all other clinically-diagnosed malignancies by the total number of years at risk (years at risk in each arm if comparing between arms). Other clinically-diagnosed malignancies may be pathologically confirmed or diagnosed based on strong clinical, radiological, or cytological evidence, or on a

laboratory marker; death from cardiovascular causes, the number of deaths in each arm from cardiovascular causes was reported; and quality of life, assessed at each follow-up visit by EORTC QLQ-C30 [3], QLQ-BLS24 and QLQ-BLM30 [4,5] (questionnaire-responses were combined and transformed into dimension-scores [6]).

Statistical Analysis

The statistical analysis addressed the primary hypothesis on an intention-to-treat basis. As a 2x2 factorial design, the analysis consisted of two comparisons: firstly, all patients randomised to selenium with all those randomised to the associated placebo, stratifying by vitamin E allocation; secondly, all patients randomised to vitamin E with all those randomised to the associated placebo, stratifying by selenium allocation. An interaction was not expected. Kaplan-Meier estimates of recurrence-free and progression-free interval were used to compare treatment groups descriptively, whilst log-rank tests were used to test the hypothesis of no difference between treatments. Hazard ratios comparing treatments were estimated from Cox proportional hazards regression models, both unadjusted and adjusted for known prognostic factors. The same methods were used for the secondary survival outcomes.

Sample size for the 2x2 factorial design was based on two *a priori* independent hypotheses being tested, one relating to selenium and one to vitamin E. We estimated that the 5-year recurrence rate would be 45% (55% recurrence-free) following contemporary standard-of-care treatment [1]. To detect an absolute increase of 12% in the recurrence-free rate by either of the agents vs. their associated placebo (i.e., 55% to 67%) using a log-rank test, 460 patients were needed in total to give 80% power with two-sided significance level of 5%. This calculation was based on an accrual period of 6 years and a minimum follow-up period of 3 years. Allowing for an additional 12% for loss to follow-up resulted in a total requirement of 515 patients.

An independent Data Monitoring Committee (iDMC) reviewed trial progress annually and made recommendations to the BCPP working group. A Trial Steering Committee (TSC) was also established

© 2023 Bryan RT et al. *JAMA Network Open.*

to provide oversight of the trial, focusing on trial progress, protocol adherence, patient safety and emerging evidence. Trial stopping criteria was considered by both the iDMC and TSC and comprised: unacceptable recruitment rates or data quality; excessive toxicity; clinically convincing differences in treatment at interim analysis; new information regarding trial therapies making the continuation of the trial unethical.

Following early termination of the trial, Hospital Episode Statistics (HES) extracts (NHS Data Access Advisory Group application NIC-157371-GG78N, reference 031012-a) were used to identify potential recurrence and progression events until 26th September 2018 in those patients already recruited; potential events identified in this way prompted site queries and confirmation.

Role of the Funding Source

The funder of the study had no role in trial design, data collection, analysis or interpretation or writing of the report. The corresponding author had full access to all the data in the trial and had final responsibility for the decision to submit for publication.

© 2023 Bryan RT et al. *JAMA Network Open.*

eResults

ADDITIONAL STUDY RESULTS

Trial Treatment

The duration of treatment for each of the four arms is shown in eTable 2 and eTable 3. Compliance to treatment was recorded in three ways: (i) patients returned packaging with any remaining tablets; (ii) patients completed a diary recording any missed tablets which they missed; (iii) patients were asked to recall whether they had missed any tablets. Across these three approaches, the median percentage of days of taking trial treatment was >95% (eTable 2 and eTable 3). Out of 270 participants, 264 (98%) had at least 3 years follow-up or died within 3 years of randomisation. Only 2 patients were censored within 1 year of randomisation.

Standard Treatments

Standard-of-care treatments received by participants within the trial are shown in eTables 4, 5, and 6.

Adverse Events

eTables 7, 8, and 9 show adverse events by CTCAE grade.

There were 37 cardiac events involving 29 patients which impacted similar proportions of patients in both comparisons (selenium active 10% vs placebo 11%, vitamin E active 11% vs placebo 11%). There were 7 patients who experienced an adverse event, serious adverse event, or whose cause of death was partly due to diabetes (selenium active 1% vs placebo 4%, vitamin E active 4% vs placebo 1%). There were 21 new neurological events (e.g. TIA or stroke) or exacerbations of existing neurological conditions (e.g. Parkinson's Disease) involving 18 patients which impacted similar proportions of patients in both comparisons (selenium active 4% vs placebo 9%, vitamin E active 7% vs placebo 6%). There were 22 new malignancies involving 21 patients which impacted similar proportions of patients in both comparisons (selenium active 7% vs placebo 8%, vitamin E active 6% vs placebo 9%).

© 2023 Bryan RT et al. *JAMA Network Open.*

Primary Outcome

Interaction between selenium and vitamin E was tested using a Cox proportional hazards regression model including an interaction term. Interaction was found to be non-significant (p=0.85), thus confirming the suitability of the factorial design.

Sensitivity analysis was performed to assess robustness of the primary analysis. The following scenarios were investigated: excluding patients who recur within 90 days of TURBT; including all recurrences regardless of timing; censoring at randomisation those patients who recurred prior to randomisation or who recurred within 90 days of TURBT; including bladder cancer deaths as an event; using date recurrence detected rather than date recurrence confirmed as primary recurrence date; ignoring first recurrence if within 90 days of TURBT and using either second recurrence as event or censoring at date last seen; calculating RFI from date of TURBT instead of date of randomisation. The majority of the sensitivity analysis was concerned with the patients who recurred within 90 days of TURBT and were censored at randomisation date in the primary analysis. As there were only four such patients the different methods of accounting for these patients did not have a large effect on the results overall. Including deaths due to bladder cancer did not affect the analyses as all patients who died due to bladder cancer had already recurred. Calculating RFI from date of TURBT increased RFI by 3 months on average.

Adjusted analyses of the primary outcome were also performed (eTables 11 & 12). A single Cox proportional hazards model was constructed including both treatments and adjusted for known prognostic factors: tumour grade (1 vs 2 vs 3), stage (pTa vs pT1) and carcinoma in situ (absent vs present). Time varying effects were used to account for non-proportionality within stage. In this analysis the effects of both selenium and vitamin E remained largely unchanged: selenium HR 0.90 (95%CI 0.63, 1.29: p=0.55) and vitamin E HR 1.49 (95% CI 1.04, 2.15: p=0.031). A further model was fitted including both treatments and stratifying for the baseline EAU risk group (low, intermediate, high, very high).[7] After adjusting for risk group, the effect of both selenium and vitamin E remain

© 2023 Bryan RT et al. *JAMA Network Open.*

largely unchanged: selenium HR 0.92 (95% CI: 0.64, 1.31: p=0.63) and vitamin E HR 1.42 (95% CI: 0.99, 2.05: p=0.058).

At baseline 250 patients completed the quality of life questionnaire. In total, 233 questionnaires from 135 patients were returned during follow up. Of these, 17 questionnaires could not be included in the following analysis as the date of interview/completion was missing. Questionnaires were grouped into 3 monthly intervals (give or take 1 month) resulting in 126 follow up questionnaires plus 250 baseline questionnaires included in the descriptive analysis. No differences between any of the arms were observed (eFigure).

© 2023 Bryan RT et al. *JAMA Network Open.*

eTable 1: Tumour pathology of patients within the SELENIB trial

Comparison Group:	Selenium		Vitamin E		
	**Placebo (N = 136)**	**Active (N = 134)**	**Placebo (N = 130)**	**Active (N = 140)**	**Overall (N = 270)**
**Histology**					
Transitional cell carcinoma (TCC)	130 (96%)	126 (94%)	122 (94%)	134 (96%)	256 (95%)
TCC & adenocarcinomatous elements	2 (1.5%)	0 (0%)	2 (1.5%)	0 (0%)	2 (0.7%)
TCC & adenocarcinomatous elements & squamous elements	1 (0.7%)	0 (0%)	1 (0.8%)	0 (0%)	1 (0.4%)
TCC & sarcomatous elements	0 (0%)	1 (0.7%)	0 (0%)	1 (0.7%)	1 (0.4%)
TCC & squamous elements	1 (0.7%)	3 (2.2%)	2 (1.5%)	2 (1.4%)	4 (1.5%)
TCC & other	1 (0.7%)	2 (1.5%)	2 (1.5%)	1 (0.7%)	3 (1.1%)
Other	0 (0%)	1 (0.7%)	0 (0%)	1 (0.7%)	1 (0.4%)
Unknown	1 (0.7%)	1 (0.7%)	1 (0.8%)	1 (0.7%)	2 (0.7%)
**Dominant subtype**					
TCC	125 (92%)	126 (94%)	123 (95%)	128 (91%)	251 (93%)
Squamous	1 (0.7%)	0 (0%)	1 (0.8%)	0 (0%)	1 (0.4%)
Other	1 (0.7%)	1 (0.7%)	1 (0.8%)	1 (0.7%)	2 (0.7%)
Unknown	9 (6.6%)	7 (5.2%)	5 (3.8%)	11 (7.9%)	16 (5.9%)
**Growth pattern**					
Papillary	114 (84%)	112 (84%)	109 (84%)	117 (84%)	226 (84%)
Solid	5 (3.7%)	3 (2.2%)	3 (2.3%)	5 (3.6%)	8 (3.0%)
Mixed	13 (9.6%)	16 (12%)	16 (12%)	13 (9.3%)	29 (11%)
In-situ	2 (1.5%)	3 (2.2%)	2 (1.5%)	3 (2.1%)	5 (1.9%)
Unknown	2 (1.5%)	0 (0%)	0 (0%)	2 (1.4%)	2 (0.7%)
**Detrusor muscle**					
Yes	94 (69%)	97 (72%)	96 (74%)	95 (68%)	191 (71%)
No	39 (29%)	35 (26%)	33 (25%)	41 (29%)	74 (27%)

© 2023 Bryan RT et al. *JAMA Network Open.*

Comparison Group:	Selenium		Vitamin E		
	Placebo (N = 136)	Active (N = 134)	Placebo (N = 130)	Active (N = 140)	Overall (N = 270)
Unknown	3 (2.2%)	2 (1.5%)	1 (0.8%)	4 (2.9%)	5 (1.9%)

Note: Data are n (%)

© 2023 Bryan RT et al. *JAMA Network Open.*

eTable 2: Trial treatment compliance

Comparison Group:	Selenium		Vitamin E		
	Placebo	Active	Placebo	Active	Overall
**Compliance method: Returned tablets**					
**Selenium/placebo tablets taken**					
Mean (SD)	637 (403)	556 (400)	610 (407)	586 (400)	597 (403)
Median (IQR)	539 (318, 943)	512 (233, 841)	520 (291, 877)	517 (290, 873)	520 (290, 876)
Range	5, 1,529	0, 1,531	0, 1,529	5, 1,531	0, 1,531
**Percentage of days selenium/placebo taken**					
Mean (SD)	92 (13)	89 (18)	91 (15)	90 (16)	91 (16)
Median (IQR)	97 (91, 99)	95 (86, 99)	97 (90, 99)	96 (89, 99)	96 (90, 99)
Range	3, 100	0, 100	0, 100	3, 100	0, 100
**Vitamin E/placebo tablets taken**					
Mean (SD)	639 (403)	557 (402)	612 (409)	587 (400)	599 (404)
Median (IQR)	539 (318, 943)	512 (233, 852)	520 (291, 877)	517 (290, 873)	520 (290, 876)
Range	5, 1,529	0, 1,531	0, 1,529	5, 1,531	0, 1,531
**Percentage of days vitamin E/placebo taken**					
Mean (SD)	92 (13)	89 (17)	91 (15)	90 (15)	91 (15)
Median (IQR)	97 (91, 99)	95 (88, 99)	96 (90, 99)	96 (89, 99)	96 (90, 99)
Range	3, 100	0, 100	0, 100	3, 100	0, 100
**Compliance method: Diary**					
**Selenium/placebo tablets taken**					
Mean (SD)	652 (408)	577 (399)	631 (409)	602 (401)	616 (404)
Median (IQR)	540 (339, 1,034)	514 (250, 870)	522 (320, 888)	524 (306, 876)	522 (306, 879)
Range	31, 1,556	8, 1,545	30, 1,556	8, 1,545	8, 1,556
**Percentage of days selenium/placebo taken**					
Mean (SD)	95 (10)	95 (9)	96 (8)	94 (11)	95 (10)

© 2023 Bryan RT et al. *JAMA Network Open.*

Comparison Group:	Selenium		Vitamin E		
	Placebo	Active	Placebo	Active	Overall
Median (IQR)	99 (95, 100)	98 (95, 100)	98 (95, 100)	99 (94, 100)	99 (95, 100)
Range	27, 100	31, 100	37, 100	27, 100	27, 100
**Vitamin E/placebo tablets taken**					
Mean (SD)	650 (410)	575 (398)	629 (410)	599 (401)	613 (405)
Median (IQR)	540 (337, 1,025)	514 (275, 866)	520 (320, 882)	524 (307, 870)	521 (310, 876)
Range	31, 1,556	8, 1,545	30, 1,556	8, 1,545	8, 1,556
**Percentage of days vitamin E/placebo taken**					
Mean (SD)	94 (12)	95 (10)	96 (8)	94 (13)	95 (11)
Median (IQR)	98 (95, 100)	98 (95, 100)	98 (95, 100)	99 (94, 100)	98 (95, 100)
Range	20, 100	31, 100	37, 100	20, 100	20, 100
**Compliance method: Patient recollection**					
**Selenium/placebo tablets taken**					
Mean (SD)	664 (411)	589 (407)	640 (418)	615 (404)	627 (410)
Median (IQR)	540 (342, 1,011)	520 (233, 873)	522 (342, 891)	533 (315, 880)	526 (339, 884)
Range	83, 1,556	8, 1,569	30, 1,569	8, 1,545	8, 1,569
**Percentage of days selenium/placebo taken**					
Mean (SD)	98 (4)	97 (7)	98 (5)	97 (7)	97 (6)
Median (IQR)	100 (98, 100)	100 (98, 100)	100 (98, 100)	100 (97, 100)	100 (98, 100)
Range	72, 100	49, 100	65, 100	49, 100	49, 100
**Vitamin E/placebo tablets taken**					
Mean (SD)	664 (411)	589 (406)	640 (417)	615 (404)	627 (410)
Median (IQR)	540 (342, 1,011)	520 (233, 873)	522 (342, 891)	533 (315, 880)	526 (339, 884)
Range	83, 1,556	8, 1,569	30, 1,569	8, 1,545	8, 1,569
**Percentage of days vitamin E/placebo taken**					
Mean (SD)	98 (4)	97 (7)	98 (5)	97 (7)	97 (6)
Median (IQR)	100 (98, 100)	100 (98, 100)	100 (98, 100)	100 (97, 100)	100 (98, 100)
Range	72, 100	49, 100	65, 100	49, 100	49, 100

© 2023 Bryan RT et al. *JAMA Network Open.*

eTable 3: Trial Treatment Duration

Comparison Group	Selenium		Vitamin E		
	Placebo	Active	Placebo	Active	Overall
**Patients with Follow-up**	121 (89%)	117 (87%)	113 (87%)	125 (89%)	238 (88%)
**Duration of treatment (years)**					
Mean (SD)	1·9 (1·2)	1·7 (1·1)	1·8 (1·2)	1·7 (1·1)	1·8 (1·1)
Median (IQR)	1·7 (0·9, 2·9)	1·4 (0·8, 2·4)	1·4 (0·9, 2·5)	1·5 (0·9, 2·5)	1·5 (0·9, 2·5)
Range	0·2, 4·3	0·0, 4·3	0·1, 4·3	0·0, 4·2	0·0, 4·3
**Recurrences**	60 (49%)	62 (51%)	50(41%)	72 (59%)	122 (100%)
**Progressions**	17 (46%)	20 (54%)	17 (46%)	20 (54%)	37 (100%)
**Deaths**	29 (55%)	24 (45%)	27 (51%)	26 (49%)	53 (100%)

SD, standard deviation; IQR, interquartile range.

© 2023 Bryan RT et al. *JAMA Network Open.*

eTable 4: Overview of Standard Treatments Received by Patients Within the SELENIB Trial

Comparison Group	Selenium		Vitamin E		
	Placebo (N = 136)	Active (N = 134)	Placebo (N = 130)	Active (N = 140)	Overall (N = 270)
BCG	30 (22%)	26 (19%)	24 (18%)	32 (23%)	56 (21%)
Intravesical chemotherapy (course)	3 (2·2%)	4 (3·0%)	3 (2·3%)	4 (2·9%)	7 (2·6%)
Intravesical chemotherapy (single instillation)	42 (31%)	36 (27%)	42 (32%)	36 (26%)	78 (29%)
Intravesical chemotherapy (single instillation) & BCG	12 (8·8%)	16 (12%)	15 (12%)	13 (9·3%)	28 (10%)
Intravesical chemotherapy (single instillation) & Intravesical chemotherapy (course)	0 (0%)	2 (1·5%)	2 (1·5%)	0 (0%)	2 (0·7%)
No treatment recorded	49 (36%)	50 (37%)	44 (34%)	55 (39%)	99 (37%)

Note: Data are n (%). Treatments are only included in they began prior to recurrence/progression.

BCG, Bacillus Calmette–Guérin vaccine.

© 2023 Bryan RT et al. *JAMA Network Open.*

eTable 5: Intravesical BCG treatment received by patients within the SELENIB trial

Comparison Group:	Selenium		Vitamin E		
	Placebo (N = 136)	Active (N = 134)	Placebo (N = 130)	Active (N = 140)	Overall (N = 270)
**Received BCG**					
Yes	42 (31%)	42 (31%)	39 (30%)	45 (32%)	84 (31%)
No	91 (67%)	85 (63%)	84 (65%)	92 (66%)	176 (65%)
Unknown	3 (2.2%)	7 (5.2%)	7 (5.4%)	3 (2.1%)	10 (3.7%)
**Number of schedules**					
Mean (SD)	3.31 (2.49)	3.12 (1.95)	3.08 (2.08)	3.33 (2.36)	3.21 (2.23)
Median (IQR)	2.00 (2.00, 5.50)	2.00 (2.00, 4.00)	2.00 (2.00, 4.00)	2.00 (2.00, 4.00)	2.00 (2.00, 4.00)
Range	1.00, 8.00	1.00, 8.00	1.00, 8.00	1.00, 8.00	1.00, 8.00
**Schedules completed**					
Mean (SD)	2.52 (2.72)	2.40 (2.23)	2.31 (2.39)	2.60 (2.56)	2.46 (2.47)
Median (IQR)	1.00 (1.00, 3.75)	1.00 (1.00, 3.75)	1.00 (1.00, 3.50)	1.00 (1.00, 4.00)	1.00 (1.00, 4.00)
Range	0.00, 8.00	0.00, 8.00	0.00, 8.00	0.00, 8.00	0.00, 8.00
**Total number of instillations**					
Mean (SD)	16 (8)	15 (6)	16 (7)	15 (7)	16 (7)
Median (IQR)	15 (9, 24)	15 (11, 20)	15 (9, 21)	15 (9, 21)	15 (9, 21)
Range	2, 27	6, 29	6, 27	2, 29	2, 29
**Total duration (weeks)**					
Mean (SD)	15.4 (16.4)	10.3 (5.0)	12.9 (13.0)	12.5 (11.3)	12.7 (12.0)
Median (IQR)	10.4 (6.3, 17.0)	9.3 (6.2, 13.7)	7.1 (6.3, 14.0)	9.9 (6.3, 15.2)	9.9 (6.3, 15.0)
Range	4.7, 69.9	2.0, 24.0	5.0, 69.9	2.0, 64.0	2.0, 69.9

© 2023 Bryan RT et al. *JAMA Network Open.*

eTable 6: Intravesical chemotherapy received by patients within the SELENIB trial

Comparison Group:	Selenium		Vitamin E		
	Placebo (N = 136)	Active (N = 134)	Placebo (N = 130)	Active (N = 140)	Overall (N = 270)
**Single instillation received**					
Yes	54 (40%)	54 (40%)	59 (45%)	49 (35%)	108 (40%)
No	79 (58%)	73 (54%)	64 (49%)	88 (63%)	152 (56%)
Unknown	3 (2.2%)	7 (5.2%)	7 (5.4%)	3 (2.1%)	10 (3.7%)
**Single instillation agent**					
Epirubicin	10 (19%)	10 (19%)	10 (17%)	10 (20%)	20 (19%)
Mitomycin C	36 (67%)	27 (50%)	33 (56%)	30 (61%)	63 (58%)
Other	0 (0%)	1 (1.9%)	1 (1.7%)	0 (0%)	1 (0.9%)
Unknown	8 (15%)	16 (30%)	15 (25%)	9 (18%)	24 (22%)
**Course received**					
Yes	3 (2.2%)	6 (4.5%)	5 (3.8%)	4 (2.9%)	9 (3.3%)
No	130 (96%)	121 (90%)	118 (91%)	133 (95%)	251 (93%)
Unknown	3 (2.2%)	7 (5.2%)	7 (5.4%)	3 (2.1%)	10 (3.7%)
**Course agent**					
Mitomycin C	2 (67%)	6 (100%)	5 (100%)	3 (75%)	8 (89%)
Unknown	1 (33%)	0 (0%)	0 (0%)	1 (25%)	1 (11%)
**Course instillations given**					
4	1 (33%)	0 (0%)	1 (20%)	0 (0%)	1 (11%)
6	2 (67%)	6 (100%)	4 (80%)	4 (100%)	8 (89%)
**Course completed**					
Yes	3 (100%)	6 (100%)	5 (100%)	4 (100%)	9 (100%)

© 2023 Bryan RT et al. *JAMA Network Open.*

eTable 7: Adverse events by CTCAE grade

Comparison Group	Selenium		Vitamin E		
	**Placebo**	**Active**	**Placebo**	**Active**	**Overall**
**Grade**					
1	935 (90%)	830 (90%)	839 (91%)	926 (89%)	1,765 (90%)
2	32 (3·1%)	32 (3·5%)	23 (2·5%)	41 (4·0%)	64 (3·3%)
3	16 (1·5%)	8 (0·9%)	10 (1·1%)	14 (1·4%)	24 (1·2%)
4	0 (0%)	3 (0·3%)	3 (0·3%)	0 (0%)	3 (0·2%)
Unknown	53 (5·1%)	48 (5·2%)	46 (5·0%)	55 (5·3%)	101 (5·2%)
Total	1,036 (100%)	921 (100%)	921 (100%)	1,036 (100%)	1,957 (100%)

Note: Data are n (%)

© 2023 Bryan RT et al. *JAMA Network Open.*

eTable 8: Adverse events occurrence and incidence during the SELENIB trial

Comparison Group:	Selenium		Vitamin E		
**Adverse Event**	**Placebo**	**Active**	**Placebo**	**Active**	**Overall**
Abdominal pain	45 (30)	33 (26)	34 (27)	44 (29)	78 (56)
Anaemia	0 (0)	1 (1)	1 (1)	0 (0)	1 (1)
Blurred vision	41 (17)	31 (21)	41 (22)	31 (16)	72 (38)
Boil	1 (1)	0 (0)	1 (1)	0 (0)	1 (1)
Bronchitis	13 (8)	19 (14)	16 (11)	16 (11)	32 (22)
Cardiac	13 (8)	10 (8)	11 (7)	12 (9)	23 (16)
Cateract	1 (1)	0 (0)	0 (0)	1 (1)	1 (1)
CNS	1 (1)	0 (0)	1 (1)	0 (0)	1 (1)
Constipation	6 (5)	1 (1)	3 (2)	4 (4)	7 (6)
Cough and/or cold	126 (59)	91 (45)	101 (46)	116 (58)	217 (104)
Depression/anxiety	6 (5)	5 (5)	6 (6)	5 (4)	11 (10)
Dermatitis	93 (48)	97 (47)	95 (46)	95 (49)	190 (95)
Diabetes	4 (4)	2 (1)	1 (1)	5 (4)	6 (5)
Diarrhoea	72 (42)	57 (35)	74 (38)	55 (39)	129 (77)
Dizziness	65 (37)	71 (35)	49 (31)	87 (41)	136 (72)
Dysuria	1 (1)	0 (0)	0 (0)	1 (1)	1 (1)
Fall	5 (5)	6 (5)	4 (3)	7 (7)	11 (10)
Fatigue	146 (61)	133 (57)	138 (57)	141 (61)	279 (118)
Flatulence	1 (1)	0 (0)	1 (1)	0 (0)	1 (1)
Glaucoma	0 (0)	1 (1)	0 (0)	1 (1)	1 (1)
Haematuria	0 (0)	1 (1)	0 (0)	1 (1)	1 (1)
Hair loss/brittle hair	27 (17)	33 (18)	27 (21)	33 (14)	60 (35)
Hernia	2 (2)	0 (0)	1 (1)	1 (1)	2 (2)
Infection	20 (15)	12 (9)	15 (11)	17 (13)	32 (24)
Insomnia	1 (1)	0 (0)	0 (0)	1 (1)	1 (1)
Irritability	43 (27)	40 (26)	36 (23)	47 (30)	83 (53)
Mass	0 (0)	3 (3)	2 (2)	1 (1)	3 (3)
Memory loss	3 (2)	0 (0)	3 (2)	0 (0)	3 (2)
Nail tenderness	26 (19)	23 (19)	22 (18)	27 (20)	49 (38)
Nausea	61 (43)	53 (32)	50 (33)	64 (42)	114 (75)
Other	65 (38)	61 (42)	53 (38)	73 (42)	126 (80)
Pain	16 (13)	14 (12)	10 (9)	20 (16)	30 (25)
Rash	1 (1)	0 (0)	0 (0)	1 (1)	1 (1)
Shortness of breath	3 (2)	4 (4)	6 (5)	1 (1)	7 (6)
Upset stomach	79 (44)	65 (40)	72 (41)	72 (43)	144 (84)
Weakness	47 (28)	53 (32)	44 (28)	56 (32)	100 (60)
Weight change	2 (1)	1 (1)	3 (2)	0 (0)	3 (2)
**Overall**	**1036 (107)**	**921 (103)**	**921 (94)**	**1036 (116)**	**1957 (210)**

Note: Data are # occurrences (# patients affected)

© 2023 Bryan RT et al. *JAMA Network Open.*

eTable 9: Serious adverse events occurrence and incidence during the SELENIB trial

Comparison Group:	Selenium		Vitamin E		
**Category**	**Placebo**	**Active**	**Placebo**	**Active**	**Overall**
Allergy/Immunology	1 (1)	0 (0)	1 (1)	0 (0)	1 (1)
Cardiac Arrhythmia	2 (2)	2 (2)	1 (1)	3 (3)	4 (4)
Cardiac General	3 (3)	7 (6)	4 (4)	6 (5)	10 (9)
Death	1 (1)	3 (3)	4 (4)	0 (0)	4 (4)
Endocrine	1 (1)	0 (0)	0 (0)	1 (1)	1 (1)
Gastrointestinal	0 (0)	1 (1)	0 (0)	1 (1)	1 (1)
Hemorrhage/Bleeding	1 (1)	0 (0)	0 (0)	1 (1)	1 (1)
Hepatobiliary/Pancreas	1 (1)	0 (0)	1 (1)	0 (0)	1 (1)
Infection	10 (4)	3 (3)	9 (3)	4 (4)	13 (7)
Lymphatics	0 (0)	1 (1)	0 (0)	1 (1)	1 (1)
Metabolic/Laboratory	1 (1)	0 (0)	0 (0)	1 (1)	1 (1)
Musculoskeletal/Soft Tissue	8 (5)	1 (1)	3 (3)	6 (3)	9 (6)
Neurology	4 (3)	3 (2)	5 (4)	2 (1)	7 (5)
Ocular/Visual	4 (3)	0 (0)	0 (0)	4 (3)	4 (3)
Pain	2 (2)	2 (2)	4 (4)	0 (0)	4 (4)
Pulmonary/Upper Respiratory	5 (5)	1 (1)	3 (3)	3 (3)	6 (6)
Renal/Genitourinary	5 (4)	3 (3)	4 (4)	4 (3)	8 (7)
Secondary Malignancy	1 (1)	1 (1)	2 (2)	0 (0)	2 (2)
Surgery/Intra-operative Injury	1 (1)	2 (2)	0 (0)	3 (3)	3 (3)
Trauma	0 (0)	1 (1)	0 (0)	1 (1)	1 (1)
Vascular	2 (2)	1 (1)	2 (2)	1 (1)	3 (3)
**Overall**	**53 (32)**	**32 (24)**	**43 (29)**	**42 (27)**	**85 (56)**

Note: Data are # occurrences (# patients affected)

© 2023 Bryan RT et al. *JAMA Network Open.*

eTable 10: Recurrence-Free Survival Estimates at Yearly Intervals for Each Treatment Comparison

	Year 1	Year 2	Year 3	Year 4	Year 5
**Vitamin E Comparison**					
Placebo	80 (74, 87)	72 (65, 80)	63 (54, 72)	62 (54, 71)	60 (51, 69)
Vitamin E	72 (65, 80)	58 (51, 67)	51 (44, 61)	36 (39, 56)	46(39, 56)
**Selenium Comparison**					
Placebo	72 (64, 87)	61 (53, 70)	57 (49, 66)	54 (46, 63)	52 (44, 62)
Selenium	80 (74, 87)	69 (61, 77)	57 (49, 66)	53 (45, 63)	53 (45, 63)

Note: Data are survival probability estimates (95% confidence interval).

© 2023 Bryan RT et al. *JAMA Network Open.*

eTable 11: Recurrence-free interval Cox model estimates adjusted for prognostic factors

	Hazard Ratio	95% Confidence Interval	p-value
Selenium allocation			
Placebo (reference)	—	—	
Active	0.90	0.63, 1.29	0.55
Vitamin E allocation			
Placebo (reference)	—	—	
Active	1.49	1.04, 2.15	0.031
Grade			
Grade 1 (reference)	—	—	
Grade 2	0.88	0.56, 1.40	0.59
Grade 3	0.56	0.31, 1.03	0.064
Stage			
pTa (reference)	—	—	
pT1	0.20	0.00, 342	0.67
CIS			
Absent (reference)	—	—	
Present	1.29	0.70, 2.39	0.42
**Time varying effects**			
Stage	1.47	0.28, 7.83	0.65

CIS, carcinoma *in situ*

eTable 12: Recurrence-free interval Cox model estimates adjusted for EAU risk group

	Hazard Ratio	95% Confidence Interval	p-value
Selenium allocation			
Placebo (reference)	—	—	
Active	0.92	0.64, 1.31	0.63
Vitamin E allocation			
Placebo (reference)	—	—	
Active	1.42	0.99, 2.05	0.058

A single Cox proportional hazards model was constructed including both treatments and adjusted for known prognostic factors: tumour grade (1 vs 2 vs 3), stage (pTa vs pT1), and carcinoma *in situ* (absent vs present); time varying effects were used to account for non-proportionality within stage (Table S5A). A further model was fitted including both treatments and stratifying for the baseline European Association of Urology (EAU) risk group (low, intermediate, high, very high) [8].

© 2023 Bryan RT et al. *JAMA Network Open.*

eFigure: Quality of Life Analysis

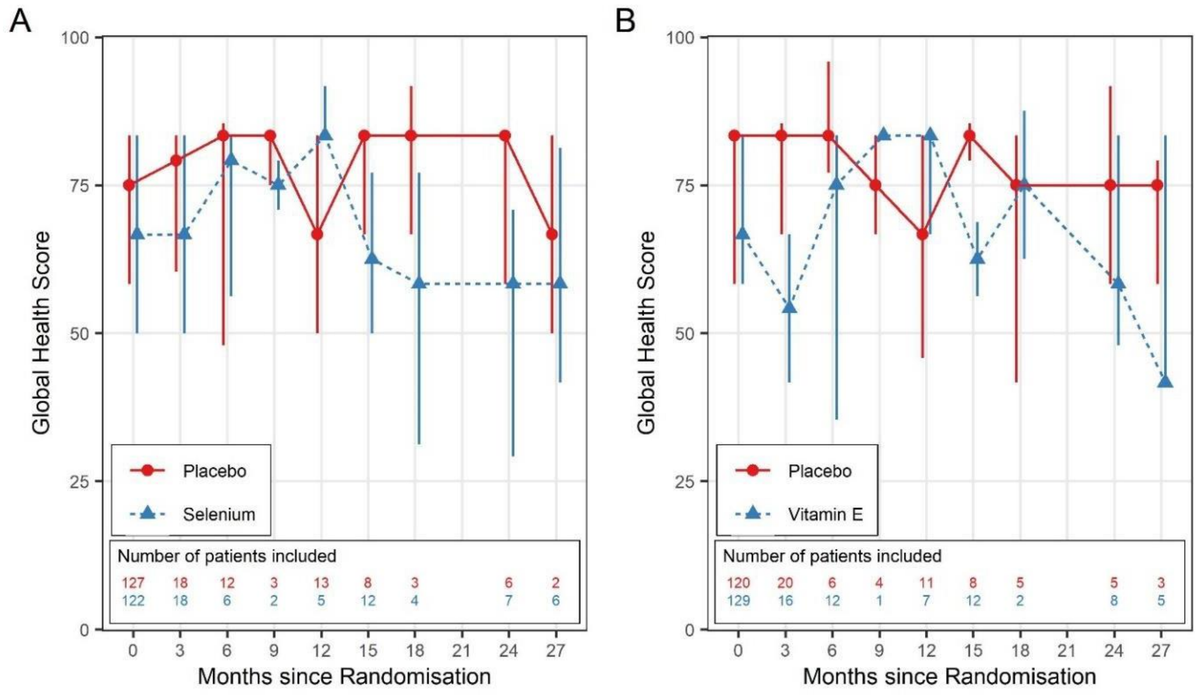

Assessed at each follow-up visit, EORTC QLQ-C30, QLQ-BLS24, and QLQ-BLM30 [5,6] were used to assess quality of life of patients receiving selenium (A) or vitamin E (B). Questionnaires were grouped into three monthly intervals (± one month) resulting in 126 follow up questionnaires plus 250 baseline questionnaires included in the analysis. Of these, 17 questionnaires could not be included in the following analysis as the date of interview/completion was missing.

Median scores are plotted with inter-quartile range error bars.

© 2023 Bryan RT et al. *JAMA Network Open.*

eReferences

1. Oosterlinck W, Lobel B, Jakse G, Malmstrom PU, Stockle M, Sternberg C. Guidelines on bladder cancer. *Eur Urol.* 2002;41(2):105-112.
2. Bryan RT, Collins SI, Daykin MC, et al. Mechanisms of recurrence of Ta/T1 bladder cancer. *Ann R Coll Surg Engl.* 2010;92(6):519-524.
3. Aaronson NK, Ahmedzai S, Bergman B, et al. The European Organization for Research and Treatment of Cancer QLQ-C30: A Quality-of-Life Instrument for Use in International Clinical Trials in Oncology. *JNCI: Journal of the National Cancer Institute.* 1993;85(5):365-376.
4. Blazeby JM, Hall E, Aaronson NK, et al. Validation and reliability testing of the EORTC QLQ-NMIBC24 questionnaire module to assess patient-reported outcomes in non-muscle-invasive bladder cancer. *Eur Urol.* 2014;66(6):1148-1156.
5. Danna BJ, Metcalfe MJ, Wood EL, Shah JB. Assessing Symptom Burden in Bladder Cancer: An Overview of Bladder Cancer Specific Health-Related Quality of Life Instruments. *Bladder Cancer.* 2016;2(3):329-340.
6. Fayers P, Aaronson NK, Bjordal K, Groenvold M, Curran D, Bottomley A. *EORTC QLQ-C30 Scoring Manual.* 3rd ed. Brussels2001.
7. Babjuk M, Burger M, Capoun O, et al. European Association of Urology Guidelines on Non-muscle-invasive Bladder Cancer (Ta, T1, and Carcinoma in Situ). *Eur Urol.* 2022;81(1):75-94.
8. Babjuk M, Burger M, Capoun O, et al. European Association of Urology Guidelines on Non-muscle-invasive Bladder Cancer (Ta, T1, and Carcinoma in Situ). *Eur Urol.* 2021.

© 2023 Bryan RT et al. *JAMA Network Open.*
