# Supplementary material for: Selenium and Vitamin E for Prevention of Non–Muscle-Invasive Bladder Cancer Recurrence and Progression: A Randomized Clinical Trial
Source: JAMA Netw Open. 2023 Oct 17;6(10):e2337494. doi: 10.1001/jamanetworkopen.2023.37494 (PMC10582794; doi:10.1001/jamanetworkopen.2023.37494)
Supplement: Supplement 3. — Nonauthor Collaborators [file jamanetwopen-e2337494-s003.pdf]

Supplemental Online Content: Nonauthor Collaborators
*First name, last name, and suffix (if applicable) are required and will appear in PubMed.

*Group Name(s): SELENIB Investigators

*First Name and Middle Initial(s)	*Last Name	*Suffix (eg, Jr, III)	Academic Degrees	Institution	Location (city, state/province, country)	Role or Contribution, eg, chair, principal investigator	Group (if more than 1 Group listed in the byline) and/or Subgroup (eg, Steering Committee)
Gun	Choudry		FRCS(Urol)	Queen's Hospital	Burton Upon Trent, West Midlands, UK	Investigator	
Sikander	Khwaja		FRCS(Urol)	Queen's Hospital	Burton Upon Trent, West Midlands, UK	Investigator	
Ken	Desai		FRCS(Urol)	University Hospital Coventry & Warwickshire	Coventry, West Midlands, UK	Investigator	
Kieran P	Jefferson		FRCS(Urol)	University Hospital Coventry & Warwickshire	Coventry, West Midlands, UK	Investigator	
Lawrence A	Emtage		FRCS(Urol)	Russell’s Hall Hospital	Dudley, West Midlands, UK	Investigator	
Aniruddha	Chakravarti		FRCS(Urol)	Russell’s Hall Hospital	Dudley, West Midlands, UK	Investigator	
Michael C	Foster		FRCS(Urol)	Good Hope Hospital	Sutton Coldfield, West Midlands, UK	Investigator	
B Dev	Sarmar		FRCS(Urol)	Birmingham Heartlands Hospital	Birmingham, West Midlands, UK	Investigator	
Christopher J	Luscombe		FRCS(Urol)	Royal Stoke University Hospital	Stoke-on-Trent, West Midlands, UK	Investigator	
D Michael A	Wallace		FRCS(Urol)	Queen Elizabeth Hospital	Birmingham, West Midlands, UK	Investigator	
Nicholas J	James		FRCR	Queen Elizabeth Hospital	Birmingham, West Midlands, UK	Investigator	
Peter W	Cooke		FRCS(Urol)	New Cross Hospital	Wolverhampton, West Midlands, UK	Investigator	
Krzysztof	Kadow		FRCS(Urol)	City Hospital	Birmingham, West Midlands, UK	Investigator	
John	Parkin		FRCS(Urol)	City Hospital	Birmingham, West Midlands, UK	Investigator	
Adel	Makar		FRCS(Urol)	Worcestershire Royal Hospital	Worcester, West Midlands, UK	Investigator	
