# Supplementary material for: Selenium and Vitamin E for Prevention of Non–Muscle-Invasive Bladder Cancer Recurrence and Progression: A Randomized Clinical Trial
Source: JAMA Netw Open. 2023 Oct 17;6(10):e2337494. doi: 10.1001/jamanetworkopen.2023.37494 (PMC10582794; doi:10.1001/jamanetworkopen.2023.37494)
Supplement: Supplement 4. — Data Sharing Statement [file jamanetwopen-e2337494-s004.pdf]

Data Sharing Statement

Bryan. Selenium and Vitamin E for Prevention of Non–Muscle-Invasive Bladder Cancer Recurrence and Progression. *JAMA Netw Open*. Published October 17, 2023. doi:10.1001/jamanetworkopen.2023.37494

Data
Data available: Yes
Data types: Other (please specify)
Additional Information: Participant data and the associated supporting documentation will be available within six months after the publication of this manuscript. Details of our data request process is available on the CRCTU website. Only scientifically sound proposals from appropriately qualified research groups will be considered for data sharing. The decision to release data will be made by the CRCTU Director's Committee, who will consider the scientific validity of the request, the qualifications and resources of the research group, the views of the Chief Investigator and the trial steering committee, consent arrangements, the practicality of anonymising the requested data and contractual obligations. A data sharing agreement will cover the terms and conditions of the release of trial data and will include publication requirements, authorship and acknowledgements and obligations for the responsible use of data. An anonymised encrypted dataset will be transferred directly using a secure method and in accordance with the University of Birmingham's IT guidance on encryption of data sets.
How to access data: Participant data and the associated supporting documentation will be available within six months after the publication of this manuscript. Details of our data request process is available on the CRCTU website. Only scientifically sound proposals from appropriately qualified research groups will be considered for data sharing. The decision to release data will be made by the CRCTU Director's Committee, who will consider the scientific validity of the request, the qualifications and resources of the research group, the views of the Chief Investigator and the trial steering committee, consent arrangements, the practicality of anonymising the requested data and contractual obligations. A data sharing agreement will cover the terms and conditions of the release of trial data and will include publication requirements, authorship and acknowledgements and obligations for the responsible use of data. An anonymised encrypted dataset will be transferred directly using a secure method and in accordance with the University of Birmingham's IT guidance on encryption of data sets.
When available: beginning date: 12-01-2023

Supporting Documents
Document types: Informed consent form, Statistical/analytic code
How to access documents: Study protocol (including statistical analysis plan and informed consent form) is available here: https://www.birmingham.ac.uk/Documents/college-mds/cancer-genomic-sciences/Bladder-Cancer-Prognosis-Programme-SELENIB-Trial.pdf
When available: With publication

Additional Information
Who can access the data: Participant data and the associated supporting documentation will be available within six months after the publication of this manuscript. Details of our data request process is available on the CRCTU website. Only scientifically sound proposals from appropriately qualified research groups will be considered for data sharing. The decision to release data will be made by the CRCTU Director's Committee, who will consider the scientific validity of the request, the qualifications and resources of the research group, the views of the Chief Investigator and the trial steering committee, consent arrangements, the practicality of anonymising the requested data and contractual obligations. A data sharing agreement will cover the terms and conditions of the release of trial data and will include publication requirements, authorship and acknowledgements and obligations for the responsible use of

data. An anonymised encrypted dataset will be transferred directly using a secure method and in accordance with the University of Birmingham's IT guidance on encryption of data sets.
Types of analyses: Participant data and the associated supporting documentation will be available within six months after the publication of this manuscript. Details of our data request process is available on the CRCTU website. Only scientifically sound proposals from appropriately qualified research groups will be considered for data sharing. The decision to release data will be made by the CRCTU Director's Committee, who will consider the scientific validity of the request, the qualifications and resources of the research group, the views of the Chief Investigator and the trial steering committee, consent arrangements, the practicality of anonymising the requested data and contractual obligations. A data sharing agreement will cover the terms and conditions of the release of trial data and will include publication requirements, authorship and acknowledgements and obligations for the responsible use of data. An anonymised encrypted dataset will be transferred directly using a secure method and in accordance with the University of Birmingham's IT guidance on encryption of data sets.
Mechanisms of data availability: Participant data and the associated supporting documentation will be available within six months after the publication of this manuscript. Details of our data request process is available on the CRCTU website. Only scientifically sound proposals from appropriately qualified research groups will be considered for data sharing. The decision to release data will be made by the CRCTU Director's Committee, who will consider the scientific validity of the request, the qualifications and resources of the research group, the views of the Chief Investigator and the trial steering committee, consent arrangements, the practicality of anonymising the requested data and contractual obligations. A data sharing agreement will cover the terms and conditions of the release of trial data and will include publication requirements, authorship and acknowledgements and obligations for the responsible use of data. An anonymised encrypted dataset will be transferred directly using a secure method and in accordance with the University of Birmingham's IT guidance on encryption of data sets.
